# Supplementary material for: Remodeling the cellular stress response for enhanced genetic code expansion in mammalian cells
Source: Nat Commun. 2023 Oct 31;14:6931. doi: 10.1038/s41467-023-42689-2 (PMC10616097; doi:10.1038/s41467-023-42689-2)
Supplement: Supplementary file 1 — Supplementary Information [file 41467_2023_42689_MOESM1_ESM.pdf]

Remodeling the cellular stress response for enhanced genetic code expansion in mammalian cells

Mikhail E. Sushkin[1,2], Christine Koehler[1,3], Edward A. Lemke[1,4]*

[1] Biocentre, Johannes Gutenberg University Mainz, Hanns-Dieter-Hüsch-Weg 17, 55128 Mainz, Germany

[2] International PhD Programme of the Institute of Molecular Biology gGmbH, Hanns-Dieter-Hüsch-Weg 17, 55128 Mainz, Germany

[3] VERAXA Biotech GmbH, Carl-Friedrich-Gauß-Ring 5, 69124 Heidelberg, Germany

[4] Institute of Molecular Biology gGmbH, Ackermannweg 4, 55128 Mainz, Germany

These authors contributed equally: Mikhail E. Sushkin, Christine Koehler.

* Email: edlemke@uni-mainz.de

Supplementary Information

Table of contents

Supplementary Note 1 (page 2)

Supplementary Note 2 (pages 2-3)

Supplementary Note 3 (page 3)

Supplementary Figures 1-50 (pages 4-61)

Supplementary Table 1 (pages 62-63)

Supplementary References (page 63)

Supplementary Note 1

Testing different variants of eIF2α as a potential stress remodeler

One of the strategies to enhance GCE via remodeling the cellular stress response implies an addition of eIF2α S51A remodeler, which does not have the site for phosphorylation at position 51 and which competes with endogenous eIF2α in potential phosphorylation reactions, thus leading to the lower level of phosphorylated eIF2α and the lower performance of cellular stress response. We tested different variants of eIF2α to enhance GCE in mammalian cells (see also Main text). Addition of eIF2α S51A allowed to achieve 1.4-fold increase in GCE efficiency (Fig. 1d and Supplementary Fig. 1). Interestingly, eIF2α wild type (WT) also gave a low level of GCE efficiency enhancement. We suppose that such improvement occurs due to the additional formation of eIF2 complexes caused by eIF2α WT overexpression. Human eIF2α (UniProt P05198) contains proline at position 2, meaning that the base at position +4 in the eIF2α ORF is cytosine but not guanine, as recommended by the vertebrate Kozak sequence[1] (GCCRCCATGG, G at position +4 is underlined). In an attempt to increase the expression level of eIF2α variants and GCE performance, we inserted an additional glycine (GGC codon) at position 2 of eIF2α and repeated the tests. Interestingly, modified (+Gly) eIF2α variants led to worse performance than the original eIF2α WT and eIF2α S51A (Supplementary Figs. 1, 4).

Supplementary Note 2

Testing the integrated stress response (ISR) status with Western blot analysis

To check how introduction of stress remodelers influences the ISR in GCE-performing cells, we evaluated two central parameters of the ISR action – level of phosphorylated eIF2α (P-eIF2α) and global protein synthesis rate. The P-eIF2α level was assessed with anti-phospho-eIF2α antibodies, and puromycin incorporation assay was applied to compare overall protein synthesis rates[2–4]. Puromycin is a translation inhibitor that gets incorporated into the nascent proteins, and application of anti-puromycin antibodies allows to evaluate global protein synthesis rate[5]. Introduction of stress remodelers aims to reduce cellular stress response, and therefore a decrease in eIF2α phosphorylation level and an increase in protein synthesis rate are expected. Indeed, the P-eIF2α level was diminished after addition of PKRΔ, which is in line with the previous studies and with our results where introduction of PKRΔ provided higher overall protein expression and higher GCE efficiency (Supplementary Figs. 16a, 16c). Implementation of eIF2Bγ + eIF2Bε stress remodeler also enabled to achieve lower P-eIF2α level. Such an effect potentially can be explained by higher formation of eIF2B complex, which competes with eIF2α kinases for interaction with eIF2α, and therefore higher amount of eIF2B complexes drags eIF2 complex out of the phosphorylation reaction towards the promotion of translation initiation (Supplementary Figs. 16b, 16d). We could not observe a reduction in eIF2α phosphorylation for samples where eIF2α S51A was overexpressed (stress remodelers eIF2α S51A and PKRΔ + eIF2α S51A v2) probably due to crosstalk between anti-phospho-eIF2α antibodies and non-phosphorylated eIF2α (Supplementary Fig. 17). Puromycin incorporation assay did not show a drastic change in global protein synthesis rate for samples with single introduced stress remodelers, however addition of the PKRΔ + eIF2α S51A v2, which was the most efficient stress remodeler in flow cytometry (FC) tests, provided a significant increase in

protein synthesis rate. We speculate that sensitivity of puromycin incorporation assay in applied conditions might not be enough to detect major changes in overall protein synthesis rate due to short incubation time with puromycin and transient multi-plasmid transfection system.

Supplementary Note 3

Membrane-targeting signals used to direct OT organelles to distinct cellular membranes

To direct the organelles to specific cellular membranes, the following spatial targeting signals are used:

- the N-terminal domain of the rodent LCK tyrosine kinase, amino acids 1-10 (for PMP organelles),
- the N-terminal domain of human EBAG9, amino acids 1-29 (GMP organelles),
- the N-terminal domain of rabbit cytochrome P450 2C1 (CYPIIC1), amino acids 1-27 (ERMP organelles),
- the N-terminal domain of human TOM20, amino acids 1-70 (OMMP organelles).

a

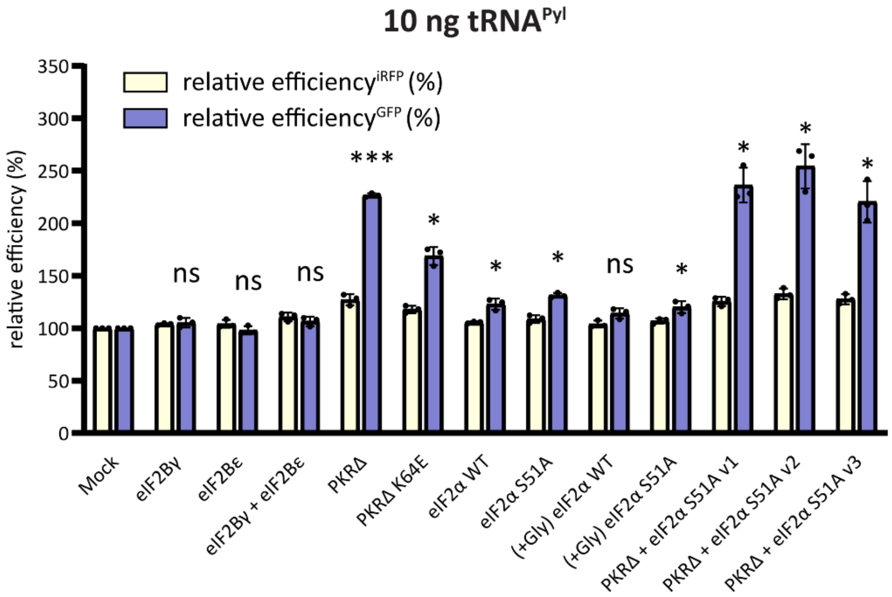
10 ng tRNA^Pyl
relative efficiency^iRFP (%)
relative efficiency^GFP (%)
relative efficiency (%)
0
50
100
150
200
250
300
350
ns
ns
ns
***
*
*
*
ns
*
*
*
*
Mock
eIF2Bγ
eIF2Bε
eIF2Bγ + eIF2Bε
PKRΔ
PKRΔ K64E
eIF2α WT
eIF2α S51A
(+Gly) eIF2α WT
(+Gly) eIF2α S51A
PKRΔ + eIF2α S51A v1
PKRΔ + eIF2α S51A v2
PKRΔ + eIF2α S51A v3

b

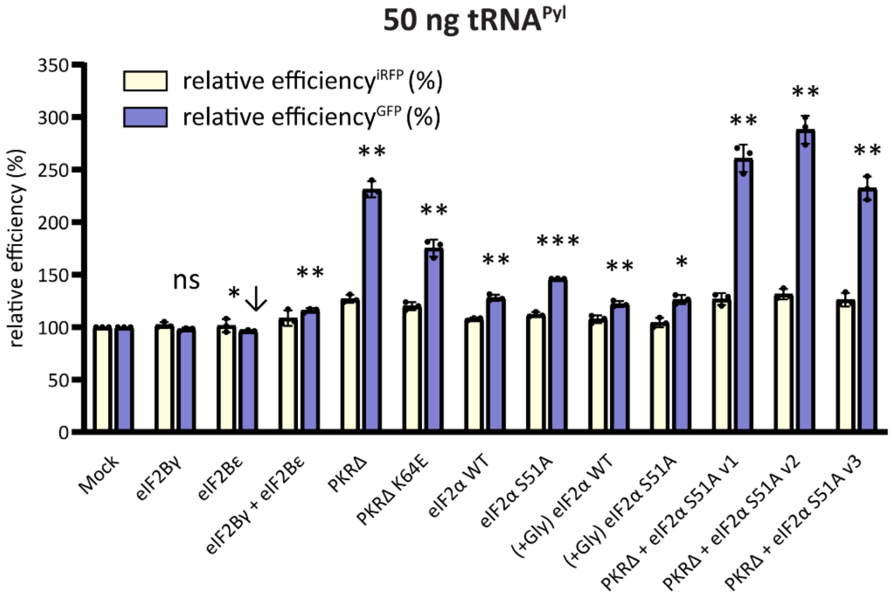
50 ng tRNA^Pyl
relative efficiency^iRFP (%)
relative efficiency^GFP (%)
relative efficiency (%)
0
50
100
150
200
250
300
350
ns
*
**
**
**
**
***
**
*
**
**
**
Mock
eIF2Bγ
eIF2Bε
eIF2Bγ + eIF2Bε
PKRΔ
PKRΔ K64E
eIF2α WT
eIF2α S51A
(+Gly) eIF2α WT
(+Gly) eIF2α S51A
PKRΔ + eIF2α S51A v1
PKRΔ + eIF2α S51A v2
PKRΔ + eIF2α S51A v3

c

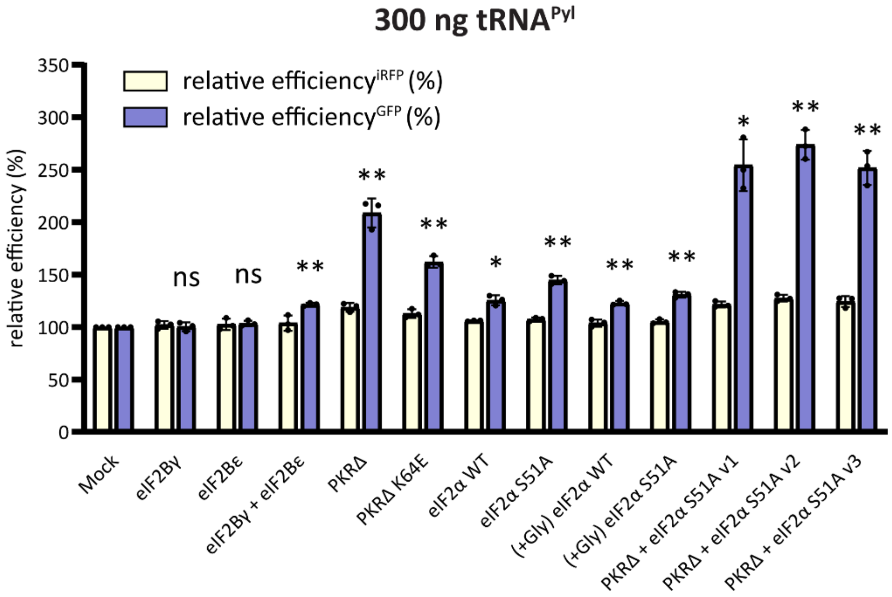
300 ng tRNA^Pyl
relative efficiency^iRFP (%)
relative efficiency^GFP (%)
relative efficiency (%)
0
50
100
150
200
250
300
350
ns
ns
**
**
**
*
**
**
**
*
**
**
Mock
eIF2Bγ
eIF2Bε
eIF2Bγ + eIF2Bε
PKRΔ
PKRΔ K64E
eIF2α WT
eIF2α S51A
(+Gly) eIF2α WT
(+Gly) eIF2α S51A
PKRΔ + eIF2α S51A v1
PKRΔ + eIF2α S51A v2
PKRΔ + eIF2α S51A v3

Supplementary Figure 1: Screening of various stress remodelers for studying the effect of their addition on GCE efficiency and iRFP expression level. Bar plots show changes in fluorescent signal in units of relative efficiency^{GFP} (%) and relative efficiency^{iRFP} (%) in presence of 10 ng tRNA^{Pyl} (**a**), 50 ng tRNA^{Pyl} (**b**), or 300 ng tRNA^{Pyl} (**c**) after addition of potential stress remodelers. Relative efficiency^{GFP} (%) is calculated as the median GFP signal for each particular case divided by the median GFP signal for samples with the addition of mock plasmid. Relative efficiency^{iRFP} (%) is calculated as the median iRFP signal for each particular case divided by the median iRFP signal for samples with the addition of mock plasmid. Median GFP and iRFP signals were obtained after FC analysis of corresponding samples. Bar plots show the mean value for relative efficiencies of three independent experiments, error bars represent the standard deviation (SD). Adjusted *p* values are presented for relative efficiency^{GFP} (%) values. Ns denotes not significant (p value > 0.05), * - p value ≤ 0.05, ** - p value ≤ 0.01, *** - p value ≤ 0.001, p values were calculated using one-sample (two-tailed) t-test with Benjamini-Hochberg multiple comparisons correction (false discovery rate = 0.05). *↓ designates p value ≤ 0.05 and significant decrease in relative efficiency^{GFP} (%) to compare with Mock (no stress remodeler addition). Exact p values are provided in the Source Data file. Data for stress remodelers eIF2Bγ + eIF2Bε, PKRΔ, eIF2α S51A, and PKRΔ + eIF2α S51A v2 are also presented in Fig. 1d.

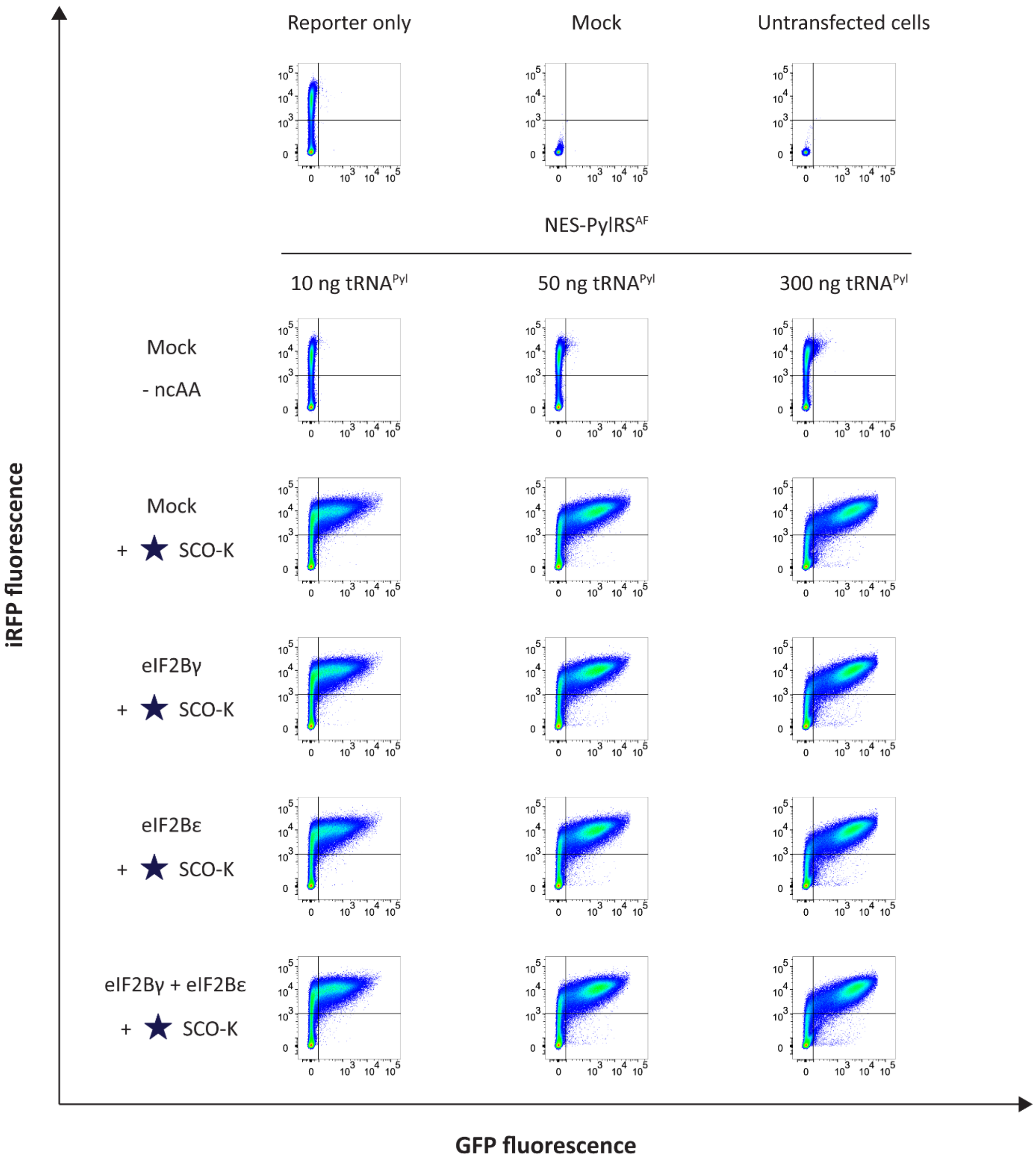

Supplementary Figure 2: FC analysis of iRFP-GFP^{39TAG} reporter in case of absence or presence of stress remodeler eIF2Bγ, eIF2Bε, or their combination. Concatenated data from three independent experiments are shown.

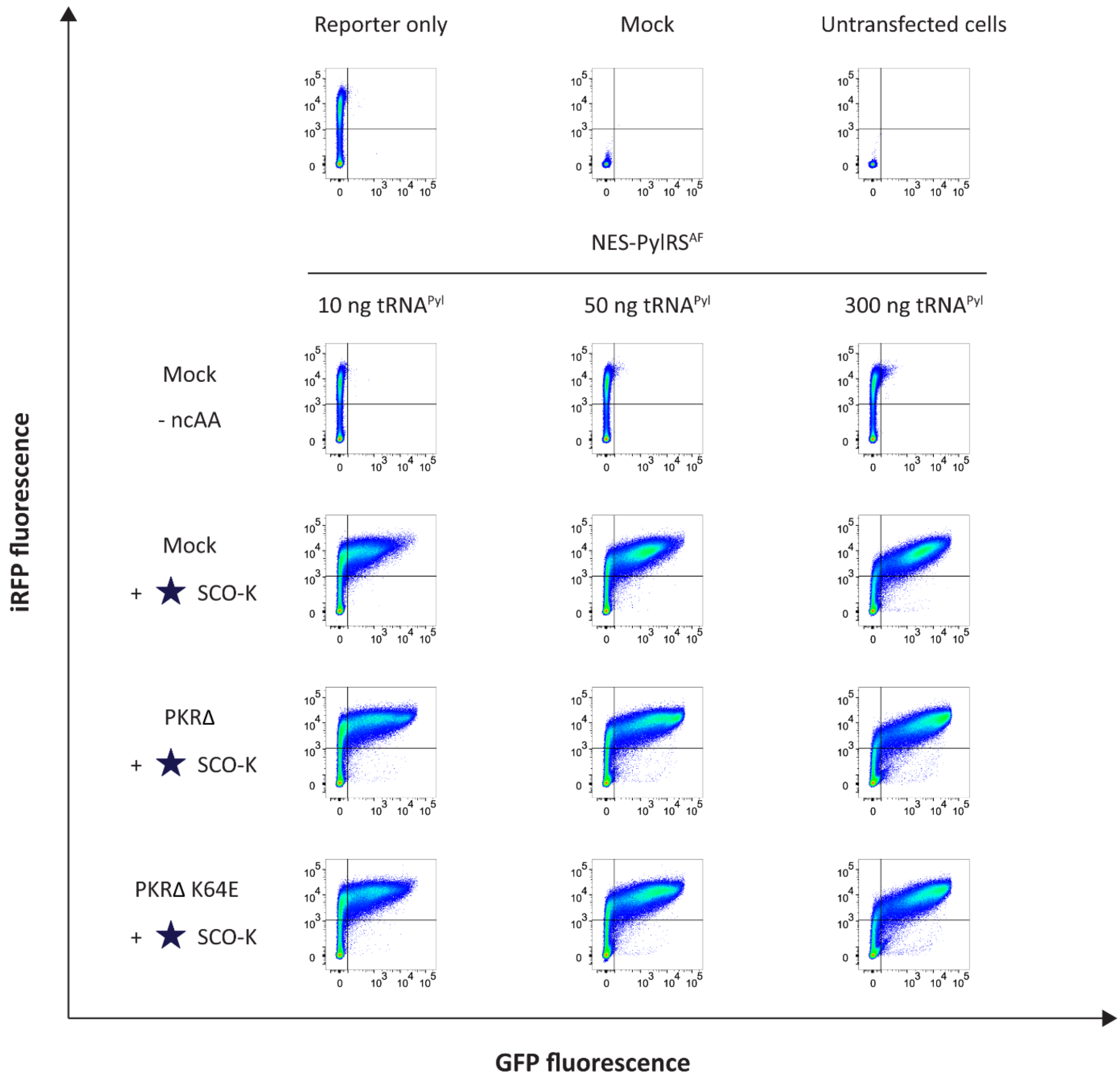

Supplementary Figure 3: FC analysis of iRFP-GFP^{39TAG} reporter in case of absence or presence of stress remodeler PKRΔ or PKRΔ K64E. Concatenated data from three independent experiments are shown.

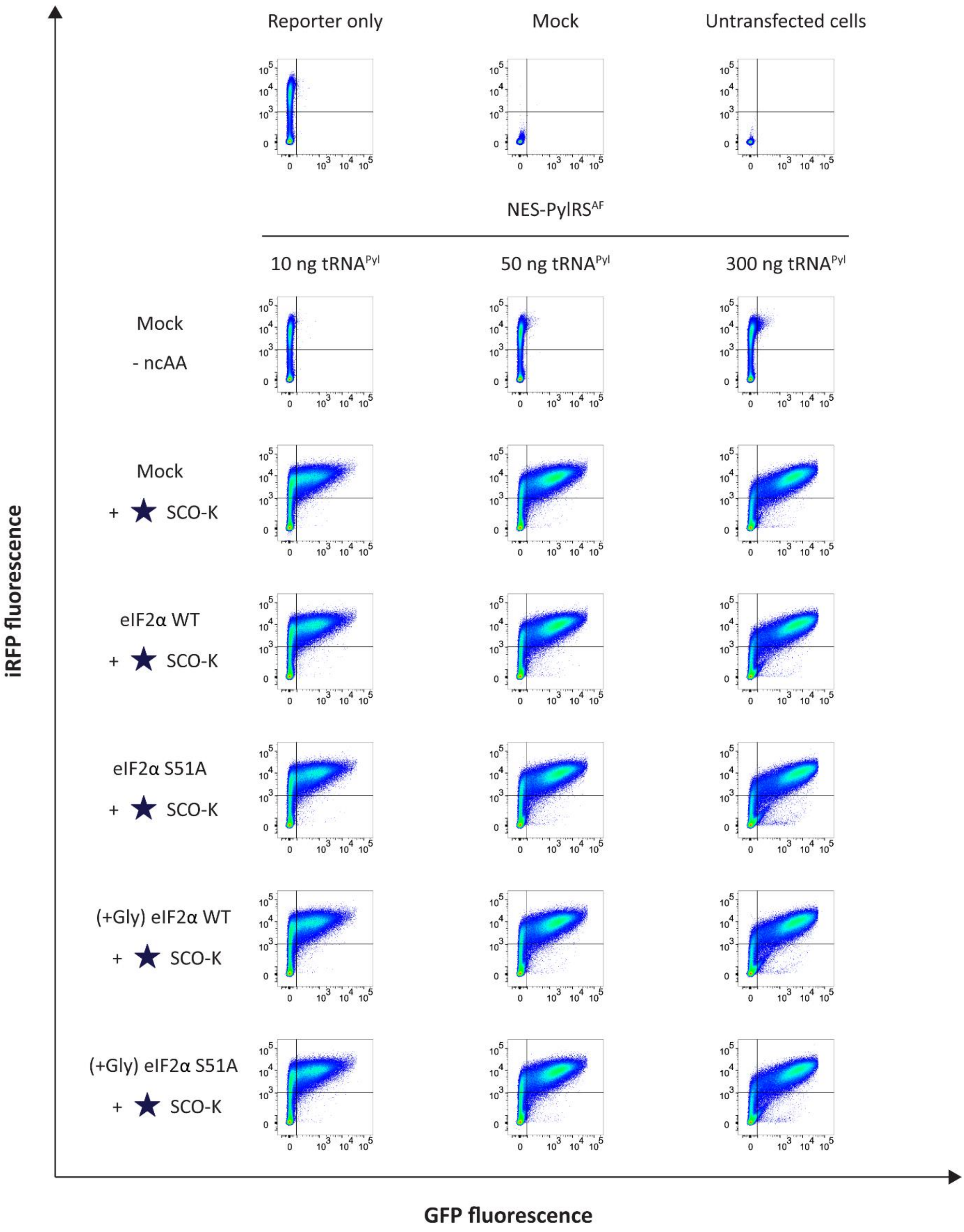

Supplementary Figure 4: FC analysis of iRFP-GFP^{39TAG} reporter in case of absence or presence of stress remodeler eIF2α WT, eIF2α S51A, (+Gly) eIF2α WT, or (+Gly) eIF2α S51A. Concatenated data from three independent experiments are shown.

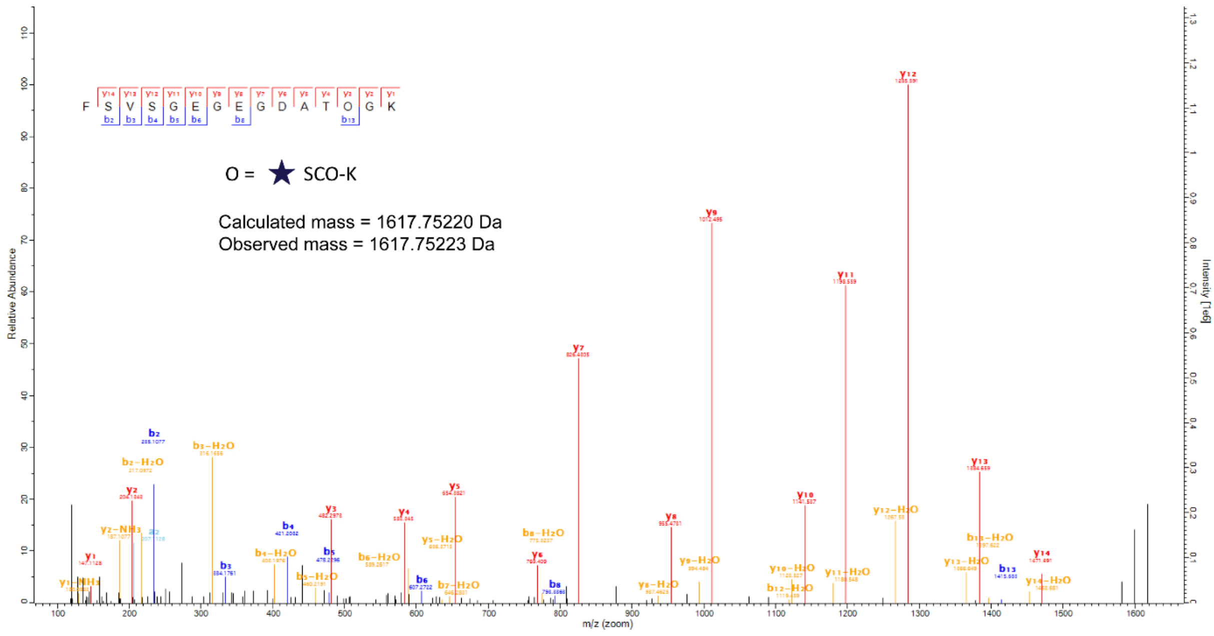

Supplementary Figure 5: MS/MS spectrum of cyclooctyne-lysine (SCO-K)-containing peptide derived after digestion of iRFP-GFP$^{39SCO\text{-}K}$ reporter expressed in HEK293T cells in the absence of stress remodeler. Observed mass denotes the monoisotopic mass of the neutral peptide. Delta between calculated and observed mass is 0.00003. Precursor ion charge is +2.

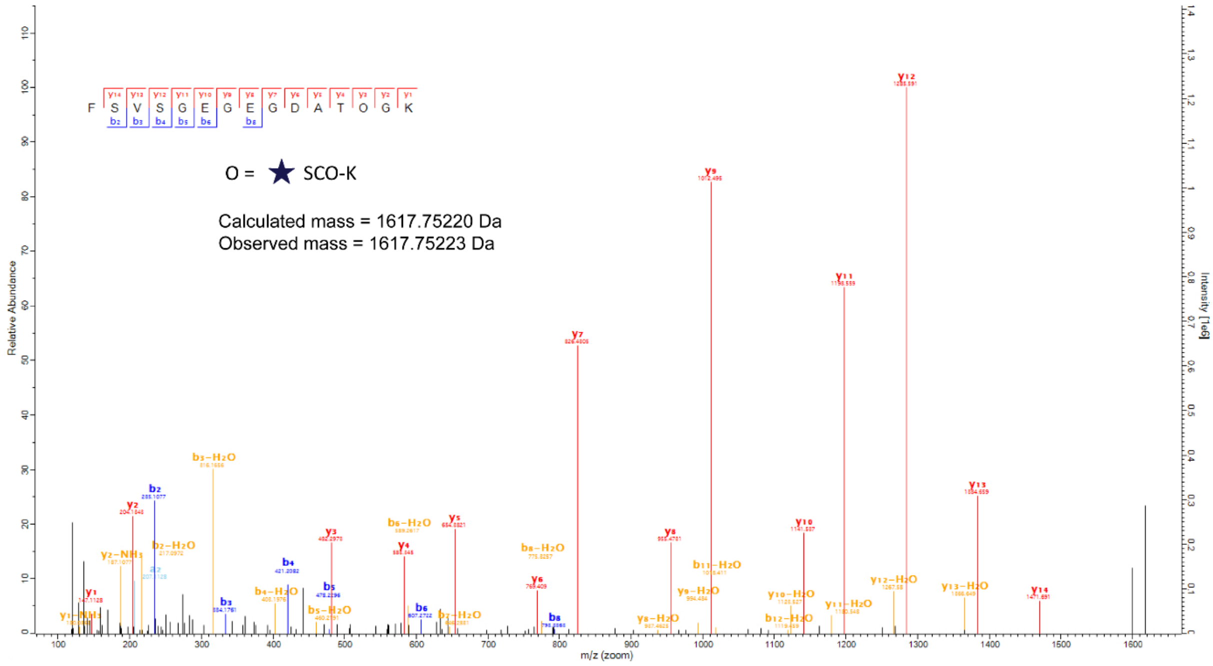

Supplementary Figure 6: MS/MS spectrum of SCO-K-containing peptide derived after digestion of iRFP-GFP$^{39SCO\text{-}K}$ reporter expressed in HEK293T cells in the presence of eIF2Bγ + eIF2Bε. Observed mass denotes the monoisotopic mass of the neutral peptide. Delta between calculated and observed mass is 0.00003. Precursor ion charge is +2.

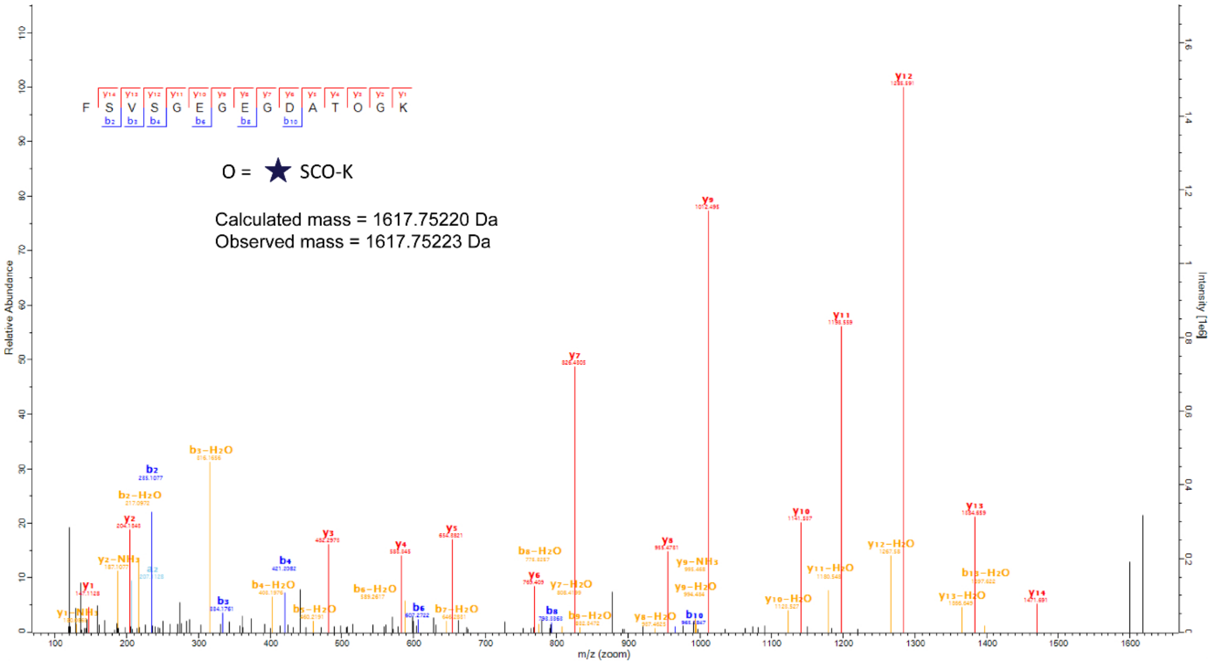

Supplementary Figure 7: MS/MS spectrum of cyclooctyne-lysine SCO-K-containing peptide derived after digestion of iRFP-GFP$^{39SCO\text{-}K}$ reporter expressed in HEK293T cells in the presence of PKRΔ. Observed mass denotes the monoisotopic mass of the neutral peptide. Delta between calculated and observed mass is 0.00003. Precursor ion charge is +2.

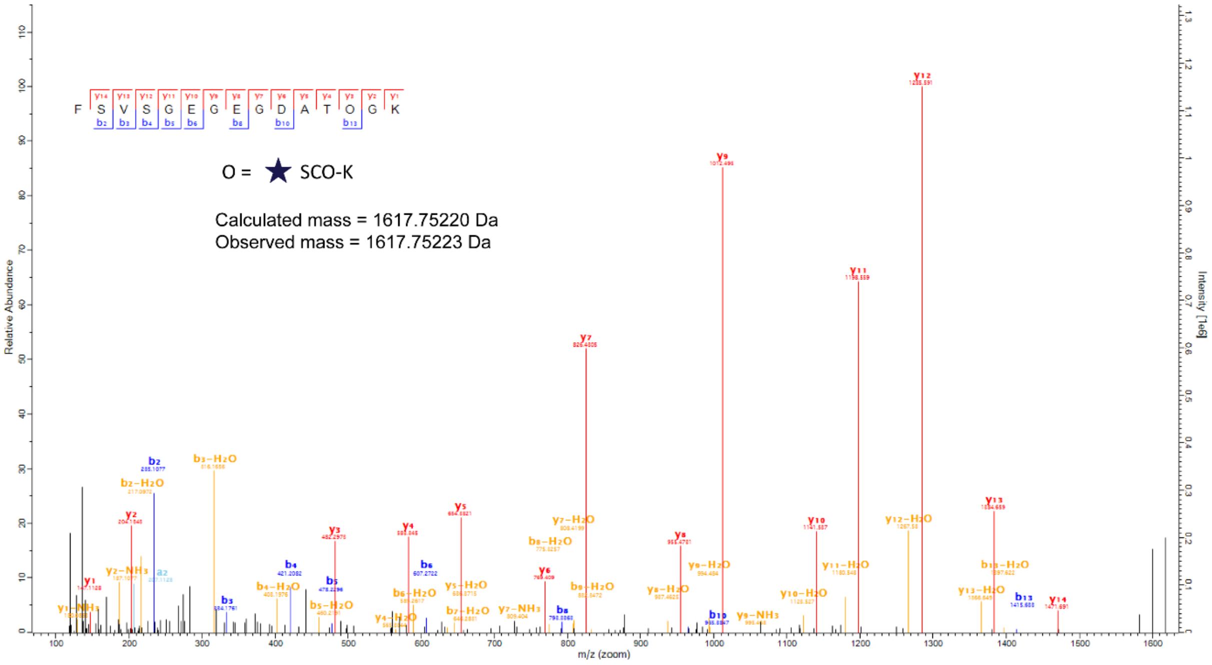

Supplementary Figure 8: MS/MS spectrum of SCO-K-containing peptide derived after digestion of iRFP-GFP$^{39SCO\text{-}K}$ reporter expressed in HEK293T cells in the presence of eIF2α S51A. Observed mass denotes the monoisotopic mass of the neutral peptide. Delta between calculated and observed mass is 0.00003. Precursor ion charge is +2.

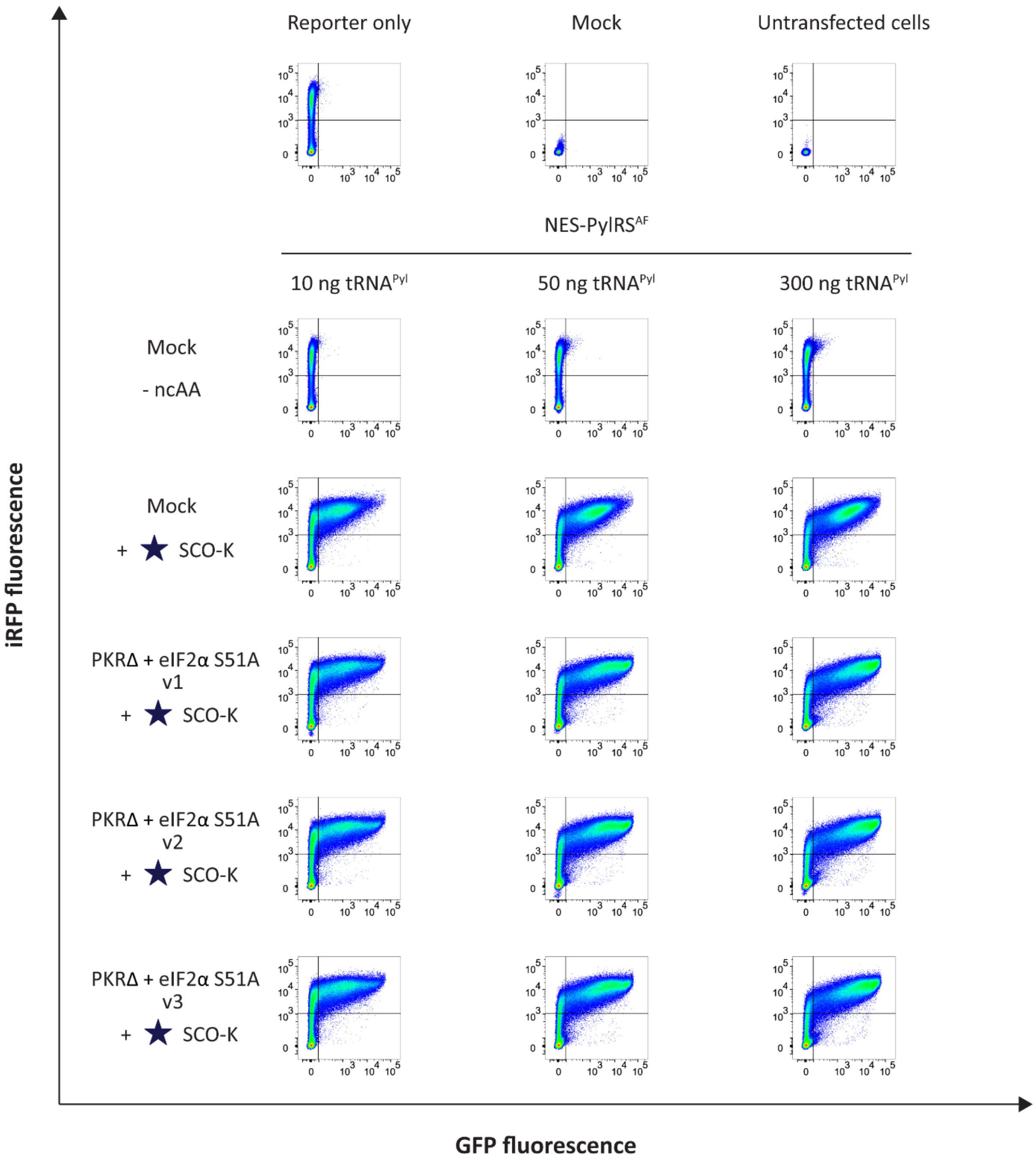

Supplementary Figure 9: FC analysis of iRFP-GFP^{39TAG} reporter in case of absence or presence of stress remodeler PKRΔ + eIF2α S51A v1, PKRΔ + eIF2α S51A v2, or PKRΔ + eIF2α S51A v3. Concatenated data from three independent experiments are shown.

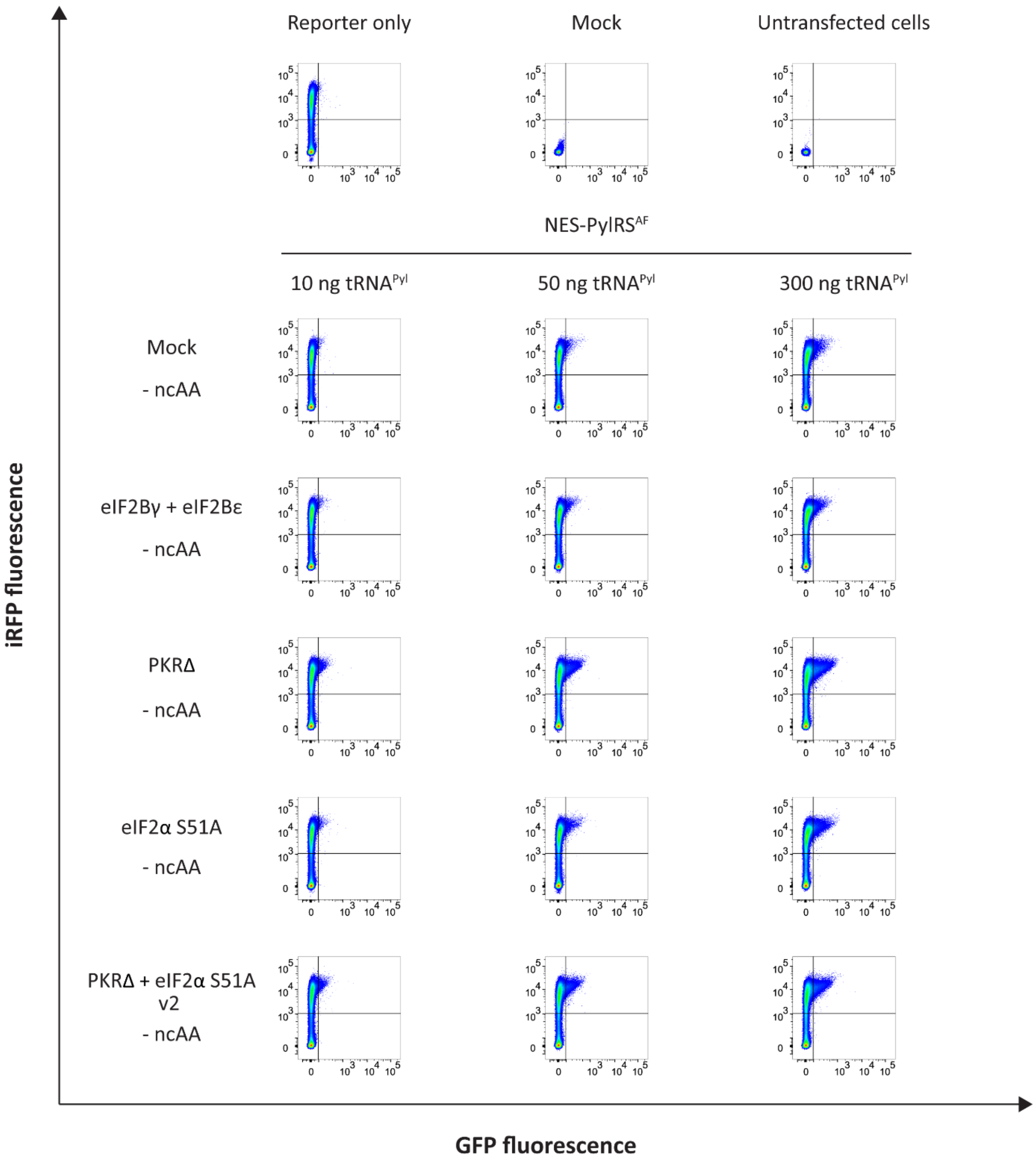

Supplementary Figure 10: FC analysis of iRFP-GFP^{39TAG} reporter in case of ncAA absence and presence of stress remodeler eIF2Bγ + eIF2Bε, PKRΔ, eIF2α S51A, or PKRΔ + eIF2α S51A v2. Concatenated data from three independent experiments are shown.

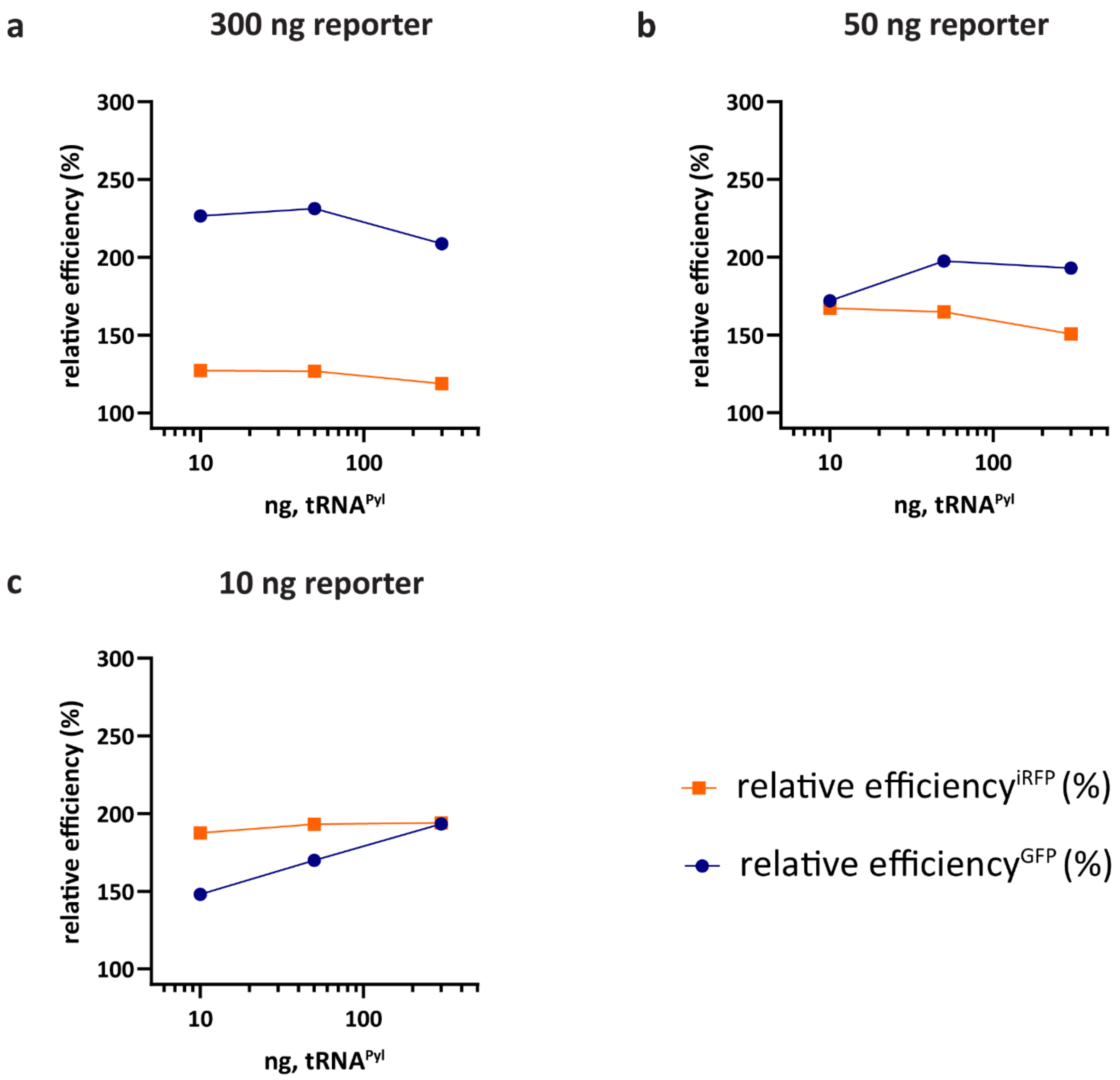

Supplementary Figure 11: Difference in iRFP and GFP fold increase depends on the transfected reporter plasmid mass. The reporter iRFP-GFP^{39TAG} was titrated down from 300 ng (**a**) to 50 ng (**b**), or 10 ng (**c**). Stress remodeler PKRΔ was used to increase GCE efficiency for all samples. Relative efficiency^{GFP} (%) and relative efficiency^{iRFP} (%) were calculated as in Supplementary Fig. 1.

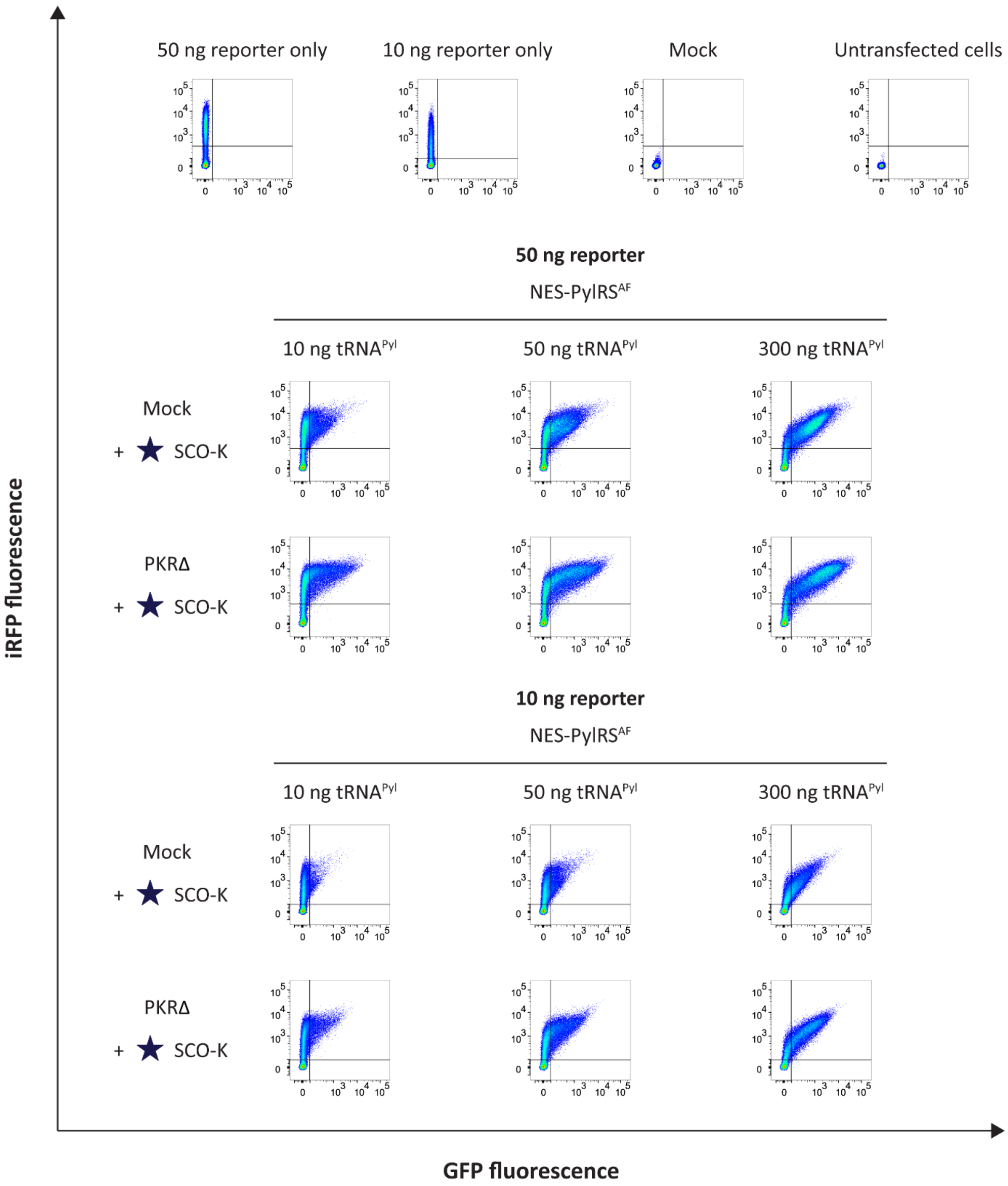

Supplementary Figure 12: FC analysis of titrated iRFP-GFP^{39TAG} reporter in case of absence or presence of stress remodeler PKRΔ. FC plots for one performed biological replicate are shown.

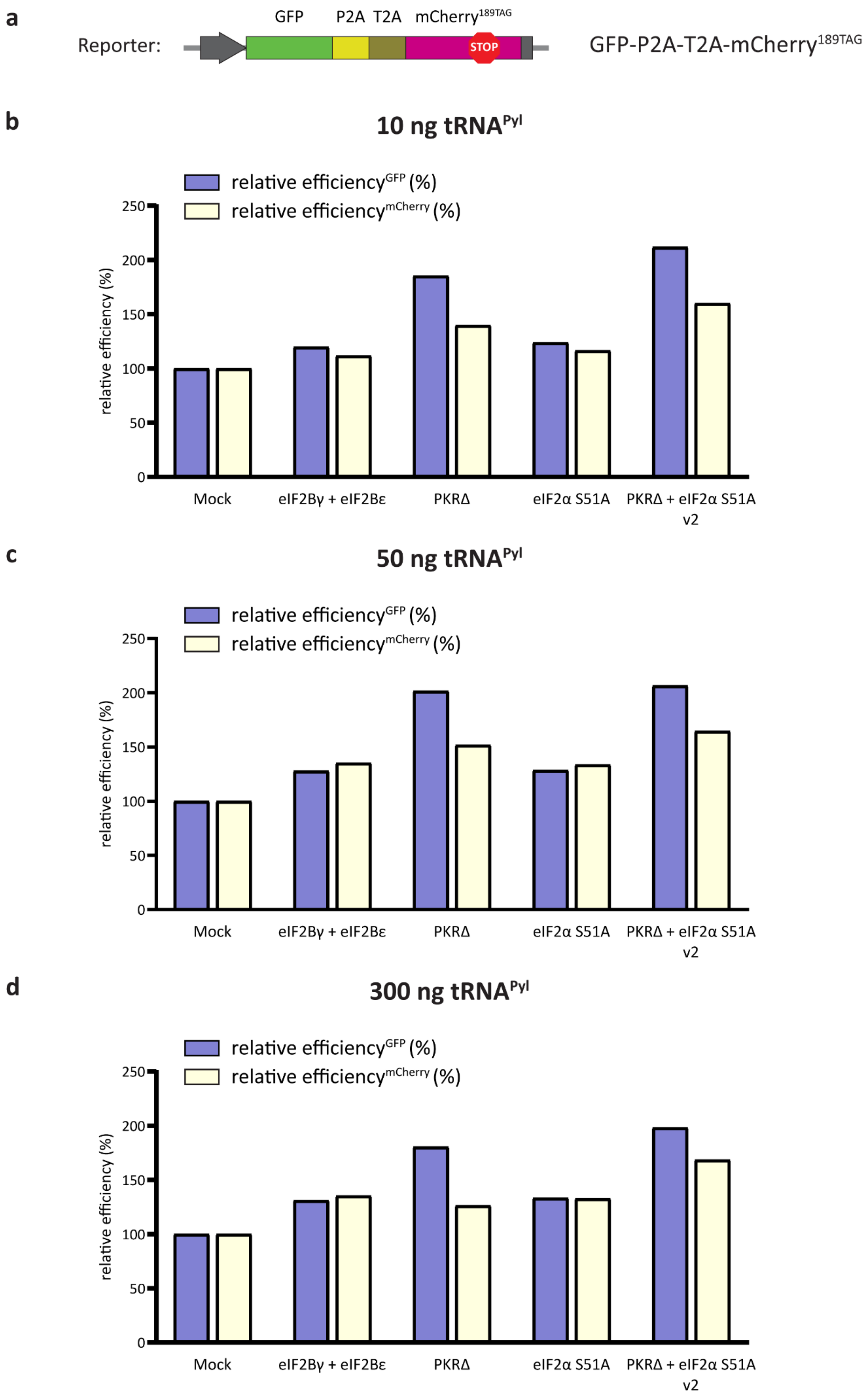
a
Reporter:
GFP
P2A
T2A
mCherry189TAG
STOP
GFP-P2A-T2A-mCherry189TAG
b
10 ng tRNAPyl
relative efficiencyGFP (%)
relative efficiencymCherry (%)
relative efficiency (%)
250
200
150
100
50
0
Mock
eIF2Bγ + eIF2Bε
PKRΔ
eIF2α S51A
PKRΔ + eIF2α S51A v2
c
50 ng tRNAPyl
relative efficiencyGFP (%)
relative efficiencymCherry (%)
relative efficiency (%)
250
200
150
100
50
0
Mock
eIF2Bγ + eIF2Bε
PKRΔ
eIF2α S51A
PKRΔ + eIF2α S51A v2
d
300 ng tRNAPyl
relative efficiencyGFP (%)
relative efficiencymCherry (%)
relative efficiency (%)
250
200
150
100
50
0
Mock
eIF2Bγ + eIF2Bε
PKRΔ
eIF2α S51A
PKRΔ + eIF2α S51A v2

Supplementary Figure 13: Reporter GFP-P2A-T2A-mCherry^{189TAG} shows increase in GFP and mCherry signals. **a**, Schematic representation of GFP-P2A-T2A-mCherry^{189TAG} reporter. Bar plots demonstrate relative efficiencies measured at 10 ng tRNA^{Pyl} (**b**), 50 ng tRNA^{Pyl} (**c**) and 300 ng tRNA^{Pyl} (**d**) in the absence or presence of tested stress remodelers. Relative efficiency^{GFP} (%) is calculated as the median GFP signal for each particular case divided by the median GFP signal for samples with the addition of mock plasmid. Relative efficiency^{mCherry} (%) is calculated as the median mCherry signal for each particular case divided by the median mCherry signal for samples with the addition of mock plasmid. Median GFP and mCherry signals were obtained after FC analysis of corresponding samples.

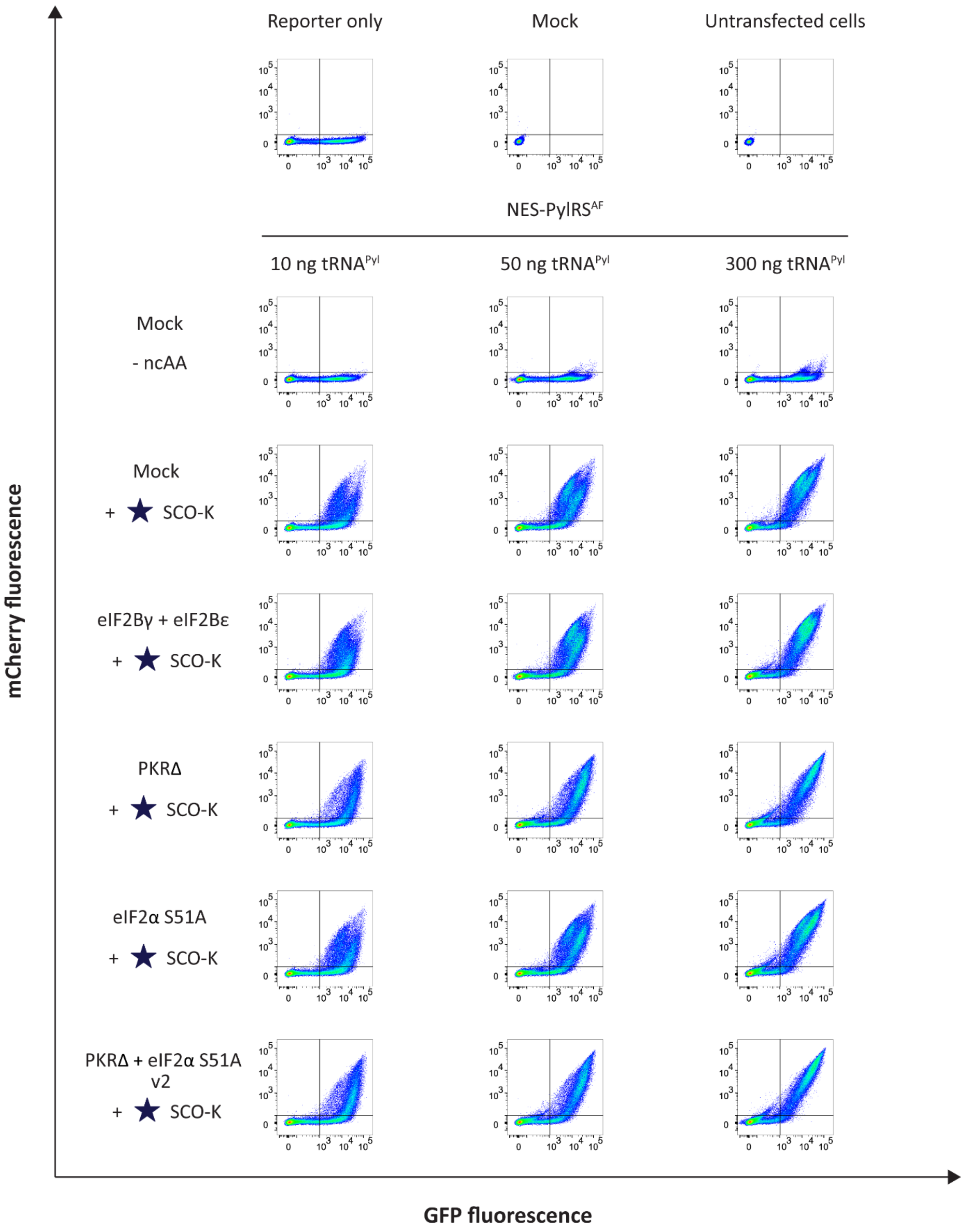

Supplementary Figure 14: FC analysis of GFP-P2A-T2A-mCherry^{189TAG} reporter in case of absence or presence of stress remodeler eIF2Bγ + eIF2Bε, PKRΔ, eIF2α S51A, or PKRΔ + eIF2α S51A v2. FC plots for one performed biological replicate are shown.

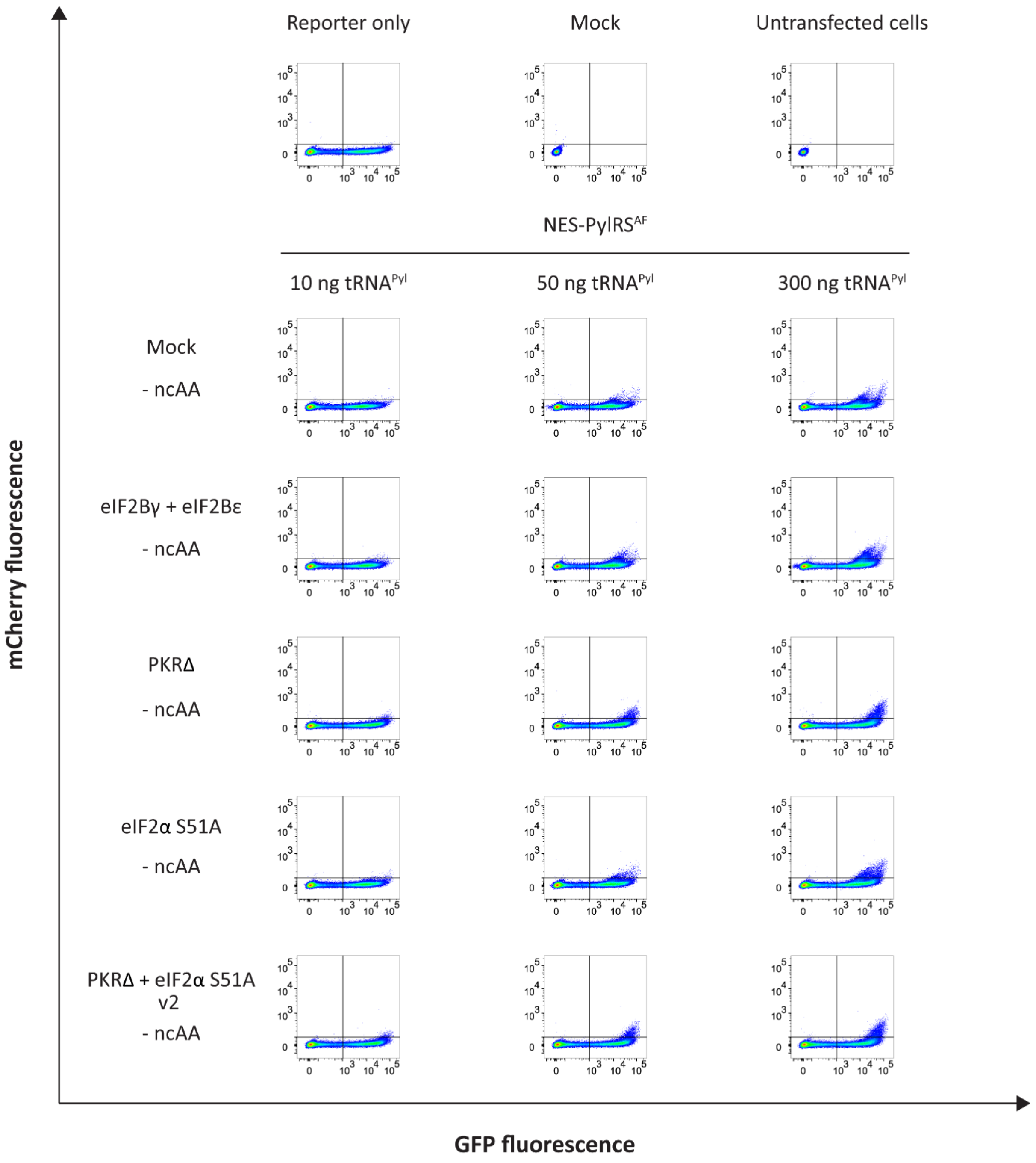

Supplementary Figure 15: FC analysis of GFP-P2A-T2A-mCherry^{189TAG} reporter in case of ncAA absence and presence of stress remodeler eIF2Bγ + eIF2Bε, PKRΔ, eIF2α S51A, or PKRΔ + eIF2α S51A v2. FC plots for one performed biological replicate are shown.

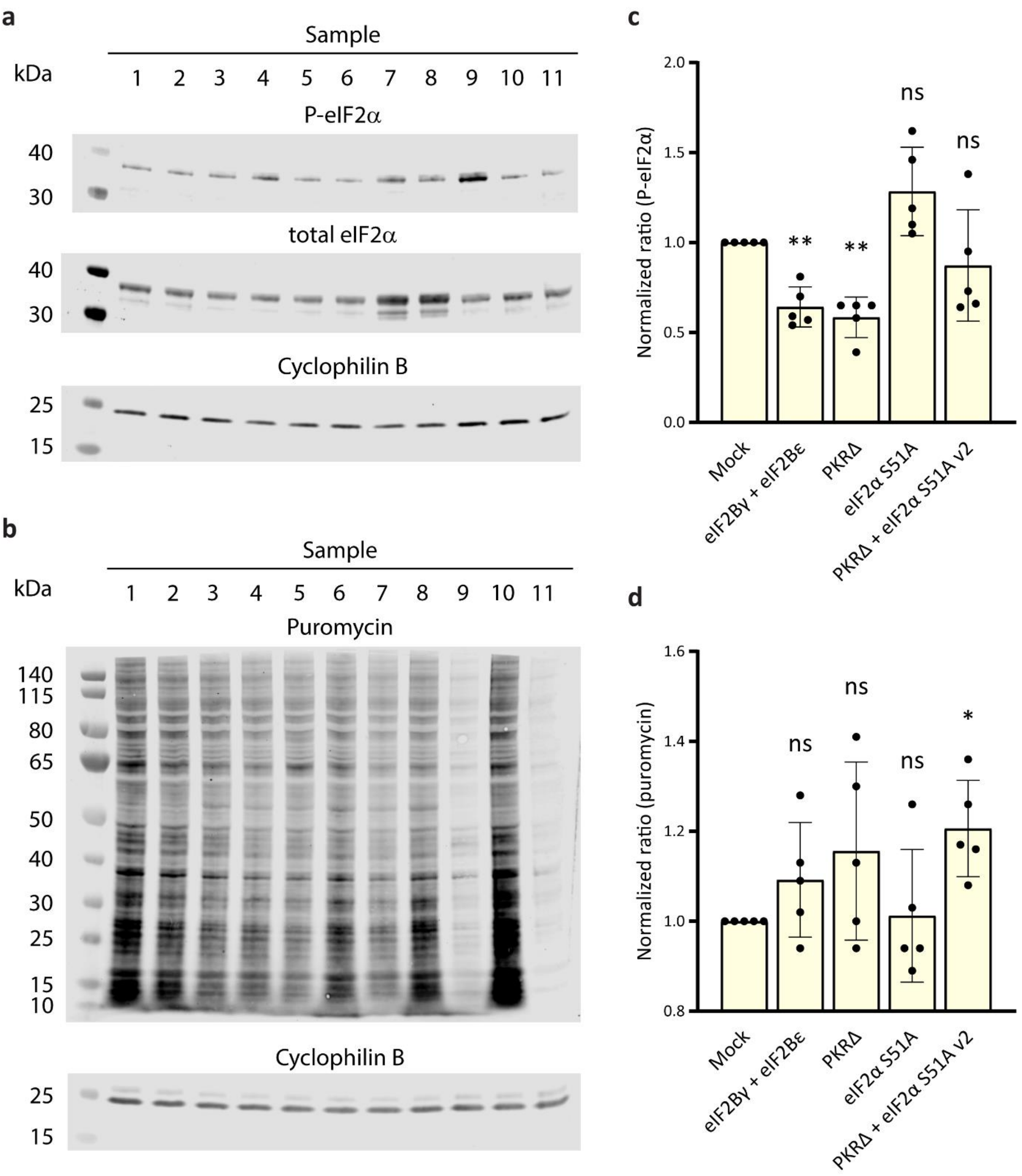

Supplementary Figure 16: Assessment of the ISR status after addition of stress remodelers to GCE-performing cells. a, **b**, Representative Western blots for evaluation of phosphorylated and total eIF2α levels (**a**) and puromycin incorporation (**b**). Cyclophilin B was used as a loading control. Samples: 1) untransfected cells; 2) only Mock (1200 ng plasmid) transfected; 3) GCE (PylRS, $tRNA^{Pyl}$) with mock plasmid (no stress remodeler) supplemented without SCO-K; 4) GCE with mock plasmid (no stress remodeler) supplemented with SCO-K; 5) GCE with eIF2Bγ + eIF2Bε stress remodeler plus SCO-K; 6) GCE with PKRΔ stress remodeler plus SCO-K; 7) GCE with eIF2α S51A stress remodeler plus SCO-K; 8) GCE with PKRΔ + eIF2α S51A v2 stress remodeler plus SCO-K; 9) untransfected cells under stress conditions (0.5 mM sodium arsenite, 1 hour incubation); 10) untransfected cells supplemented with distilled water instead of sodium arsenite; 11) untransfected cells supplemented with water instead of puromycin. **c**, **d**, Bar plots representing normalized ratios calculated after Western blot quantification for assessment of phosphorylated eIF2α level (**c**) and puromycin incorporation (**d**).

Normalized ratio (P-eIF2α) or (puromycin) was defined as P-eIF2α (**c**) or puromycin (**d**) signal divided by cyclophilin B signal for each sample and normalized to the control sample - GCE with no stress remodeler addition (Mock) supplemented with SCO-K (sample 4 on the Western blots). Bar plots show the mean value for normalized ratios of five independent experiments, error bars represent the SD. Ns denotes not significant (p value > 0.05), * - p value ≤ 0.05, ** - p value ≤ 0.01, p values were calculated using one-sample (two-tailed) t-test. Exact p values are provided in the Source Data file.

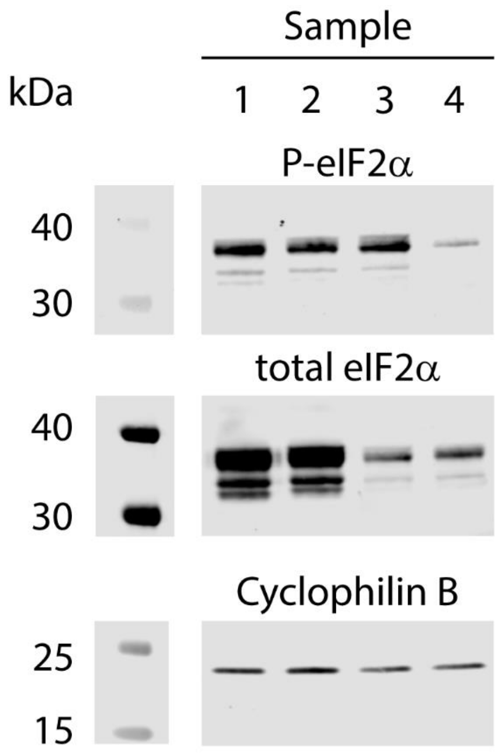

Supplementary Figure 17: Assessment of the ISR status in HEK293T cells transfected with eIF2α S51A-containing stress remodelers and without GCE machinery (PylRS and $tRNA^{Pyl}$). Western blot demonstrates levels of phosphorylated and total eIF2α in tested samples. Cyclophilin B was used as a loading control. Samples: 1) addition of eIF2α S51A stress remodeler; 2) addition of PKRΔ + eIF2α S51A v2 stress remodeler; 3) untransfected cells under stress conditions (0.5 mM sodium arsenite, 1 hour incubation); 4) untransfected cells supplemented with distilled water instead of sodium arsenite.

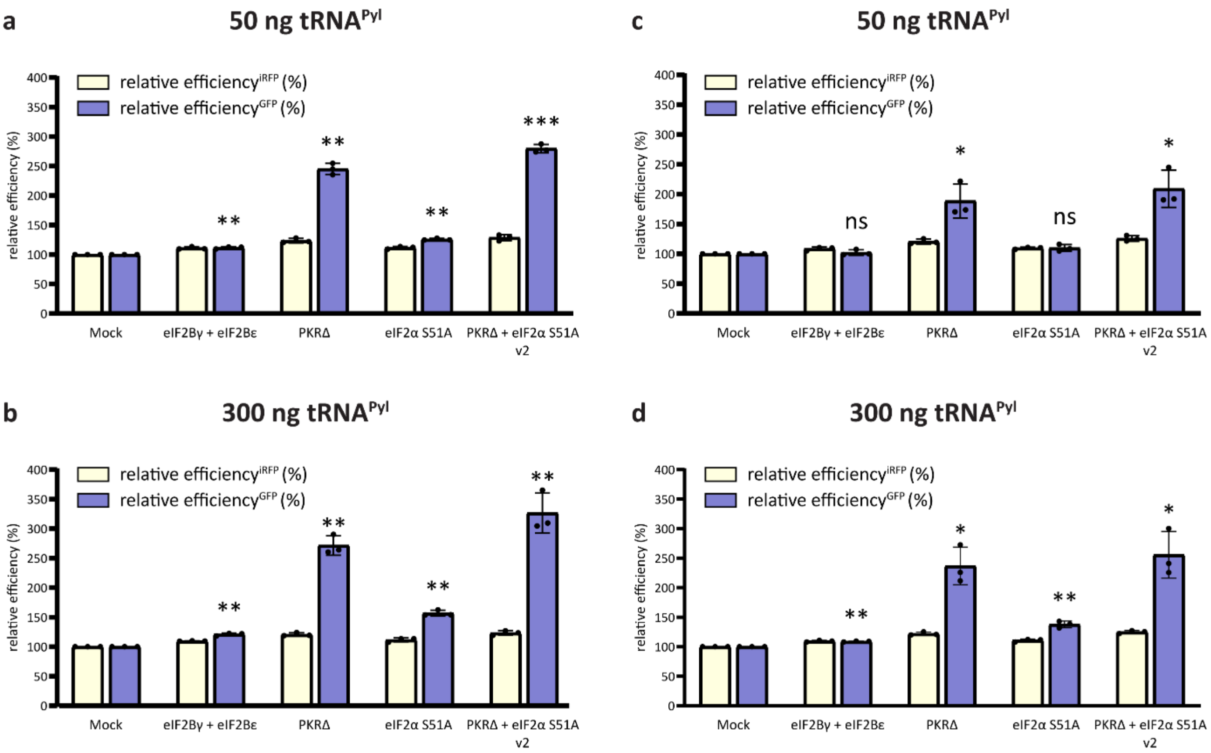

Supplementary Figure 18: Addition of stress remodelers increases the efficiency of multiple ncAA incorporation. Reporter iRFP-GFP^{39TAG,149TAG} (**a**, **b**) or iRFP-GFP^{39TAG,149TAG,182TAG} (**c**, **d**) was used to quantify GCE enhancement obtained after introduction of stress remodelers. Bar plots represent relative efficiencies measured at 50 ng tRNA^{Pyl} (**a**, **c**) and 300 ng tRNA^{Pyl} (**b**, **d**). Relative efficiency^{GFP} (%) is calculated as the median GFP signal for each particular case divided by the median GFP signal for samples with the addition of mock plasmid. Relative efficiency^{iRFP} (%) is calculated as the median iRFP signal for each particular case divided by the median iRFP signal for samples with the addition of mock plasmid. Median GFP and iRFP signals were obtained after FC analysis of corresponding samples. Bar plots show the mean value for relative efficiencies of three independent experiments, error bars represent the SD. *P* values are presented for relative efficiency^{GFP} (%) values. Ns denotes not significant (p value > 0.05), * - p value ≤ 0.05, ** - p value ≤ 0.01, *** - p value ≤ 0.001, p values were calculated using one-sample (two-tailed) t-test.

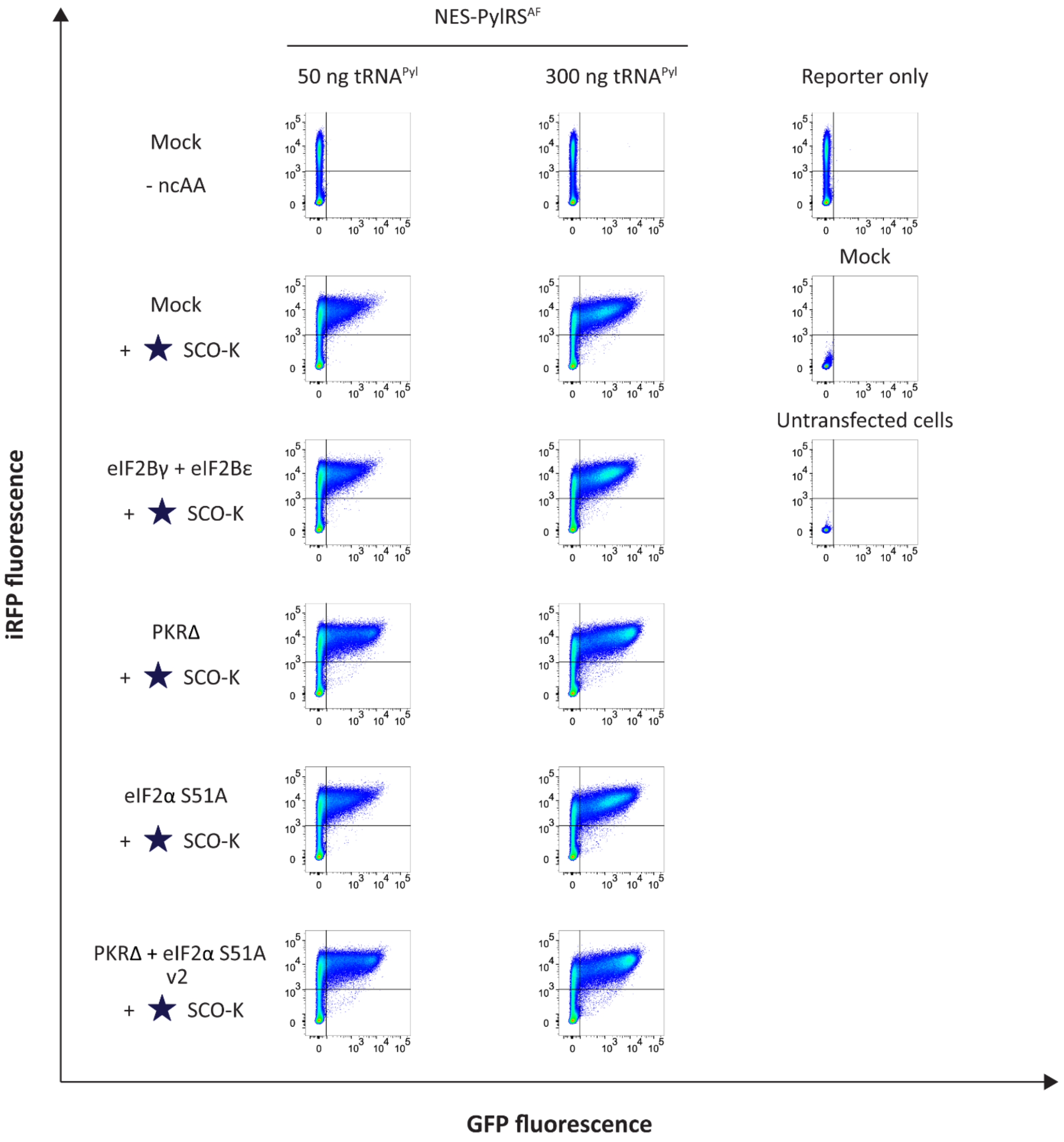

Supplementary Figure 19: FC analysis of iRFP-GFP^{39TAG,149TAG} reporter in case of absence or presence of stress remodeler eIF2Bγ + eIF2Bε, PKRΔ, eIF2α S51A, or PKRΔ + eIF2α S51A v2. Concatenated data from three independent experiments are shown.

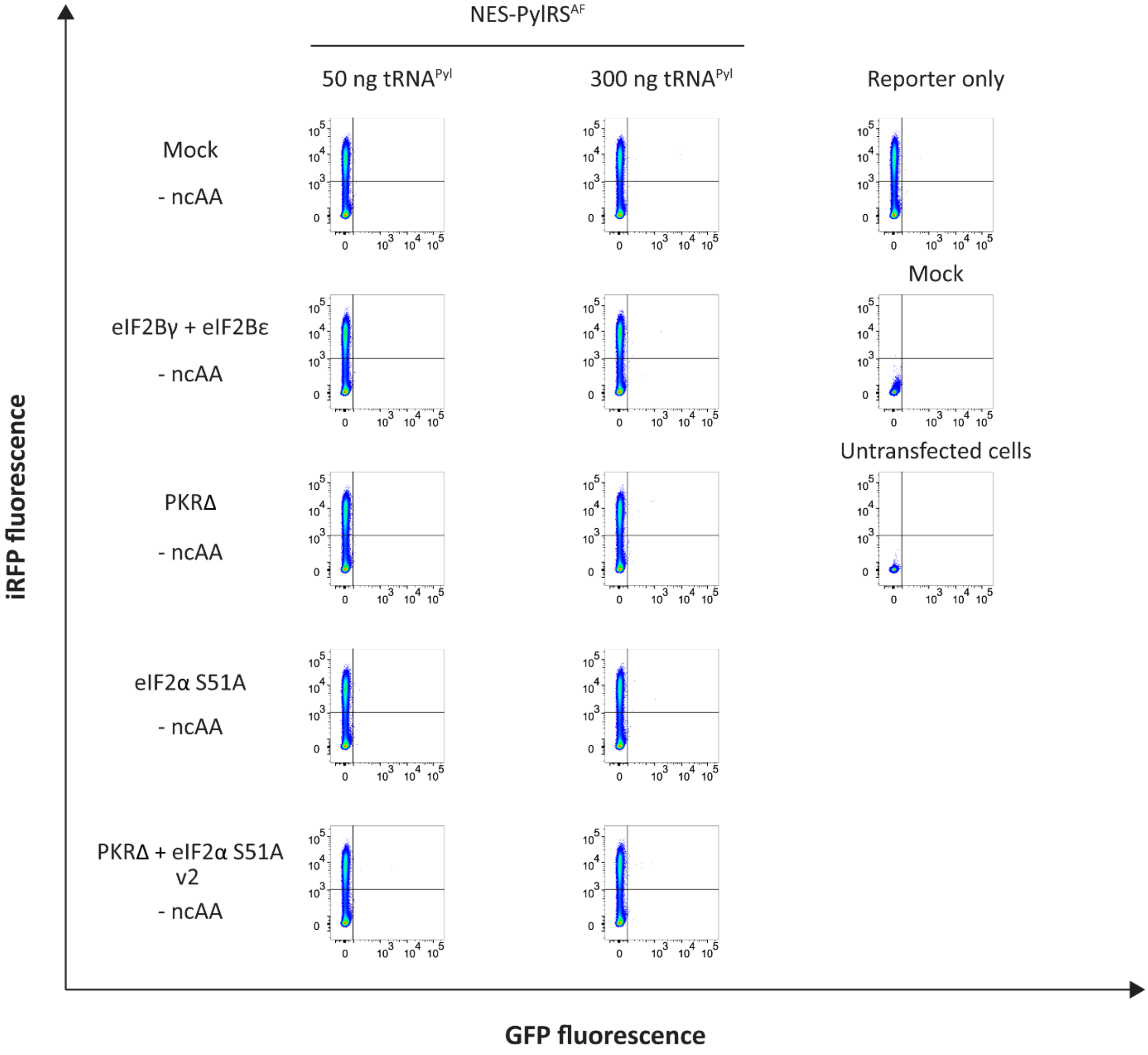

Supplementary Figure 20: FC analysis of iRFP-GFP^{39TAG,149TAG} reporter in case of ncAA absence and presence of stress remodeler eIF2Bγ + eIF2Bε, PKRΔ, eIF2α S51A, or PKRΔ + eIF2α S51A v2. Concatenated data from three independent experiments are shown.

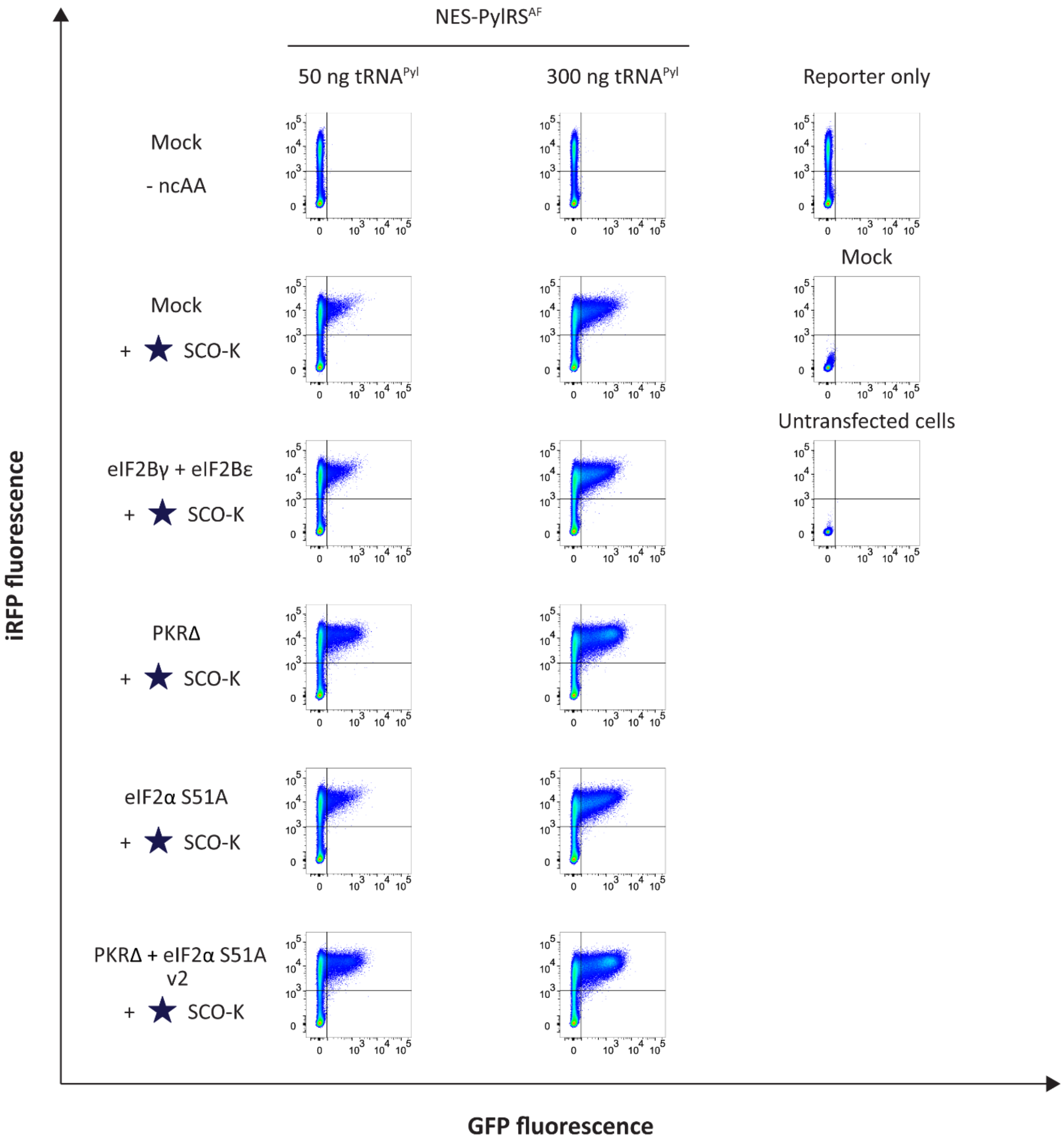

Supplementary Figure 21: FC analysis of iRFP-GFP^{39TAG,149TAG,182TAG} reporter in case of absence or presence of stress remodeler eIF2Bγ + eIF2Bε, PKRΔ, eIF2α S51A, or PKRΔ + eIF2α S51A v2. Concatenated data from three independent experiments are shown.

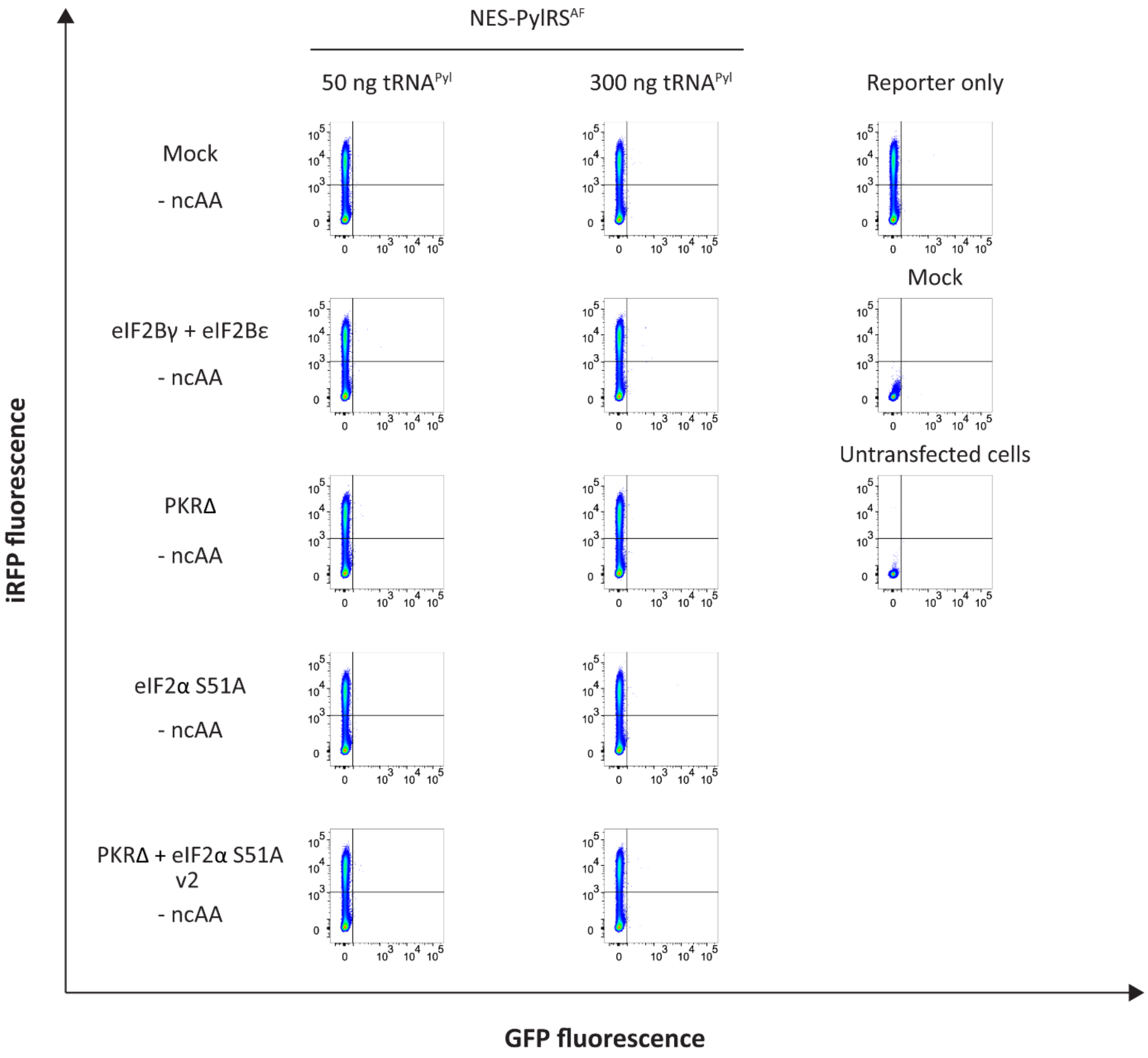

Supplementary Figure 22: FC analysis of iRFP-GFP^{39TAG,149TAG,182TAG} reporter in case of ncAA absence and presence of stress remodeler eIF2Bγ + eIF2Bε, PKRΔ, eIF2α S51A, or PKRΔ + eIF2α S51A v2. Concatenated data from three independent experiments are shown.

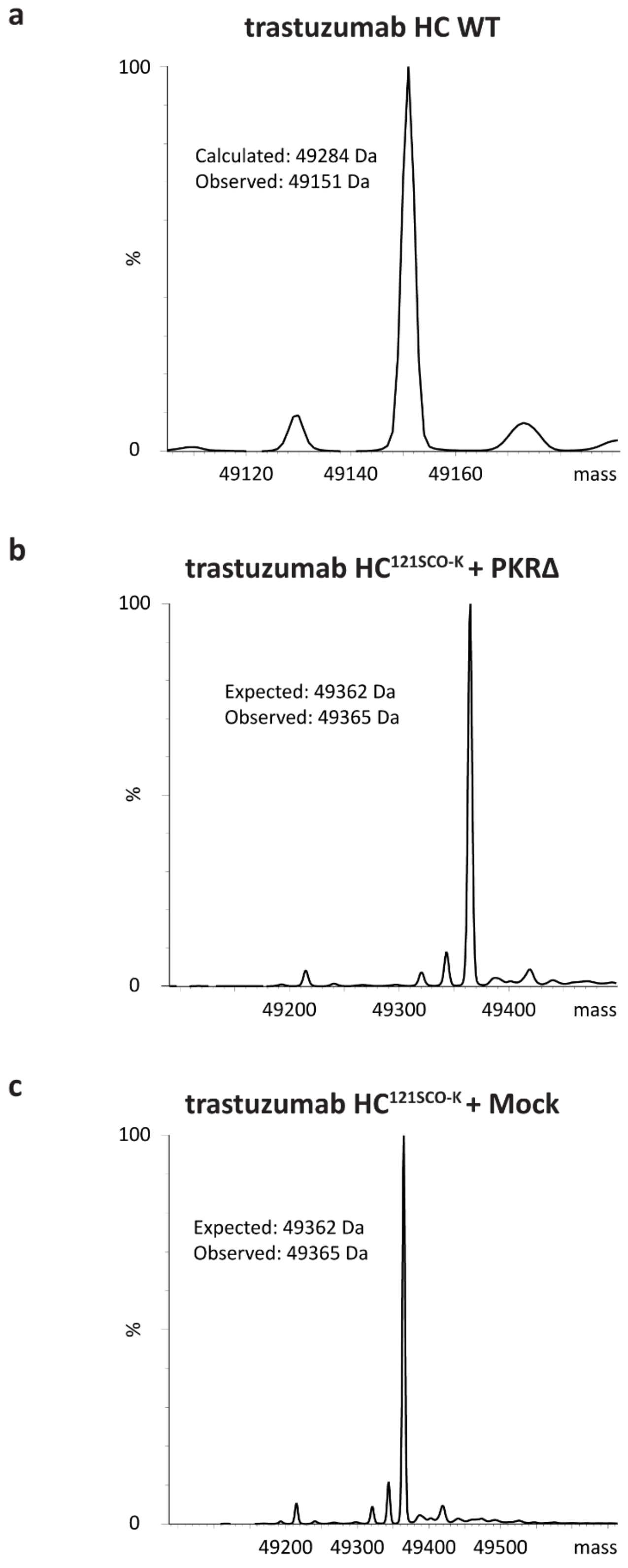

Supplementary Figure 23: MS spectra of trastuzumab HC WT and trastuzumab HC$^{121SCO\text{-}K}$ showing the intact mass of the protein. Trastuzumab WT (trastuzumab LC + HC, without stop codon) was expressed to set up a control for MS analysis (**a**). Trastuzumab$^{121SCO\text{-}K}$ (trastuzumab LC + HC$^{121SCO\text{-}K}$) was expressed in the presence of PKRΔ (**b**) or without a stress remodeler (**c**). Full-length antibody for all samples was purified, deglycosylated and submitted to MS analysis (see Methods section). The figure

represents only MS analysis of trastuzumab HC WT (**a**) and trastuzumab $HC^{121SCO\text{-}K}$ (**b, c**). Calculated mass of trastuzumab HC WT is 49284 Da, observed mass of trastuzumab HC WT is 49151 Da, delta is -133 Da due to loss of first amino acid E (129 Da) (**a**). Based on the assumption of loss of E amino acid, the expected mass of trastuzumab $HC^{121SCO\text{-}K}$ is 49362 Da, the observed mass of trastuzumab $HC^{121SCO\text{-}K}$ is 49365 Da for both (**b**) and (**c**), delta is +3 Da.

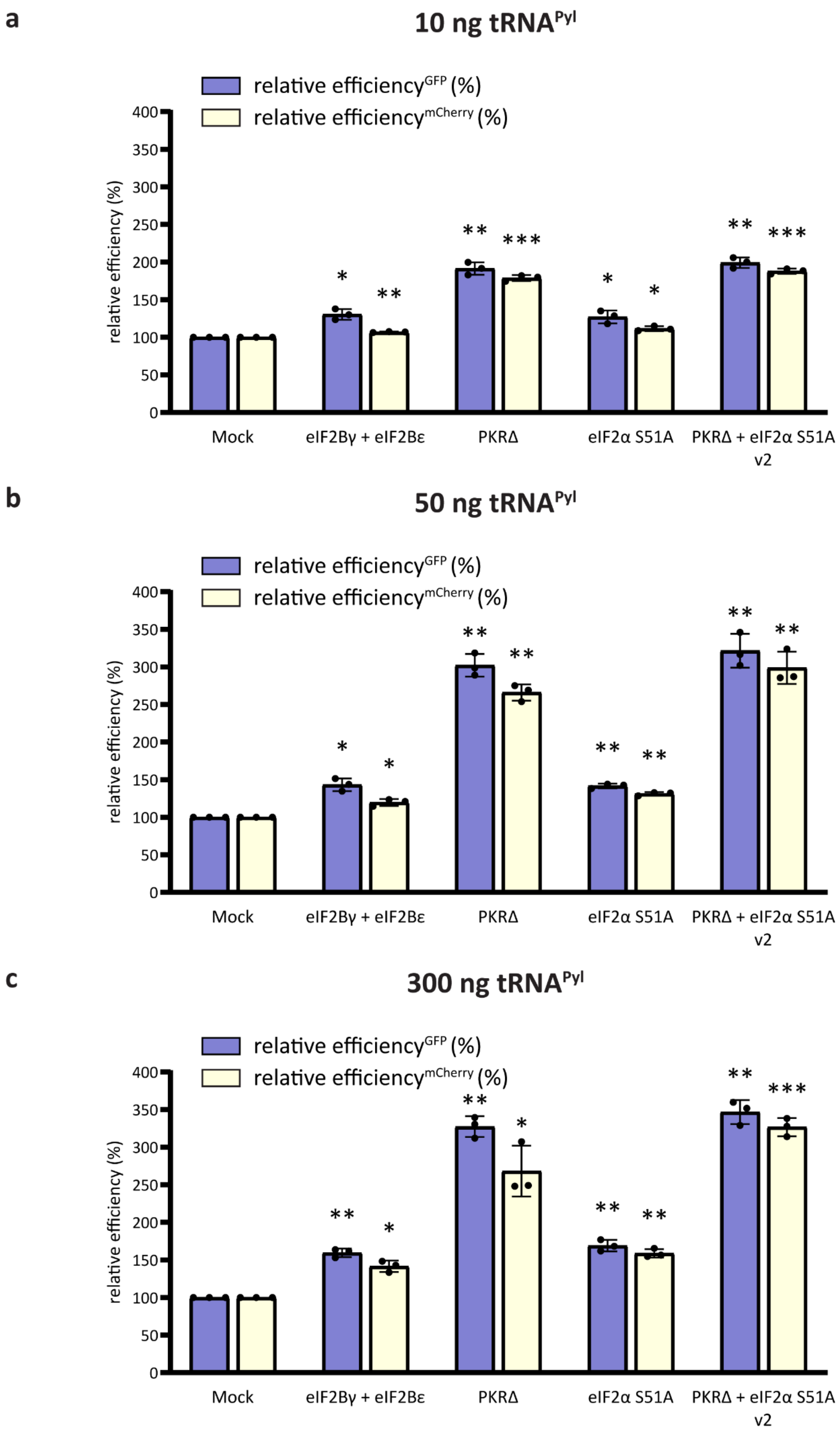
a
10 ng tRNA^Pyl
relative efficiency^GFP (%)
relative efficiency^mCherry (%)
relative efficiency (%)
0
50
100
150
200
250
300
350
400
Mock
eIF2Bγ + eIF2Bε
PKRΔ
eIF2α S51A
PKRΔ + eIF2α S51A v2
b
50 ng tRNA^Pyl
c
300 ng tRNA^Pyl

Supplementary Figure 24: Implementation of stress remodelers enhances GCE in case of applying GFP^{39TAG}, mCherry^{189TAG}-ms2 reporter. Bar plots show an increase in fluorescent signal in units of relative efficiency (%) in presence of 10 ng tRNA^{Pyl} (**a**), 50 ng tRNA^{Pyl} (**b**), or 300 ng tRNA^{Pyl} (**c**) and NES-PylRS^{AF} after addition of tested stress remodelers. Relative efficiency^{GFP} (%) is calculated as the median GFP signal for each particular case divided by the median GFP signal for NES-PylRS^{AF} with the addition of mock plasmid. Relative efficiency^{mCherry} (%) is calculated as the median mCherry signal for each particular case divided by the median mCherry signal for NES-PylRS^{AF} with the addition of mock plasmid. Median GFP or mCherry signals were obtained after FC analysis of corresponding samples. Bar plots show the mean value for relative efficiencies of three independent experiments, error bars represent the SD. * denotes p value ≤ 0.05, ** - p value ≤ 0.01, *** - p value ≤ 0.001, p values were calculated using one-sample (two-tailed) t-test. Exact p values are provided in the Source Data file.

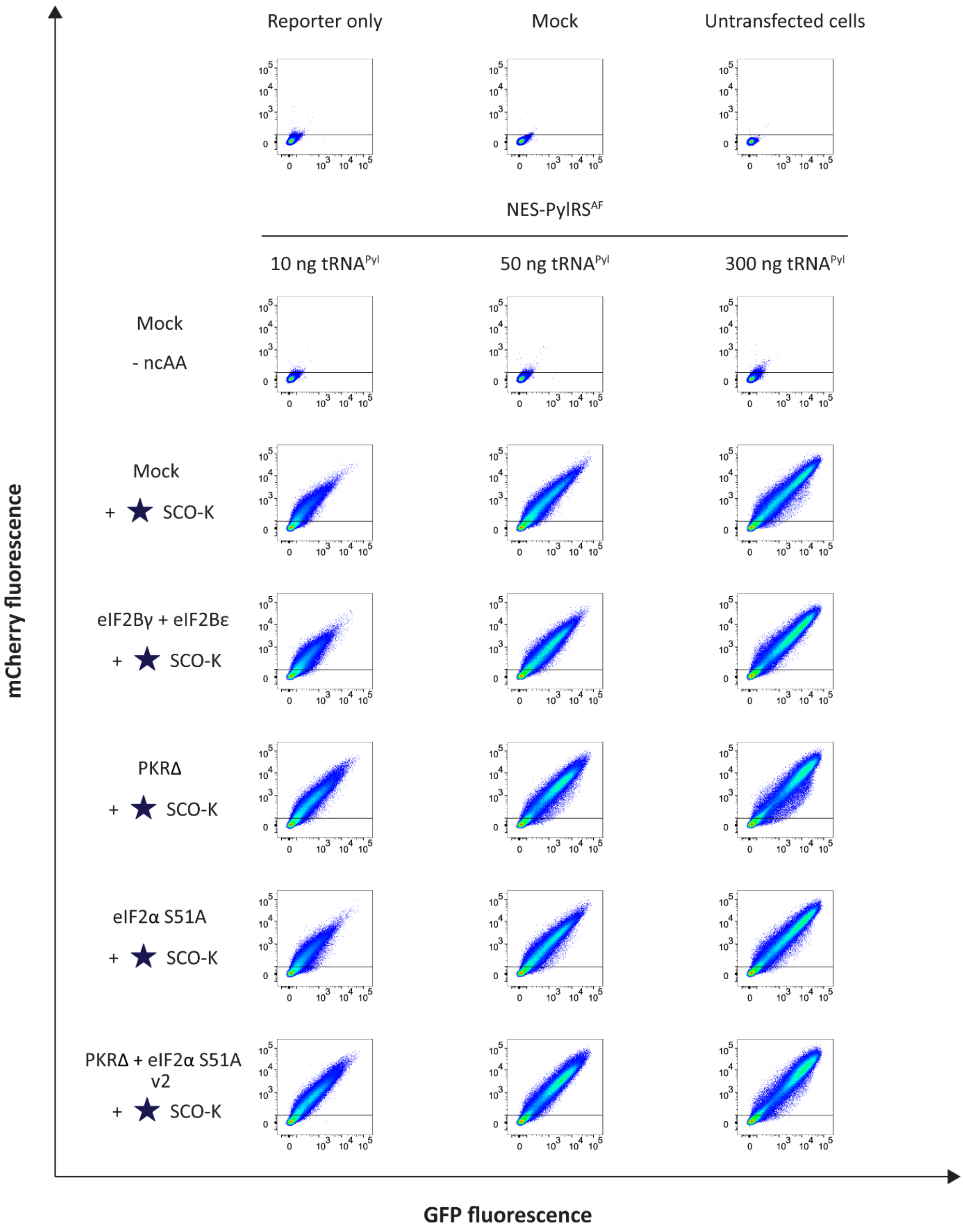

Supplementary Figure 25: FC analysis of GFP^{39TAG}, mCherry^{189TAG}-ms2 reporter showing NES-PylRS^{AF} performance in case of absence or presence of stress remodeler eIF2Bγ + eIF2Bε, PKRΔ, eIF2α S51A, or PKRΔ + eIF2α S51A v2. Concatenated data from three independent experiments are shown.

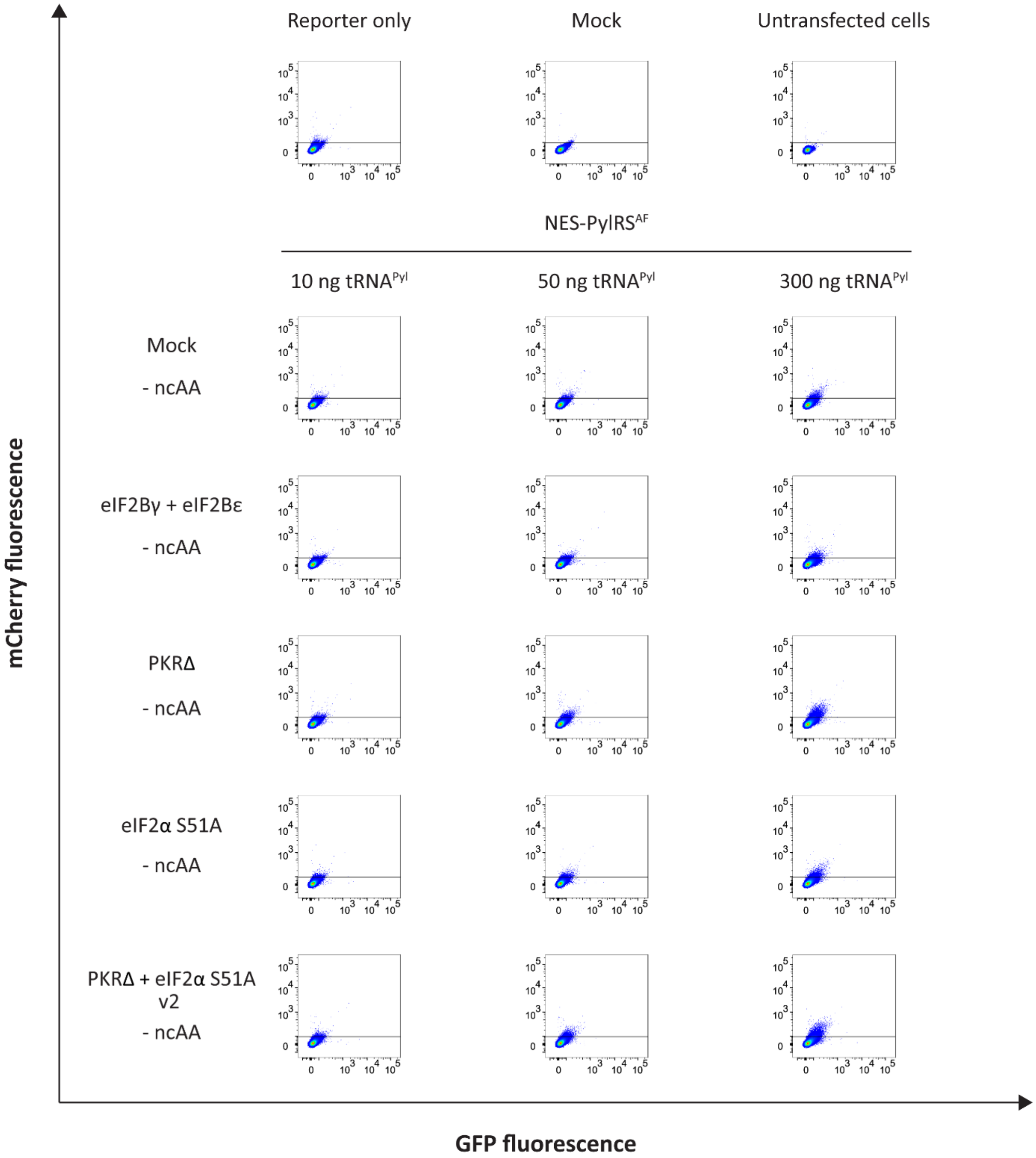

Supplementary Figure 26: FC analysis of GFP^{39TAG}, mCherry^{189TAG}-ms2 reporter showing NES-PylRS^{AF} performance in case of ncAA absence and presence of stress remodeler eIF2Bγ + eIF2Bε, PKRΔ, eIF2α S51A, or PKRΔ + eIF2α S51A v2. Concatenated data from three independent experiments are shown.

a

b

c

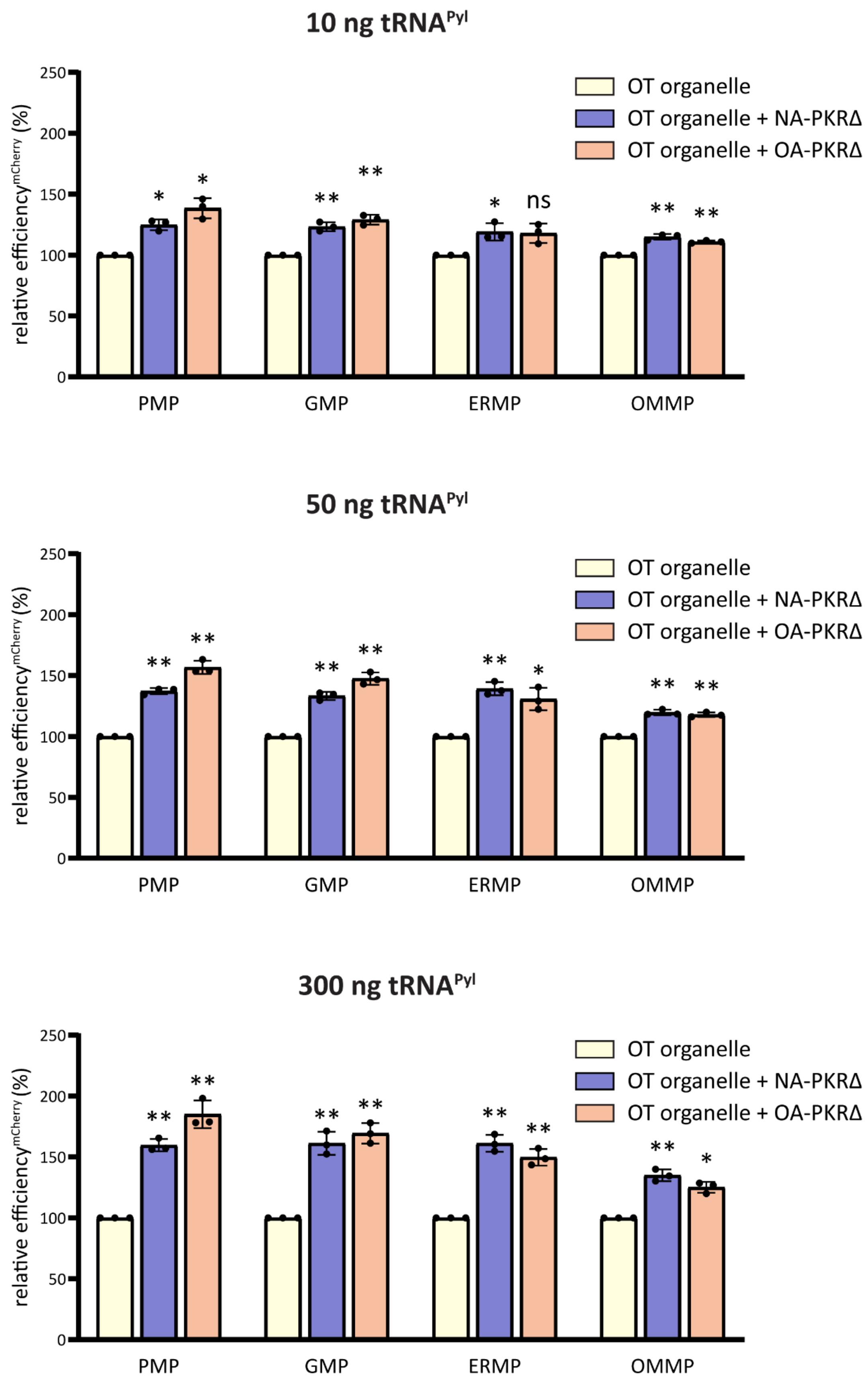

Supplementary Figure 27: Organelle-associated GCE enhancement in the presence of PKR-based stress remodelers. Bar plots show an increase in fluorescent signal in units of relative efficiency (%) in

presence of 10 ng $tRNA^{Pyl}$ (**a**), 50 ng $tRNA^{Pyl}$ (**b**), or 300 ng $tRNA^{Pyl}$ (**c**) and different OT organelles after addition of tested stress remodelers. Relative efficiency (%) is calculated as the median mCherry signal for each particular case divided by the median mCherry signal for OT organelle samples with the addition of mock plasmid. Such relative efficiency (%) calculation illustrates fold increase in organelle-associated GCE efficiency obtained after the addition of stress remodeler. Median mCherry signals were obtained after FC analysis of corresponding samples. NA denotes non-anchored, OA - organelle-anchored version of PKRΔ. Bar plots show the mean value for relative efficiencies of three independent experiments, error bars represent the SD. Ns denotes not significant (p value > 0.05), * - p value ≤ 0.05, ** - p value ≤ 0.01, p values were calculated using one-sample (two-tailed) t-test. Exact p values are provided in the Source Data file.

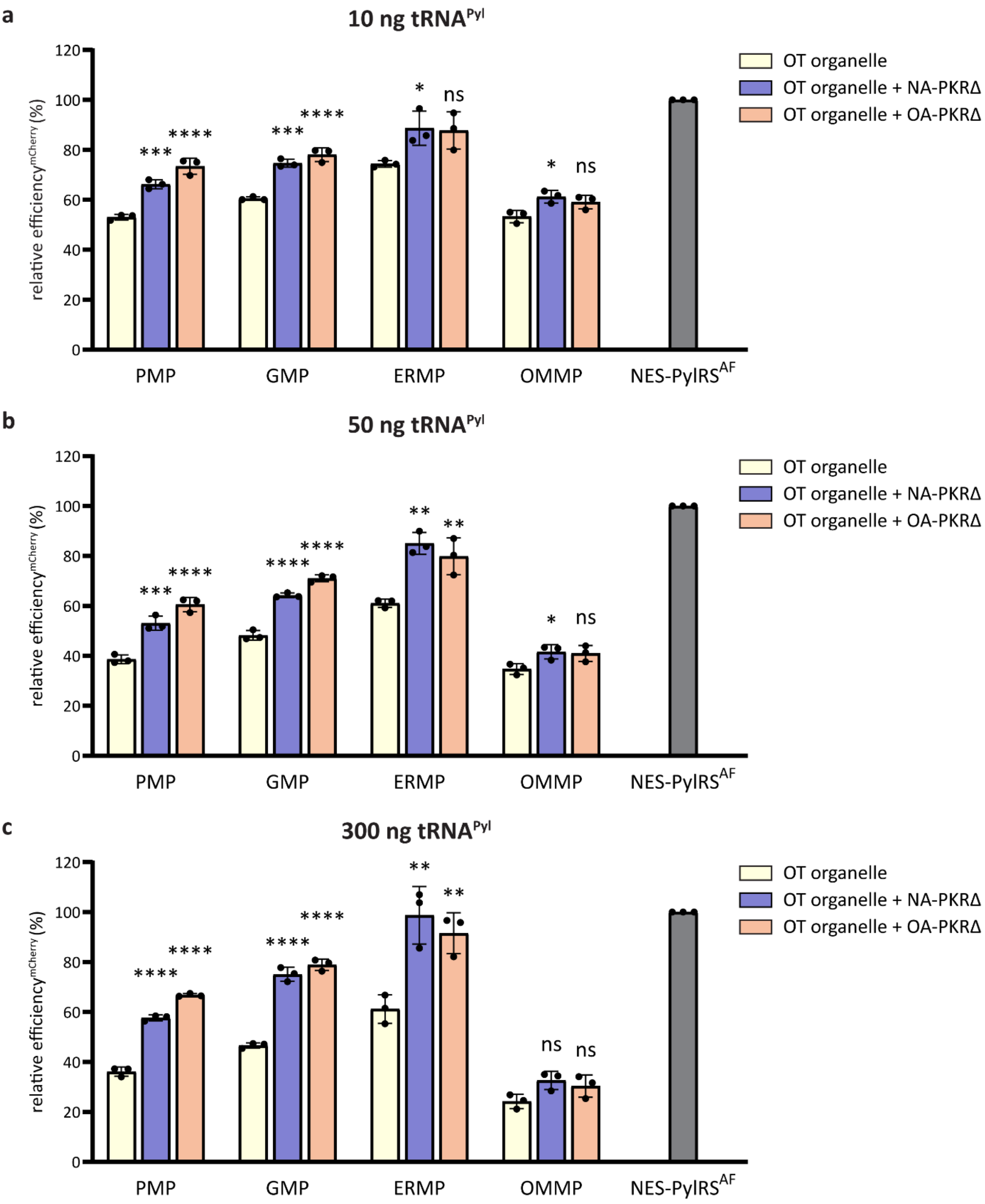

Supplementary Figure 28: Organelle-associated GCE performance in the presence of PKR-based stress remodelers normalized to cytoplasmic (NES-PylRS^{AF}) GCE level. Bar plots show an increase in fluorescent signal in units of relative efficiency (%) in presence of 10 ng tRNA^{Pyl} (**a**), 50 ng tRNA^{Pyl} (**b**), or 300 ng tRNA^{Pyl} (**c**) and different OT organelles after addition of tested stress remodelers. Relative efficiency^{mCherry} (%) is calculated as the median mCherry signal for each particular case divided by the median mCherry signal for NES-PylRS^{AF} samples with the addition of mock plasmid. Such relative efficiency^{mCherry} (%) calculation illustrates an increase in organelle-associated GCE efficiency obtained after addition of stress remodeler in comparison with common NES-PylRS^{AF} system. Median mCherry signals were obtained after FC analysis of corresponding samples. NA denotes non-anchored, OA -

organelle-anchored version of PKRΔ. Bar plots show the mean value for relative efficiencies of three independent experiments, error bars represent the SD. Ns denotes not significant (*p* value > 0.05), * - *p* value ≤ 0.05, ** - *p* value ≤ 0.01, *** - *p* value ≤ 0.001, **** - *p* value ≤ 0.0001, *p* values were calculated using one-way ANOVA with Dunnett's multiple comparison test. Exact *p* values are provided in the Source Data file.

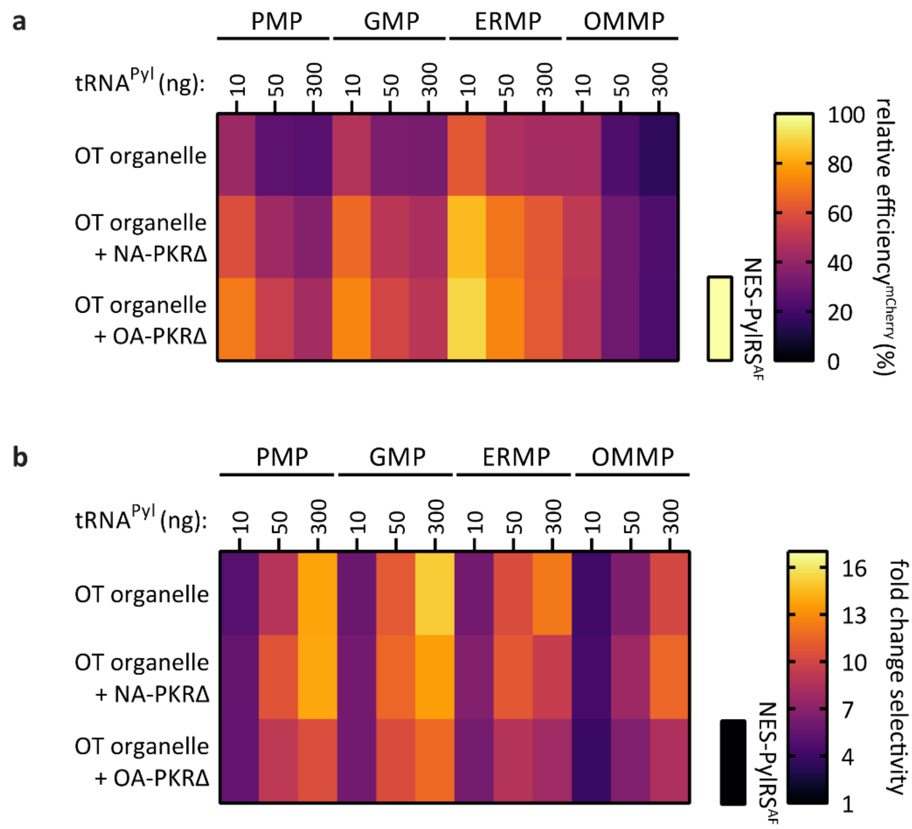

Supplementary Figure 29: Organelle-associated GCE performance in the presence of PKR-based stress remodelers. Heat maps illustrate the relative efficiency (**a**) and fold change selectivity (**b**) of distinct OT organelles in absence or presence of tested stress remodelers. Relative efficiency^{mCherry} (%) is calculated as the mean mCherry signal of a sample divided by the mean mCherry signal for cytoplasmic control (NES-PylRS^{AF}) transfected with mock plasmid. Such percentage relative efficiency^{mCherry} calculation illustrates an increase in organelle-associated GCE efficiency obtained after addition of stress remodeler in comparison with common NES-PylRS^{AF} system. Fold change selectivity was quantified as the mean mCherry signal divided by the mean GFP signal of a given system normalized to the respective ratio for the cytoplasmic control (NES-PylRS^{AF}) transfected with mock plasmid. Median mCherry and GFP signals were obtained after FC analysis of corresponding samples (Supplementary Figs. 29-32). NA denotes non-anchored, OA - organelle-anchored version of PKRΔ. Three independent experiments were performed for all samples, and the mean values for the relative efficiencies and fold change selectivity are presented.

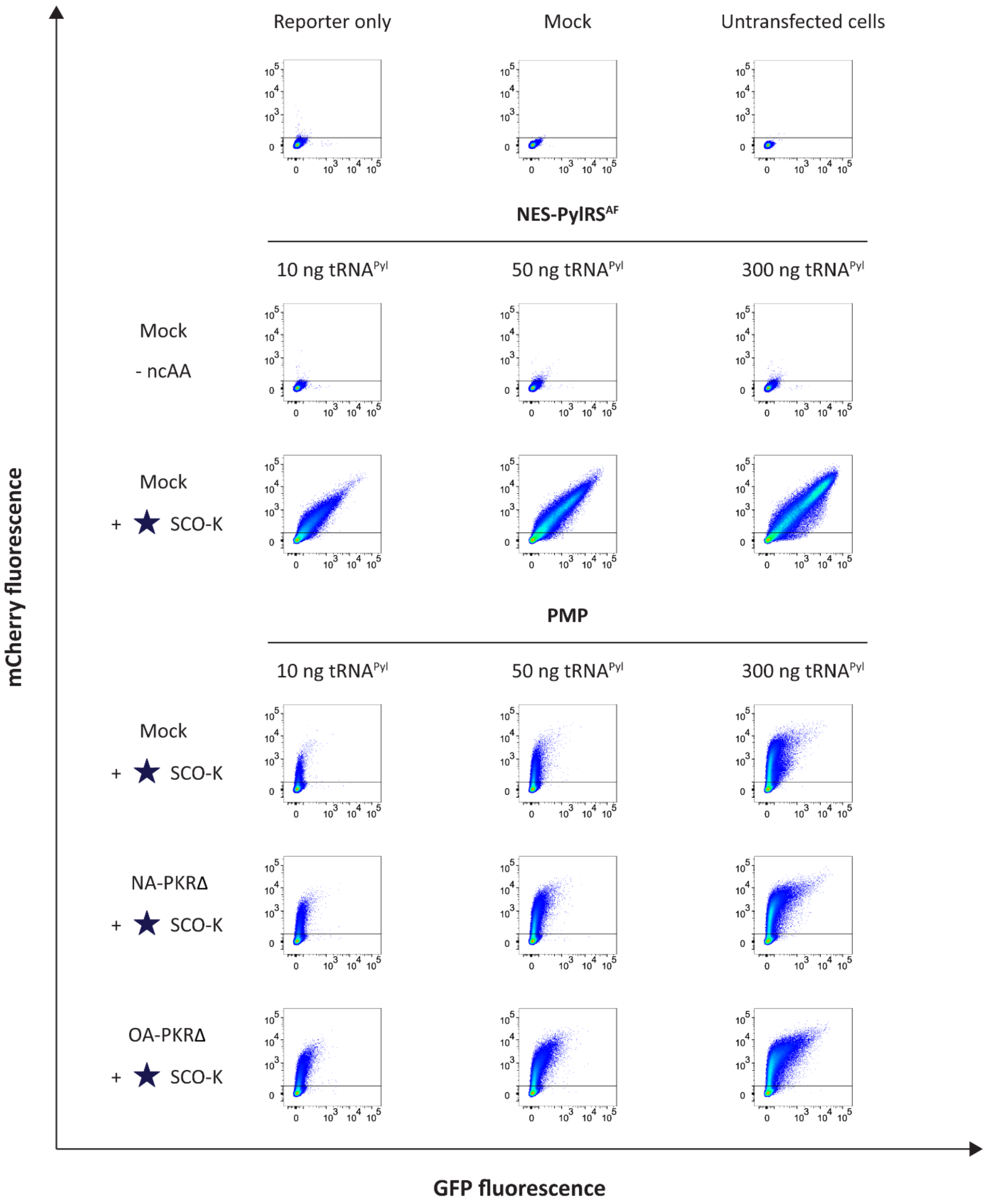

Supplementary Figure 30: FC plots showing PMP organelle performance in absence or presence of stress remodelers NA-PKRΔ or OA-PKRΔ (PMP-PKRΔ). NA denotes non-anchored, OA - organelle-anchored version of PKRΔ. Fluorescent reporter GFP^{39TAG}, $mCherry^{189TAG}$-ms2 was used in the experiments. Concatenated data from three independent experiments are shown.

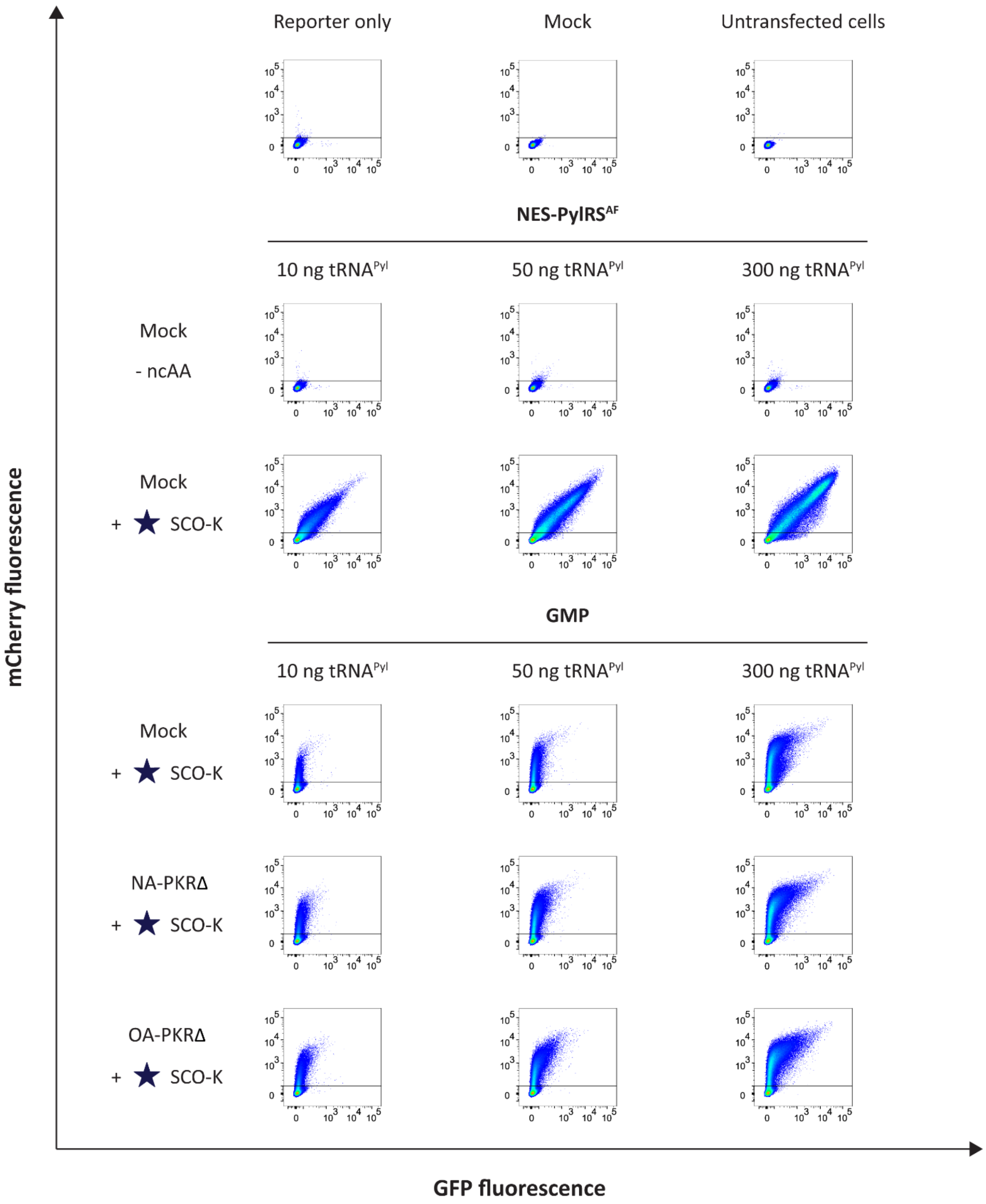

Supplementary Figure 31: FC plots showing GMP organelle performance in absence or presence of stress remodelers NA-PKRΔ or OA-PKRΔ (GMP-PKRΔ). NA denotes non-anchored, OA - organelle-anchored version of PKRΔ. Fluorescent reporter GFP^{39TAG}, $mCherry^{189TAG}$-ms2 was used in the experiments. Concatenated data from three independent experiments are shown.

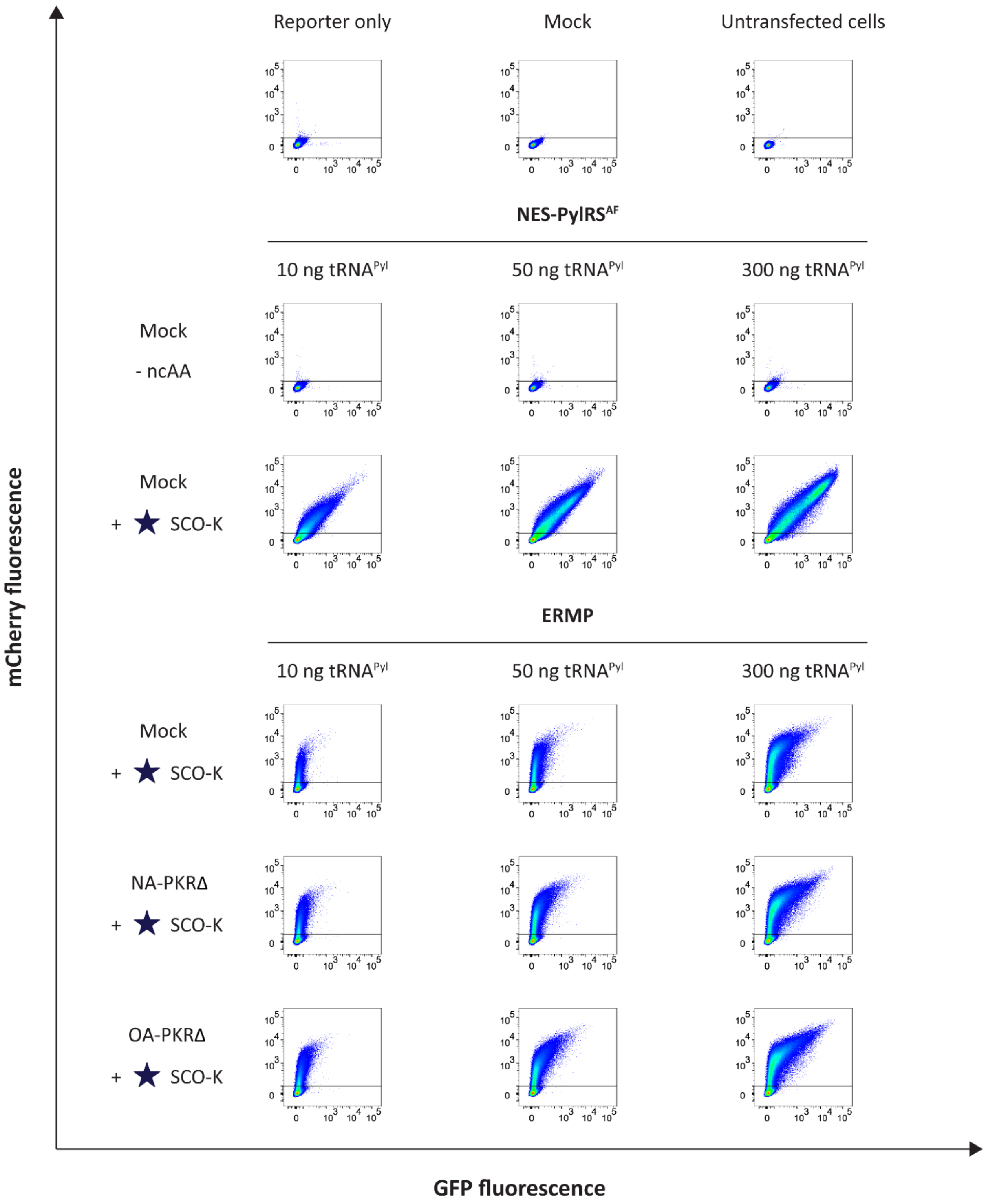

Supplementary Figure 32: FC plots showing ERMP organelle performance in absence or presence of stress remodelers NA-PKRΔ or OA-PKRΔ (ERMP-PKRΔ). NA denotes non-anchored, OA - organelle-anchored version of PKRΔ. Fluorescent reporter GFP^{39TAG}, $mCherry^{189TAG}$-ms2 was used in the experiments. Concatenated data from three independent experiments are shown.

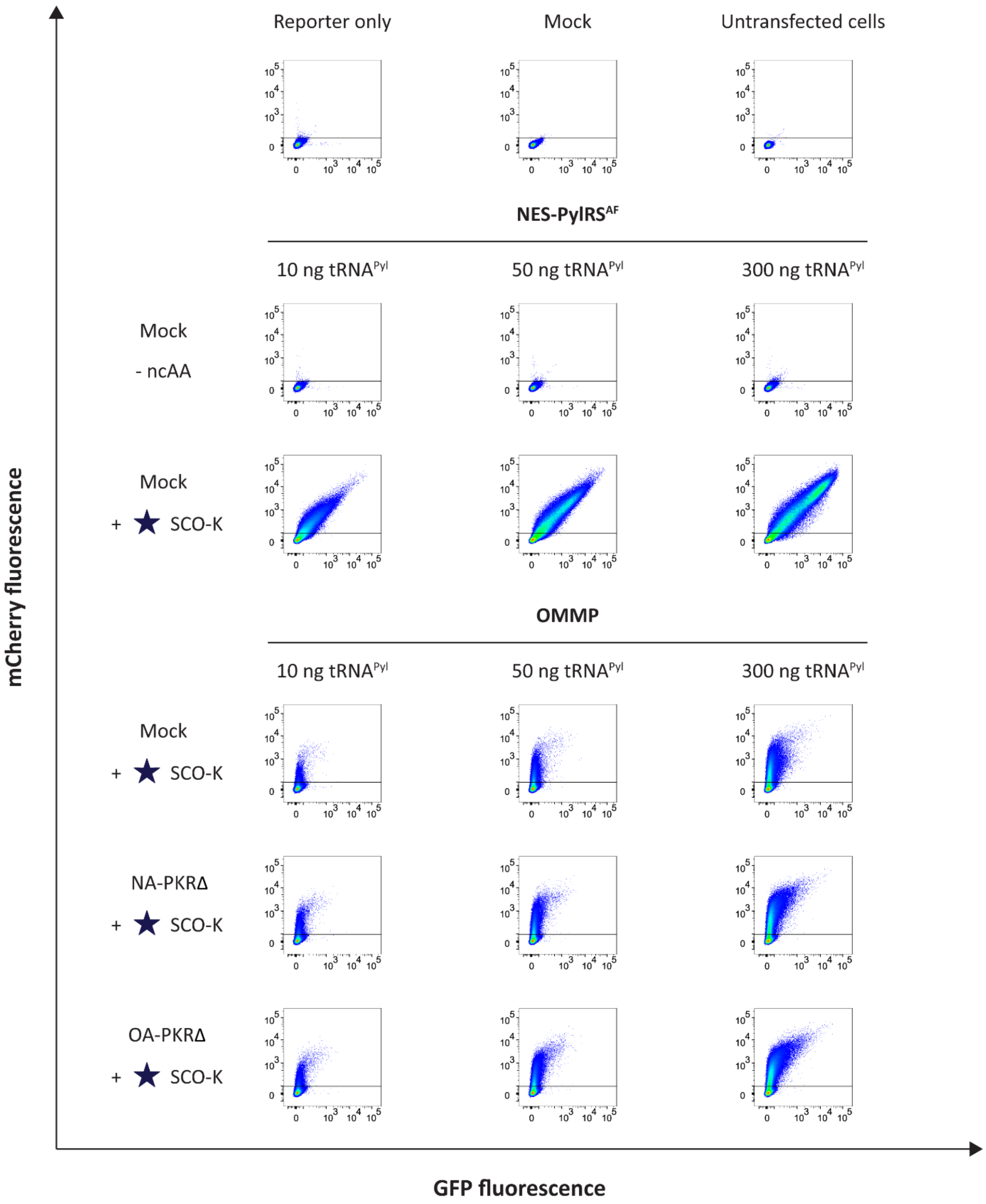

Supplementary Figure 33: FC plots showing OMMP organelle performance in absence or presence of stress remodelers NA-PKRΔ or OA-PKRΔ (OMMP-PKRΔ). NA denotes non-anchored, OA - organelle-anchored version of PKRΔ. Fluorescent reporter GFP^{39TAG}, $mCherry^{189TAG}$-ms2 was used in the experiments. Concatenated data from three independent experiments are shown.

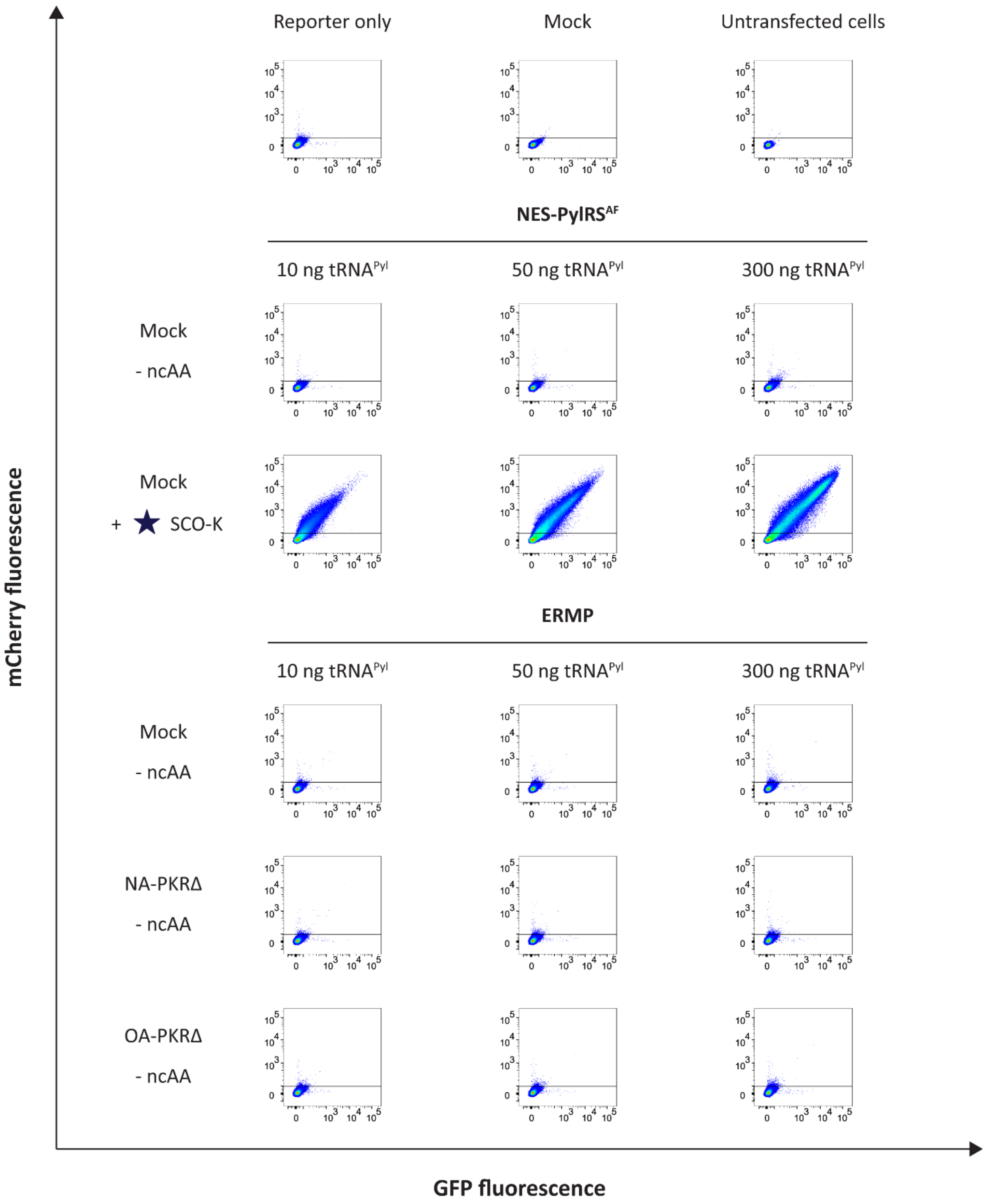

Supplementary Figure 34: FC plots showing ERMP organelle performance in case of ncAA absence and presence of stress remodelers NA-PKRΔ or OA-PKRΔ (ERMP-PKRΔ). NA denotes non-anchored, OA - organelle-anchored version of PKRΔ. Fluorescent reporter GFP^{39TAG}, $mCherry^{189TAG}$-ms2 was used in the experiments. Concatenated data from three independent experiments are shown.

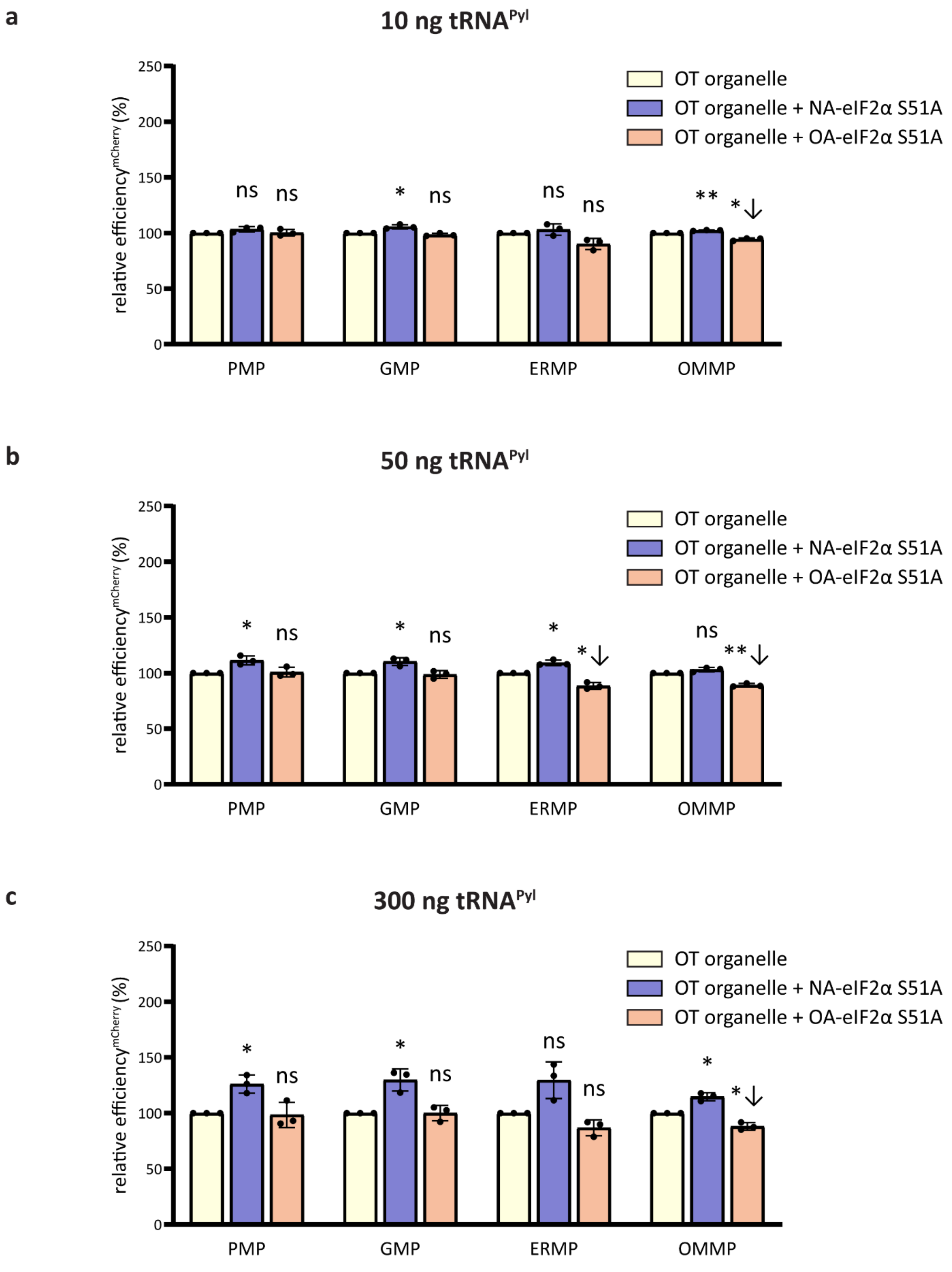
a
10 ng tRNAPyl
b
50 ng tRNAPyl
c
300 ng tRNAPyl
relative efficiencymCherry (%)
OT organelle
OT organelle + NA-eIF2α S51A
OT organelle + OA-eIF2α S51A
PMP
GMP
ERMP
OMMP
ns
*
**
0
50
100
150
200
250

Supplementary Figure 35: Organelle-associated GCE enhancement in the presence of eIF2α S51A-based stress remodelers. Bar plots show changes in fluorescent signal in units of relative efficiency (%) in presence of 10 ng $tRNA^{Pyl}$ (**a**), 50 ng $tRNA^{Pyl}$ (**b**), or 300 ng $tRNA^{Pyl}$ (**c**) and different OT organelles after addition of stress remodelers. Relative $efficiency^{mCherry}$ (%) is calculated as the median mCherry signal for each particular case divided by the median mCherry signal for OT organelle samples with the addition of mock plasmid. Such relative $efficiency^{mCherry}$ (%) calculation illustrates fold increase in organelle-associated GCE efficiency obtained after the addition of stress remodeler. Median mCherry signals were obtained after FC analysis of corresponding samples. NA denotes non-anchored, OA - organelle-anchored version of eIF2α S51A. Bar plots show the mean value for relative efficiencies of three independent experiments, error bars represent the SD. Ns denotes not significant (p value > 0.05), * - p value ≤ 0.05, ** - p value ≤ 0.01, p values were calculated using one-sample (two-tailed) t-test. *↓ and **↓ designate p value ≤ 0.05 and p value ≤ 0.01 respectively and significant decrease in relative $efficiency^{mCherry}$ (%) to compare with OT organelle with no stress remodeler addition. Exact p values are provided in the Source Data file.

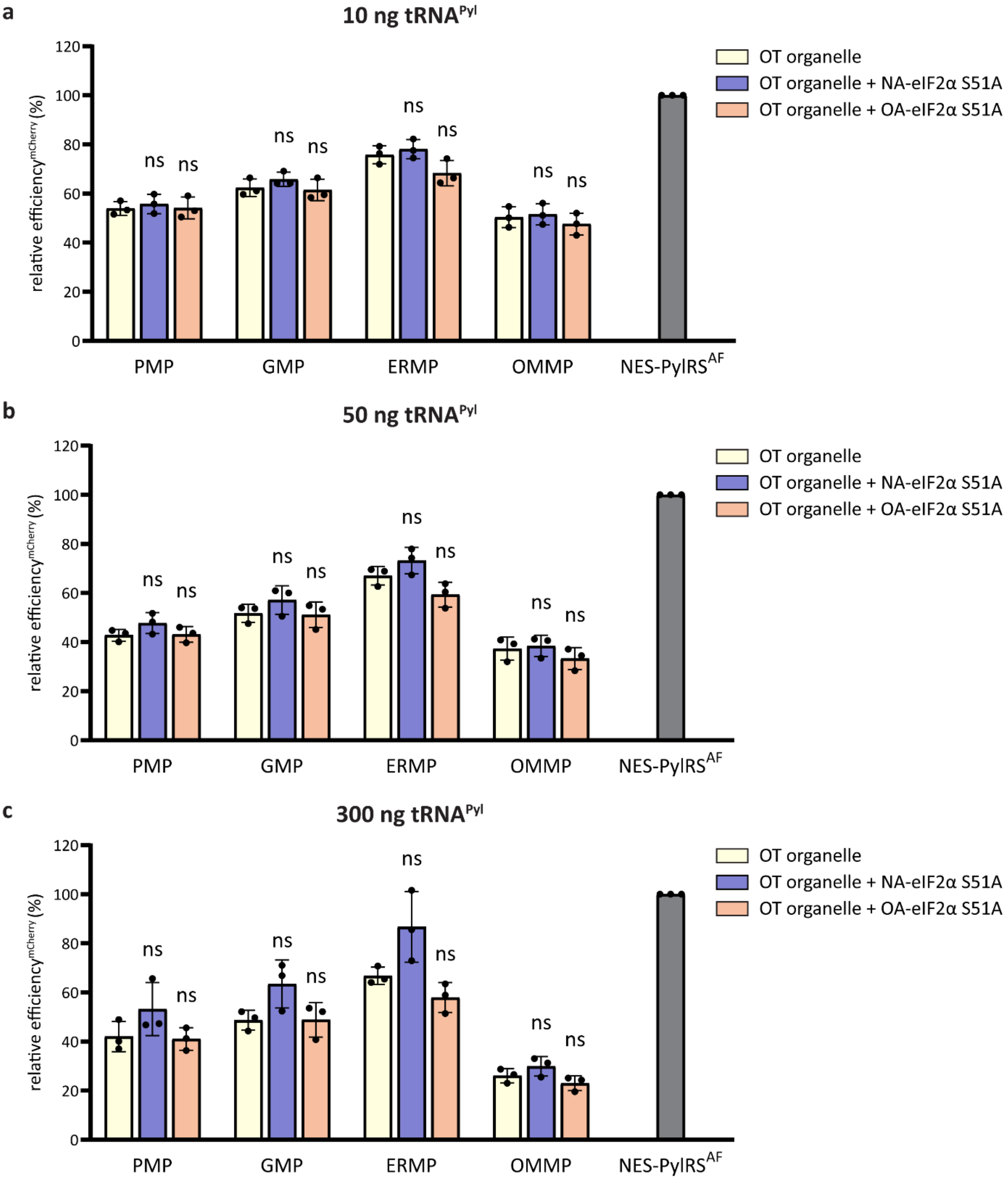

Supplementary Figure 36: Organelle-associated GCE performance in the presence of eIF2α S51A-based stress remodelers normalized to cytoplasmic (NES-PylRS^{AF}) GCE level. Bar plots show changes in fluorescent signal in units of relative efficiency (%) in presence of 10 ng tRNA^{Pyl} (**a**), 50 ng tRNA^{Pyl} (**b**), or 300 ng tRNA^{Pyl} (**c**) and different OT organelles. Relative efficiency^{mCherry} (%) is calculated as the median mCherry signal for each particular case divided by the median mCherry signal for NES-PylRS^{AF} samples with the addition of mock plasmid. Such relative efficiency^{mCherry} (%) calculation illustrates an increase in organelle-associated GCE efficiency obtained after addition of stress remodeler in comparison with common NES-PylRS^{AF} system. Median mCherry signals were obtained after FC analysis of corresponding samples. NA denotes non-anchored, OA - organelle-anchored version of

eIF2α S51A. Bar plots show the mean value for relative efficiencies of three independent experiments, error bars represent the SD. Ns denotes not significant (p value > 0.05), p values were calculated using one-way ANOVA with Dunnett's multiple comparison test. Exact p values are provided in the Source Data file.

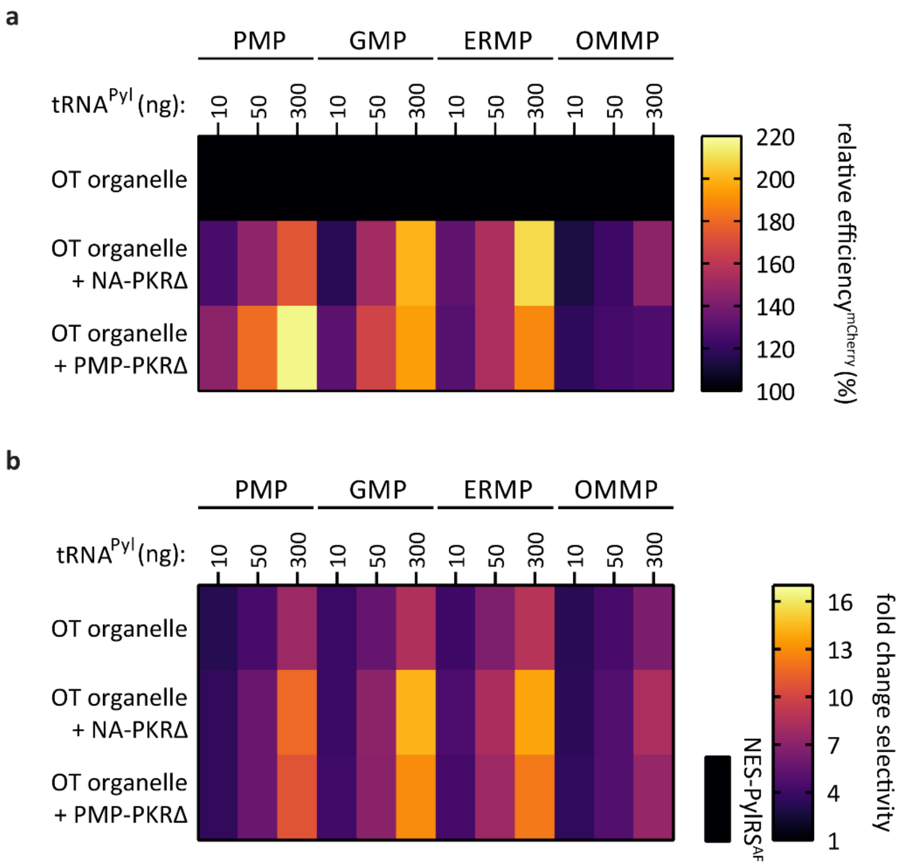

Supplementary Figure 37: PMP-PKRΔ enables enhancement of GCE realized by differently localized OT organelles. Heat maps illustrate the relative efficiency (**a**) and fold change selectivity (**b**) of distinct OT organelles in absence or presence of tested stress remodelers. Relative efficiency^{mCherry} (%) is calculated as the median mCherry signal of a sample divided by the median mCherry signal for corresponding OT organelle transfected with mock plasmid. Such relative efficiency (%) calculation illustrates fold increase in organelle-associated GCE efficiency obtained after the addition of stress remodeler. Fold change selectivity is quantified as the median mCherry signal divided by the median GFP signal of a given system normalized to the respective ratio for the cytoplasmic control (NES-PylRS^{AF}) transfected with mock plasmid. Median mCherry and GFP signals were obtained after FC analysis of corresponding samples. NA denotes non-anchored, PMP-PKRΔ – plasma membrane organelle-anchored version of PKRΔ.

a

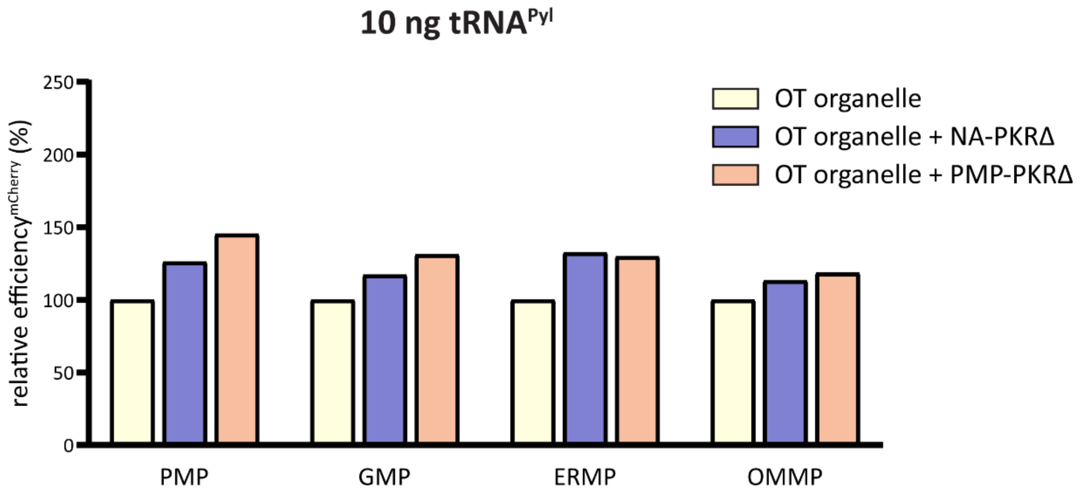

b

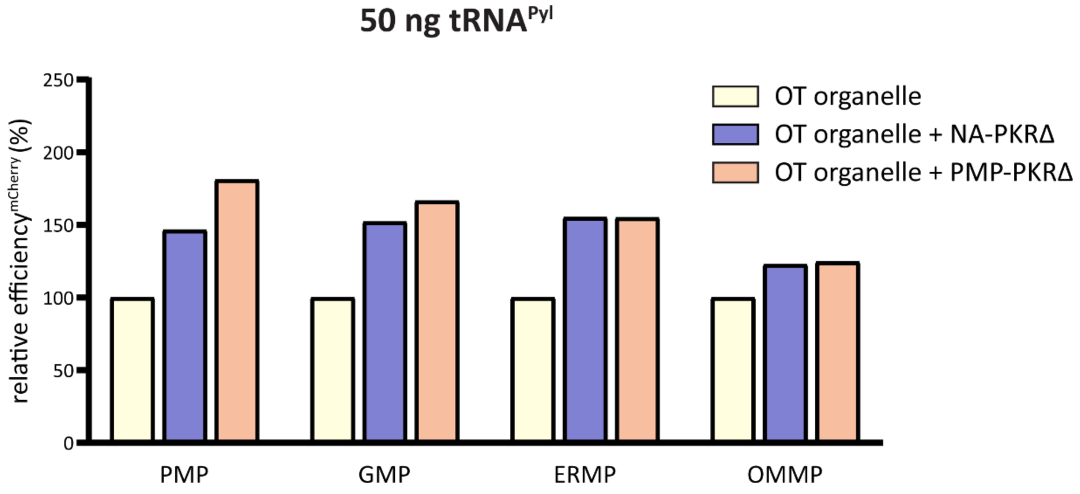

c

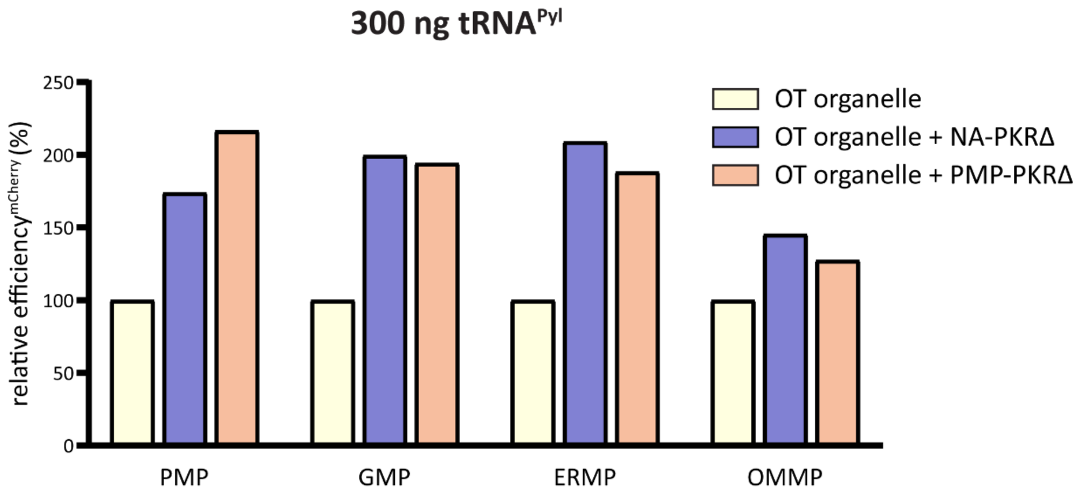

Supplementary Figure 38: PMP-PKRΔ enables enhancement of GCE realized by differently localized OT organelles. Bar plots show an increase in fluorescent signal in units of relative efficiency^{mCherry} (%) in presence of 10 ng tRNA^{Pyl} (**a**), 50 ng tRNA^{Pyl} (**b**), or 300 ng tRNA^{Pyl} (**c**) and different OT organelles after addition of tested stress remodelers. Relative efficiency^{mCherry} (%) is calculated as

the median mCherry signal for each particular case divided by the median mCherry signal for corresponding OT organelle samples with the addition of mock plasmid. Such relative efficiency (%) calculation illustrates fold increase in organelle-associated GCE efficiency obtained after addition of stress remodeler. Median mCherry signals were obtained after FC analysis of corresponding samples. NA denotes non-anchored, PMP-PKRΔ – plasma membrane organelle-anchored version of PKRΔ.

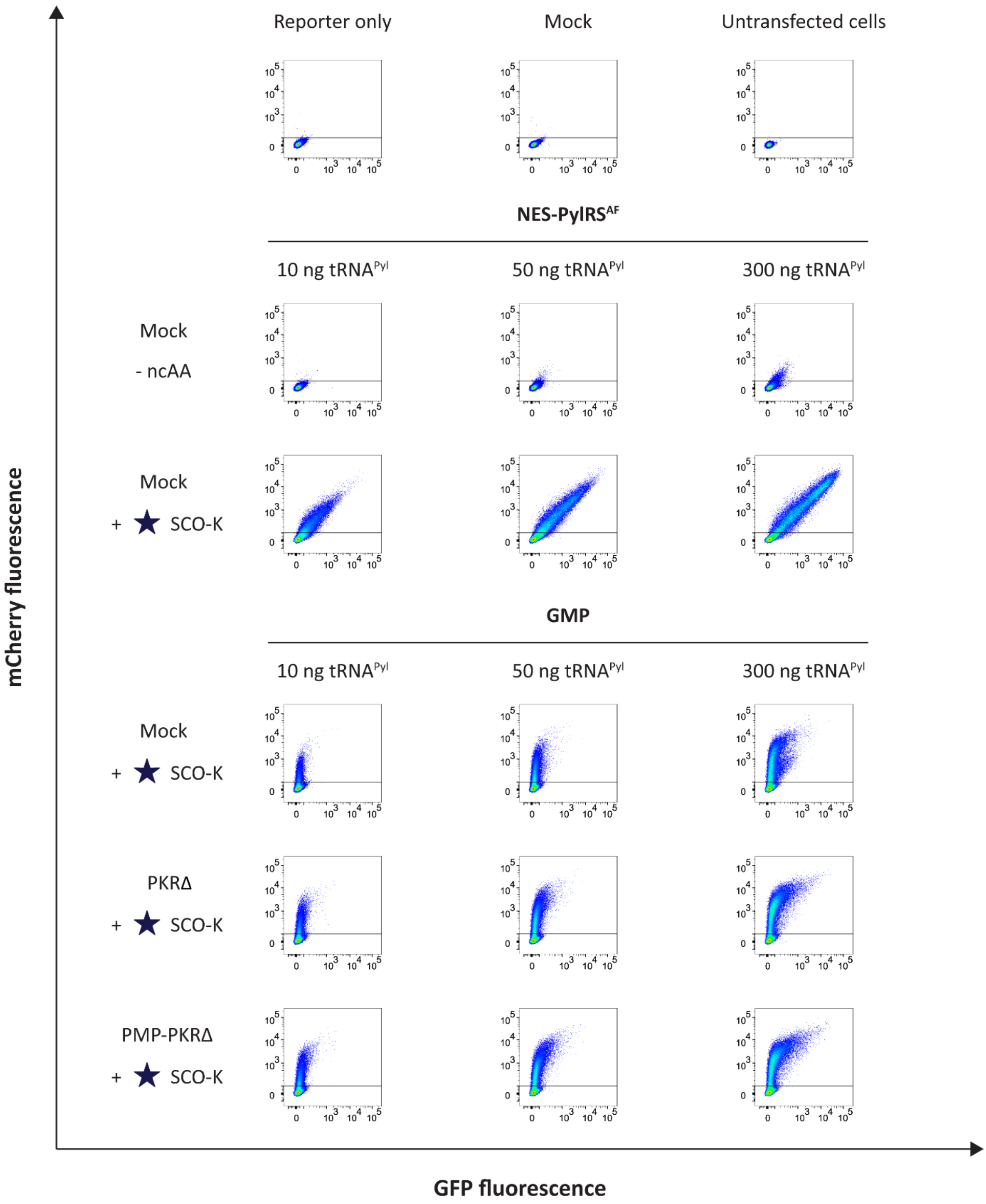

Supplementary Figure 39: FC plots showing GMP organelle performance in absence or presence of stress remodelers NA-PKRΔ or PMP-PKRΔ. NA denotes non-anchored, PMP-PKRΔ – plasma membrane organelle-anchored version of PKRΔ. Fluorescent reporter GFP^{39TAG}, $mCherry^{189TAG}$-ms2 was used in the experiment. FC plots for one performed biological replicate are shown.

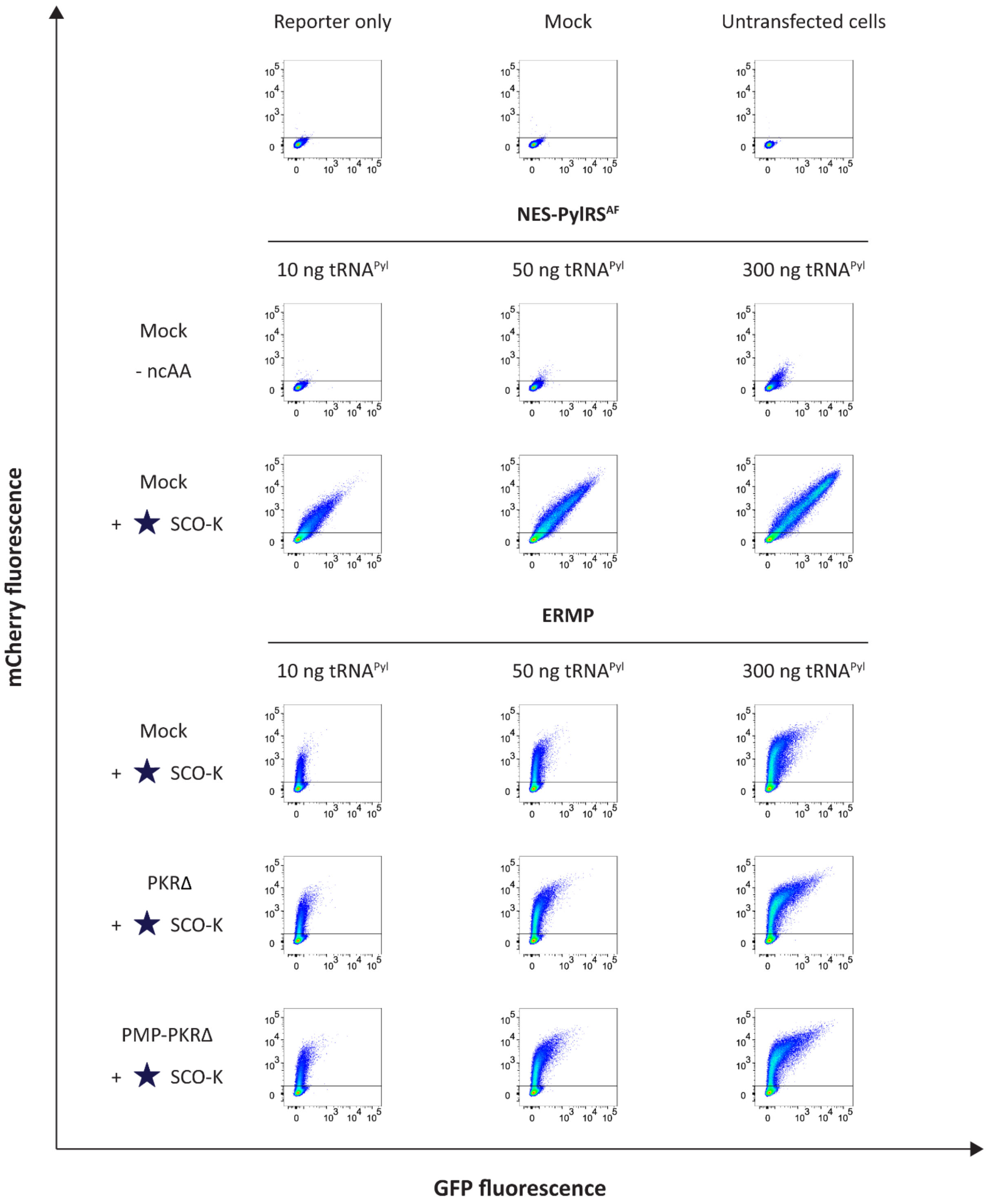

Supplementary Figure 40: FC plots showing ERMP organelle performance in absence or presence of stress remodelers NA-PKRΔ or PMP-PKRΔ. NA denotes non-anchored, PMP-PKRΔ – plasma membrane organelle-anchored version of PKRΔ. Fluorescent reporter GFP^{39TAG}, $mCherry^{189TAG}$-ms2 was used in the experiment. FC plots for one performed biological replicate are shown.

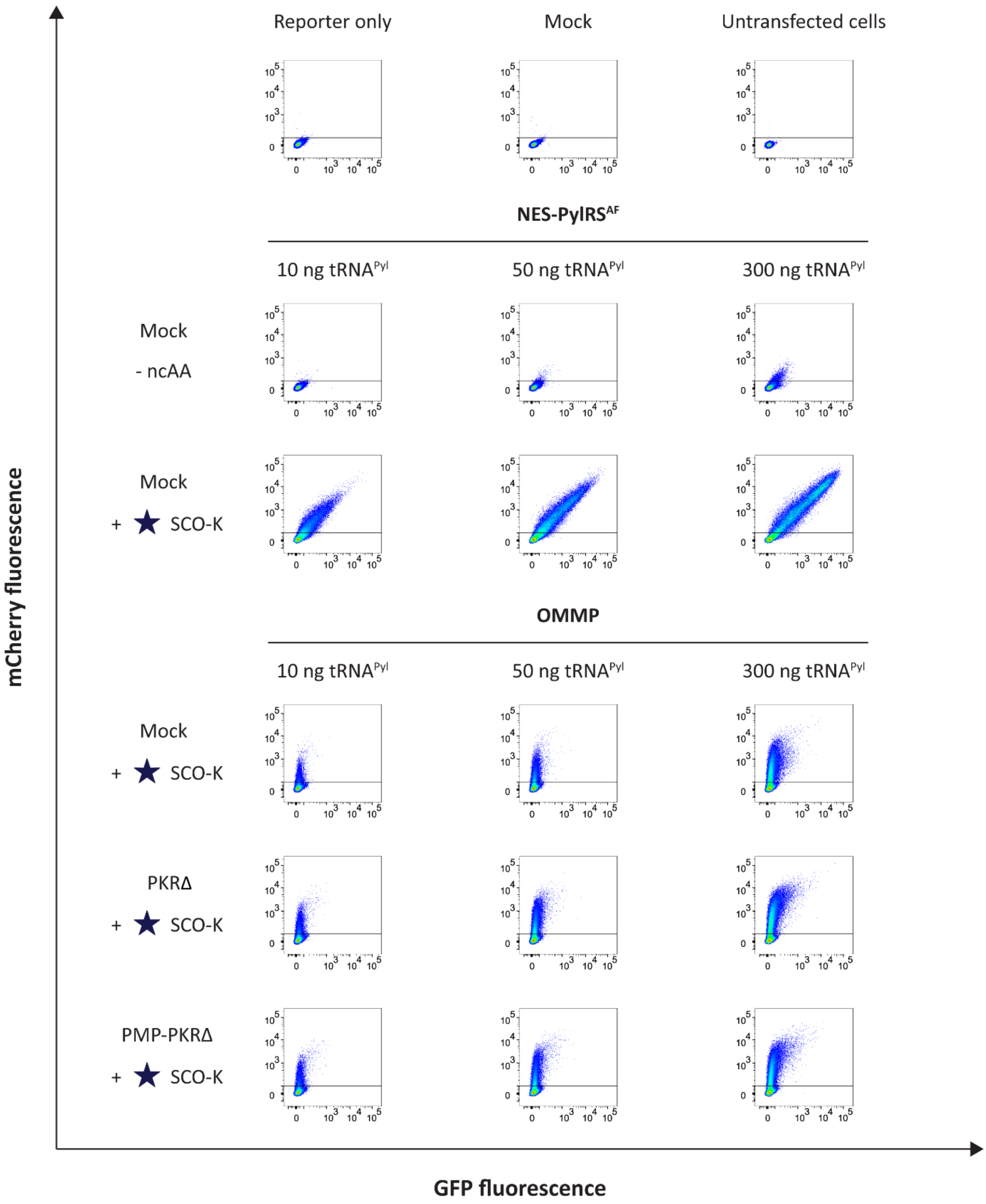

Supplementary Figure 41: FC plots showing OMMP organelle performance in absence or presence of stress remodelers NA-PKRΔ or PMP-PKRΔ. NA denotes non-anchored, PMP-PKRΔ – plasma membrane organelle-anchored version of PKRΔ. Fluorescent reporter GFP^{39TAG}, $mCherry^{189TAG}$-ms2 was used in the experiment. FC plots for one performed biological replicate are shown.

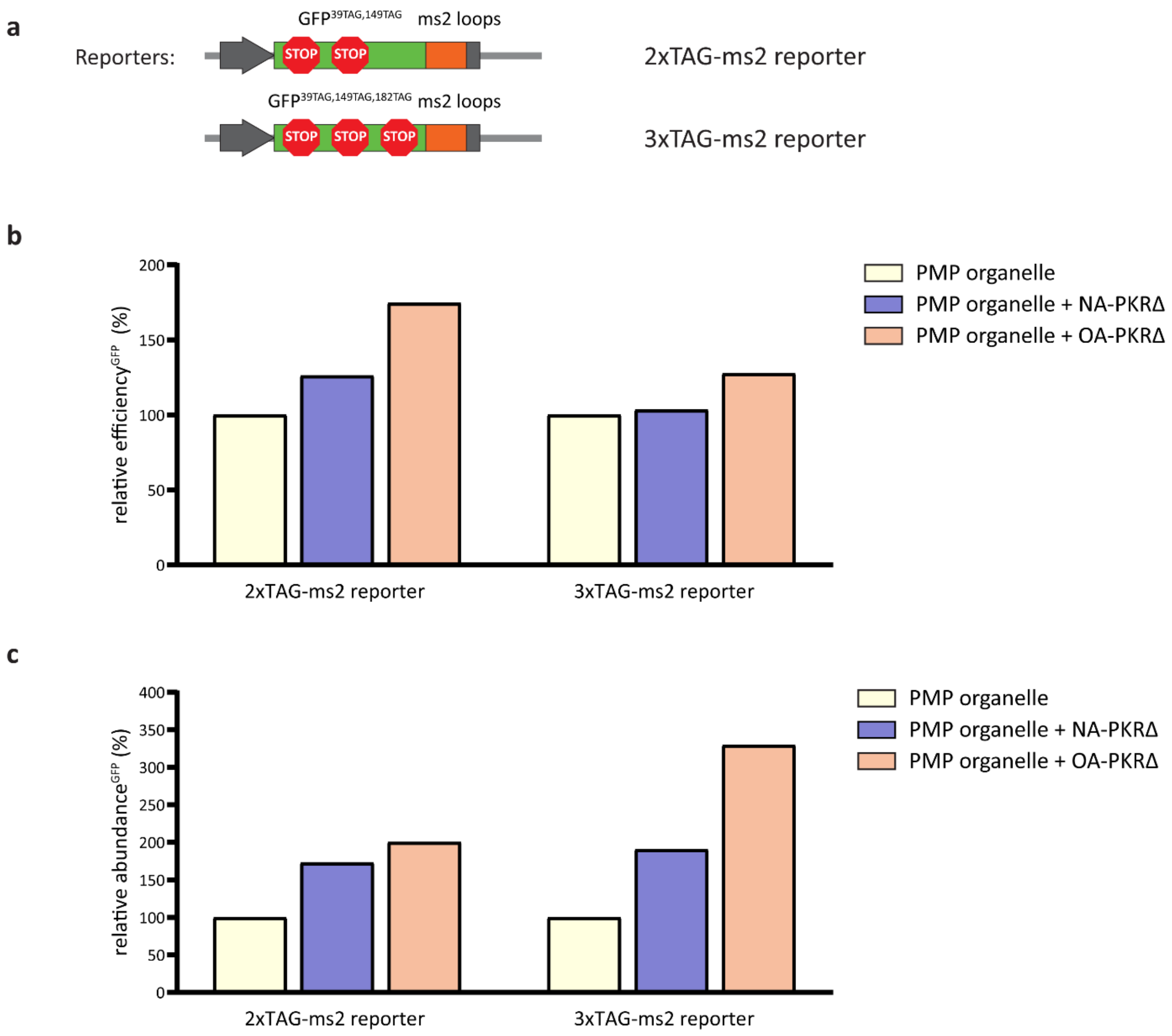

Supplementary Figure 42: PKR-based stress remodelers enhance multiple ncAA incorporation performed by PMP organelle. a, Schematic representation of GFP^{39TAG,149TAG}-ms2 (2xTAG-ms2) and GFP^{39TAG,149TAG,182TAG}-ms2 (3xTAG-ms2) reporters. Bar plots show changes in fluorescent signal in units of relative efficiency^{GFP} (%) (**b**) and changes in relative abundance^{GFP} (%) (**c**) for tested PMP organelle after addition of tested stress remodelers. Relative efficiency^{mCherry} (%) is calculated as the median mCherry signal for each particular case divided by the median mCherry signal for OT (PMP) organelle samples with the addition of mock plasmid. Relative abundance^{GFP} (%) is quantified as percentage of GFP-positive cells in a sample divided by percentage of GFP-positive cells in the corresponding PMP organelle sample transfected with mock plasmid. Median GFP signals were obtained after FC analysis of corresponding samples. NA denotes non-anchored, OA – organelle-anchored version of PKRΔ (in this experiment PMP-PKRΔ was used).

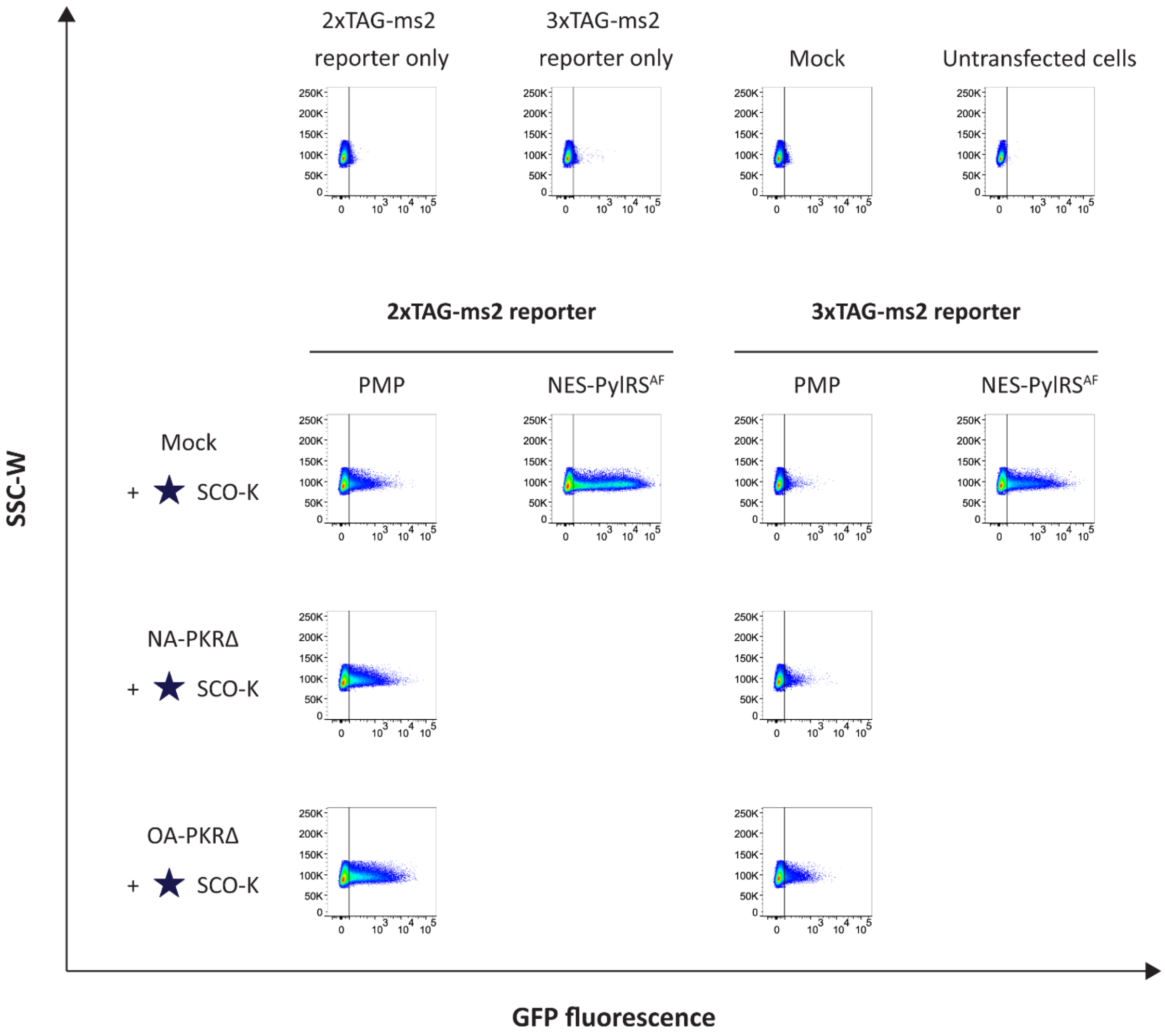

Supplementary Figure 43: FC plots showing PMP organelle performance for multiple ncAA incorporation in absence or presence of stress remodelers NA-PKRΔ or OA-PKRΔ (PMP-PKRΔ). NA denotes non-anchored, OA - organelle-anchored version of PKRΔ (in this experiment PMP-PKRΔ was used). Samples were tested with fluorescent reporters GFP^{39TAG,149TAG}-ms2 (2xTAG-ms2) and GFP^{39TAG,149TAG,182TAG}-ms2 (3xTAG-ms2). FC plots for one performed biological replicate are shown.

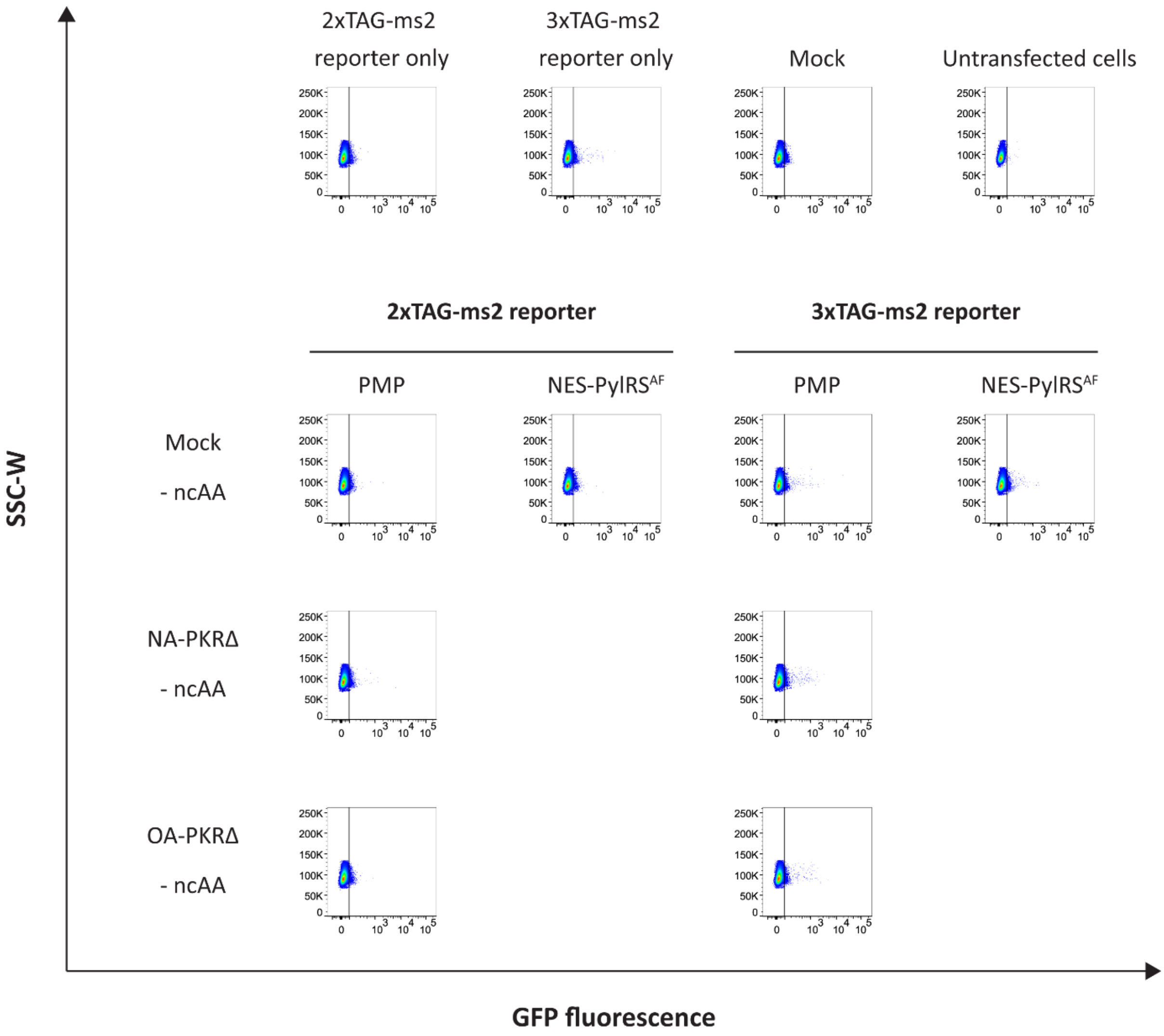

Supplementary Figure 44: FC plots showing PMP organelle performance for multiple ncAA incorporation in case of ncAA absence and presence of stress remodelers NA-PKRΔ or OA-PKRΔ (PMP-PKRΔ). NA denotes non-anchored, OA - organelle-anchored version of PKRΔ (in this experiment PMP-PKRΔ was used). Samples were tested with fluorescent reporters GFP^{39TAG,149TAG}-ms2 (2xTAG-ms2) and GFP^{39TAG,149TAG,182TAG}-ms2 (3xTAG-ms2). FC plots for one performed biological replicate are shown.

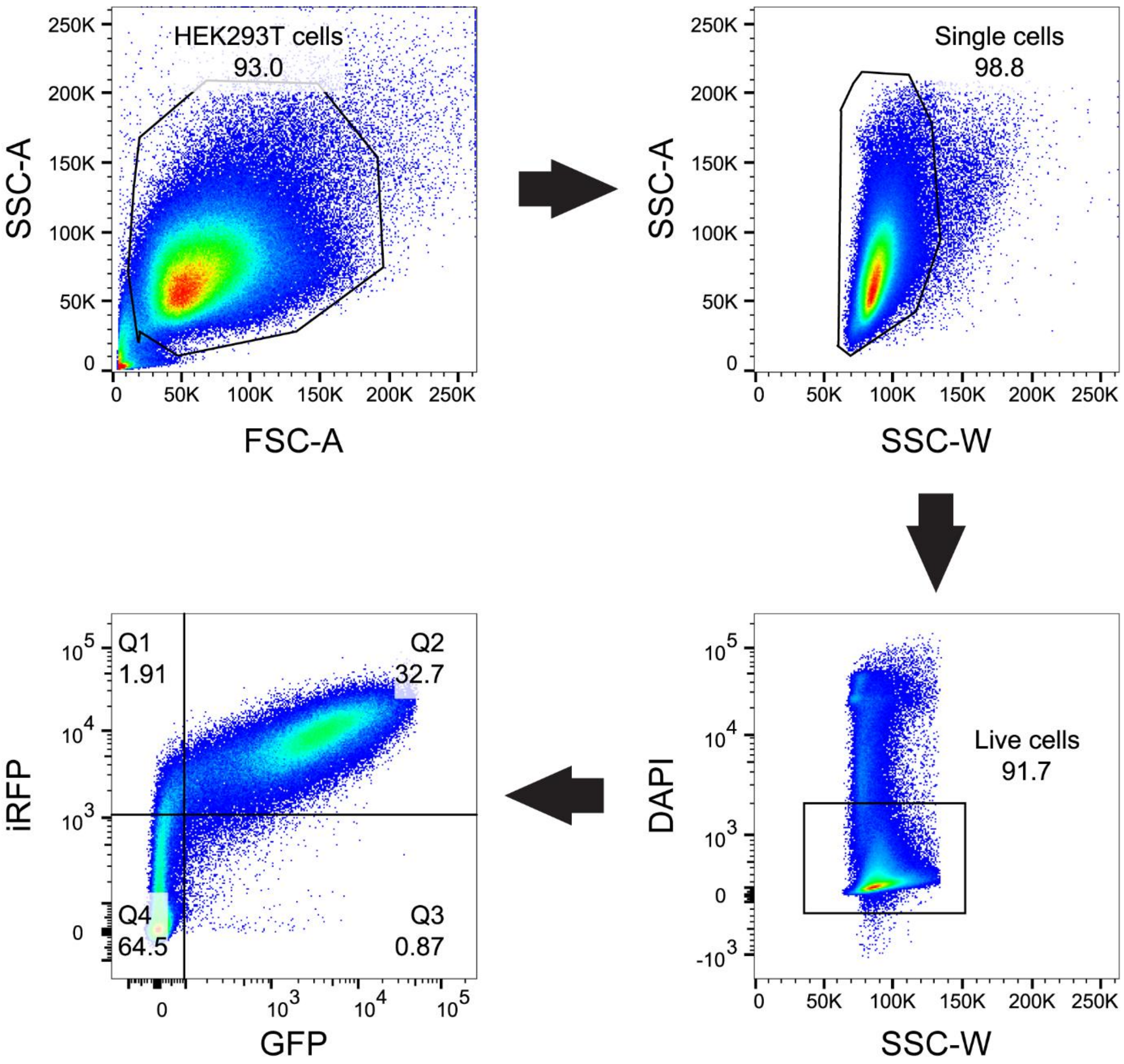

Supplementary Figure 45: Flow cytometry gating strategy for all experiments with iRFP-GFP^{39TAG}, iRFP-GFP^{39TAG,149TAG} and iRFP-GFP^{39TAG,149TAG,182TAG} reporter. Representative example of using iRFP-GFP^{39TAG} reporter transfected with NES-PylRS^{AF}, 300 ng tRNA^{Pyl}, and mock plasmid is demonstrated. See details in Methods section.

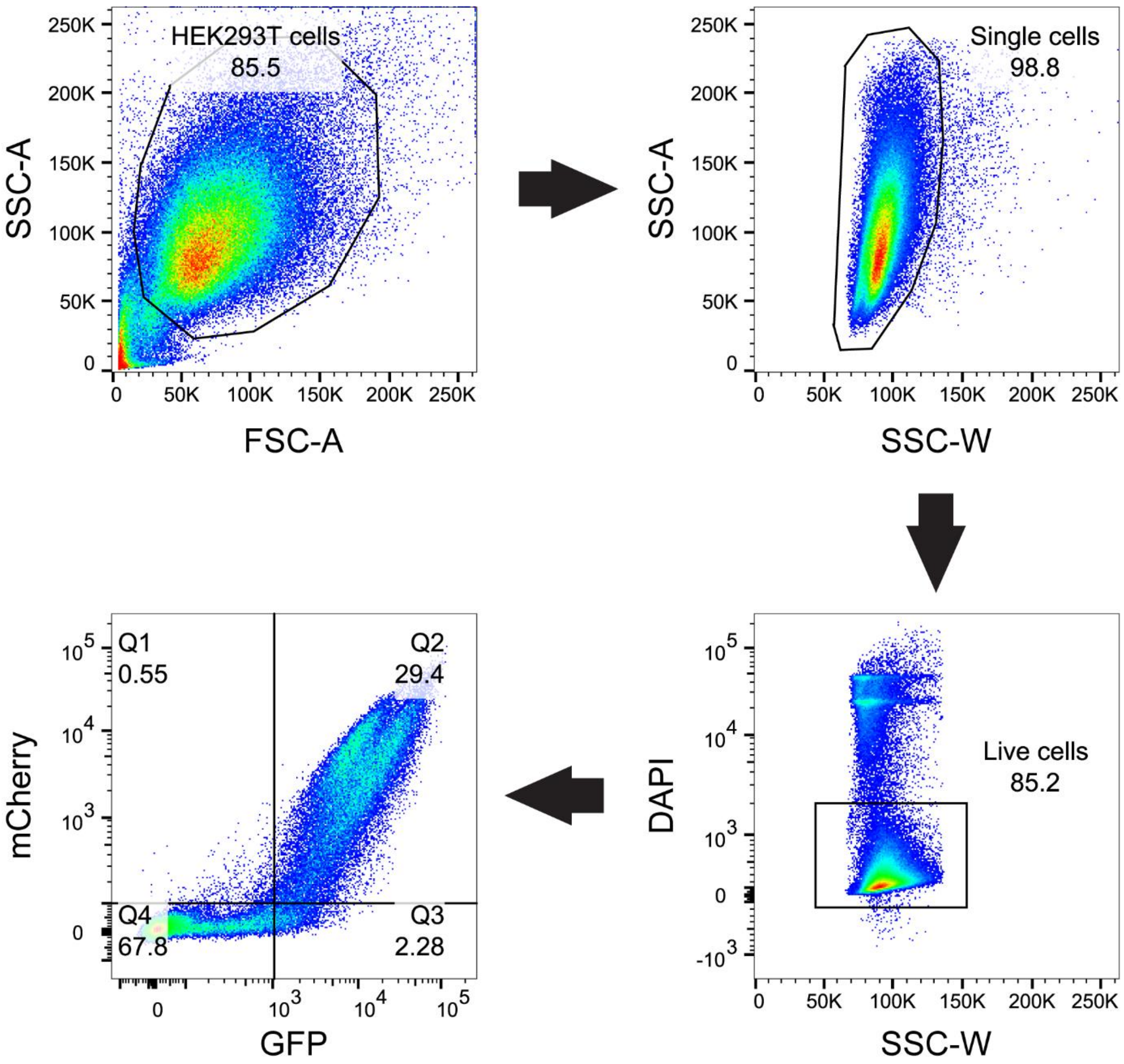

Supplementary Figure 46: Flow cytometry gating strategy for all experiments with GFP-P2A-T2A-mCherry^{189TAG} reporter. Representative example of using NES-PylRS^{AF} transfected with 300 ng tRNA^{Pyl} and mock plasmid is demonstrated. See details in Methods section.

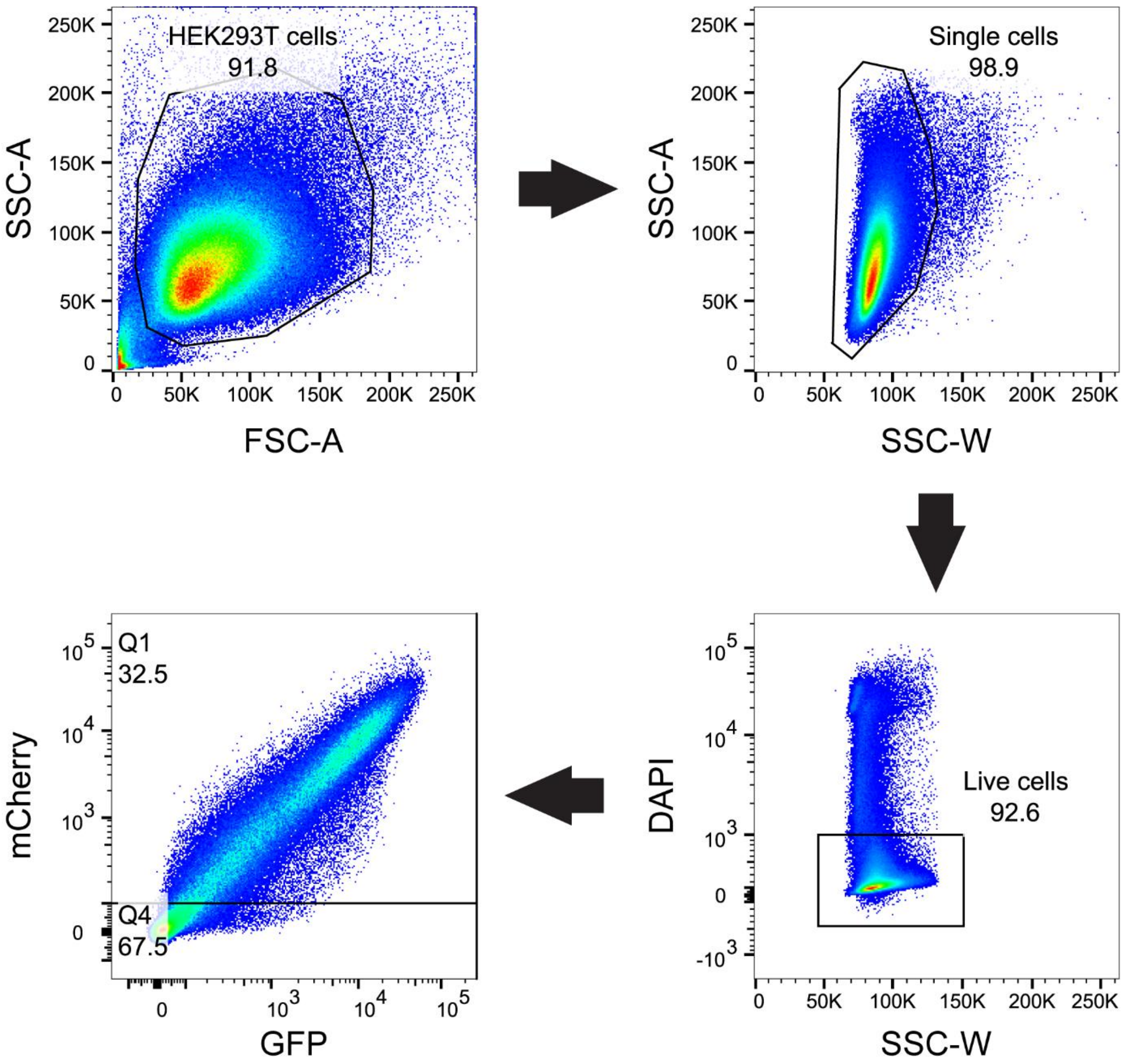

Supplementary Figure 47: Flow cytometry gating strategy for all experiments with GFP^{39TAG}, mCherry^{189TAG}-ms2 reporter. Representative example of using NES-PylRS^{AF} transfected with 300 ng tRNA^{Pyl} and mock plasmid is demonstrated. See details in Methods section.

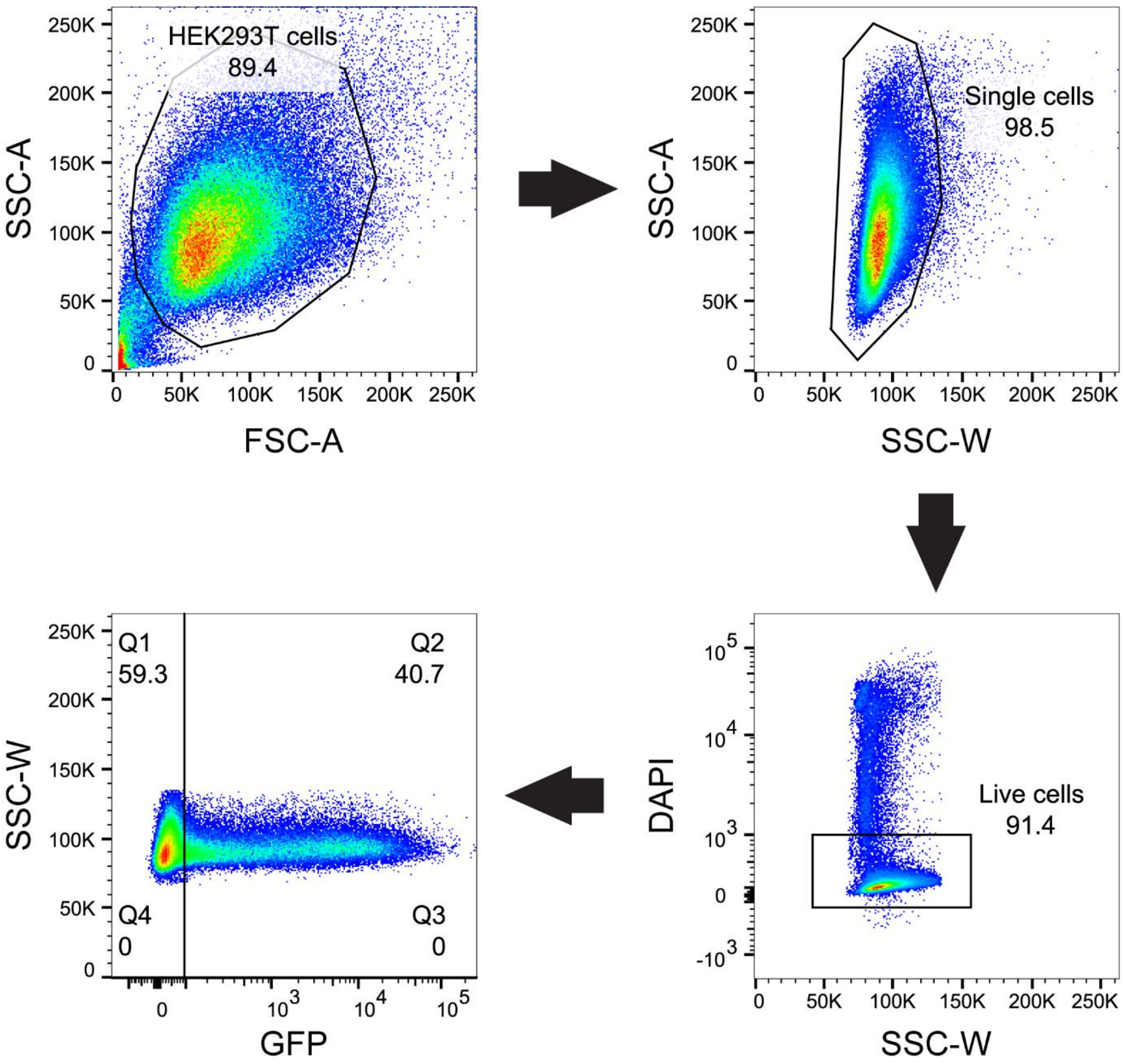

Supplementary Figure 48: Flow cytometry gating strategy for all experiments with GFP^{39TAG,149TAG}-ms2 and GFP^{39TAG,149TAG,182TAG}-ms2 reporter. Representative example of using GFP^{39TAG,149TAG}-ms2 reporter transfected with NES-PylRS^{AF}, 300 ng tRNA^{Pyl}, and mock plasmid is demonstrated. See details in Methods section.

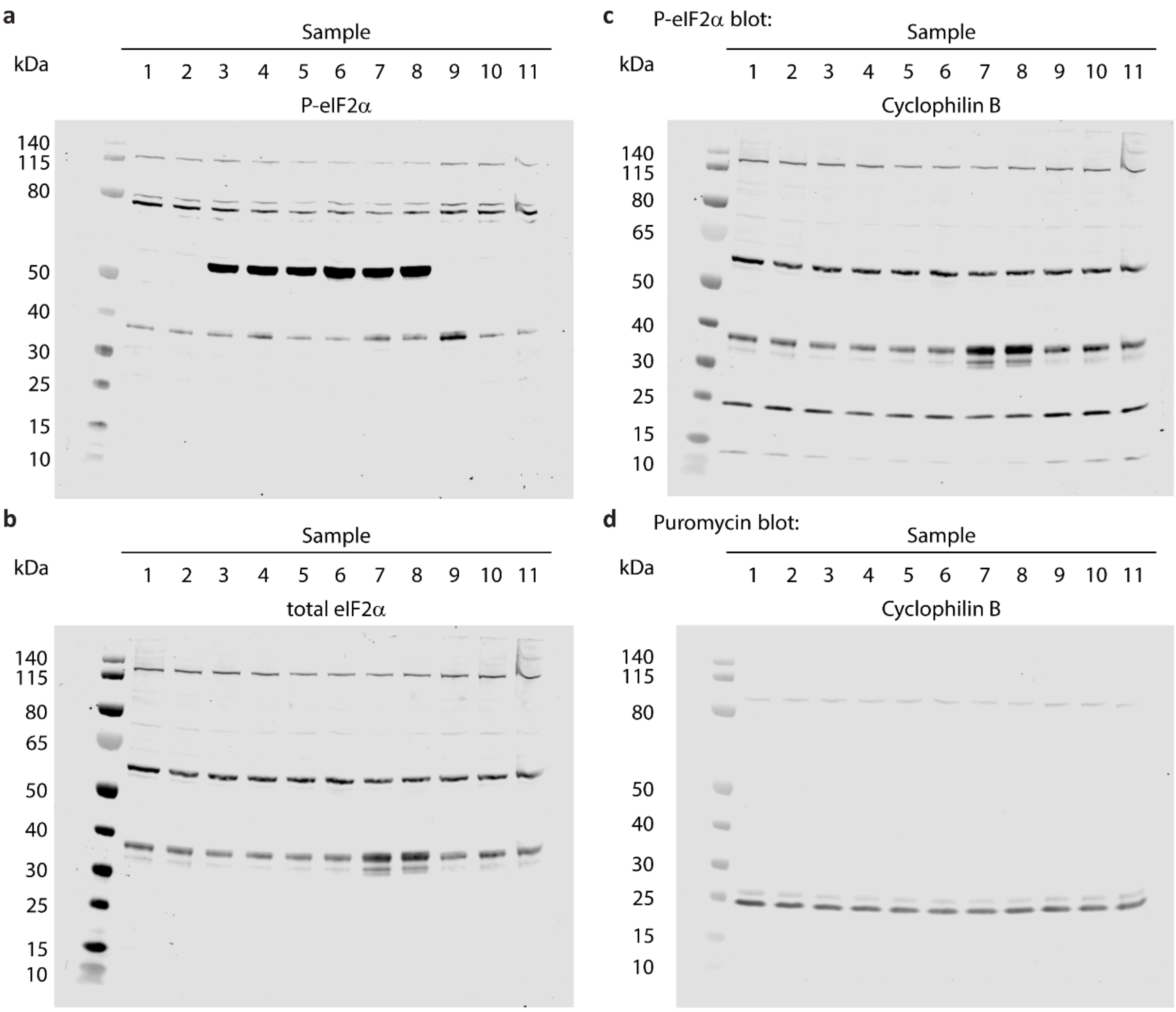

Supplementary Figure 49: Full scans of Western blots presented in Supplementary Fig. 16. **a**, Scan of Western blot showing results for probing the membrane for the P-eIF2α level assessment with rabbit anti-phospho-eIF2α antibody. **b**, Scan of Western blot showing results for probing the membrane for the P-eIF2α level assessment with mouse anti-total eIF2α antibody. **c**, Scan of Western blot showing results for probing the membrane for the P-eIF2α level assessment with mouse anti-cyclophilin B antibody. **d**, Scan of Western blot showing results for probing the membrane for the puromycin incorporation assay with rabbit anti-cyclophilin B antibody. All sample numbers can be found in the caption to Supplementary Fig. 16.

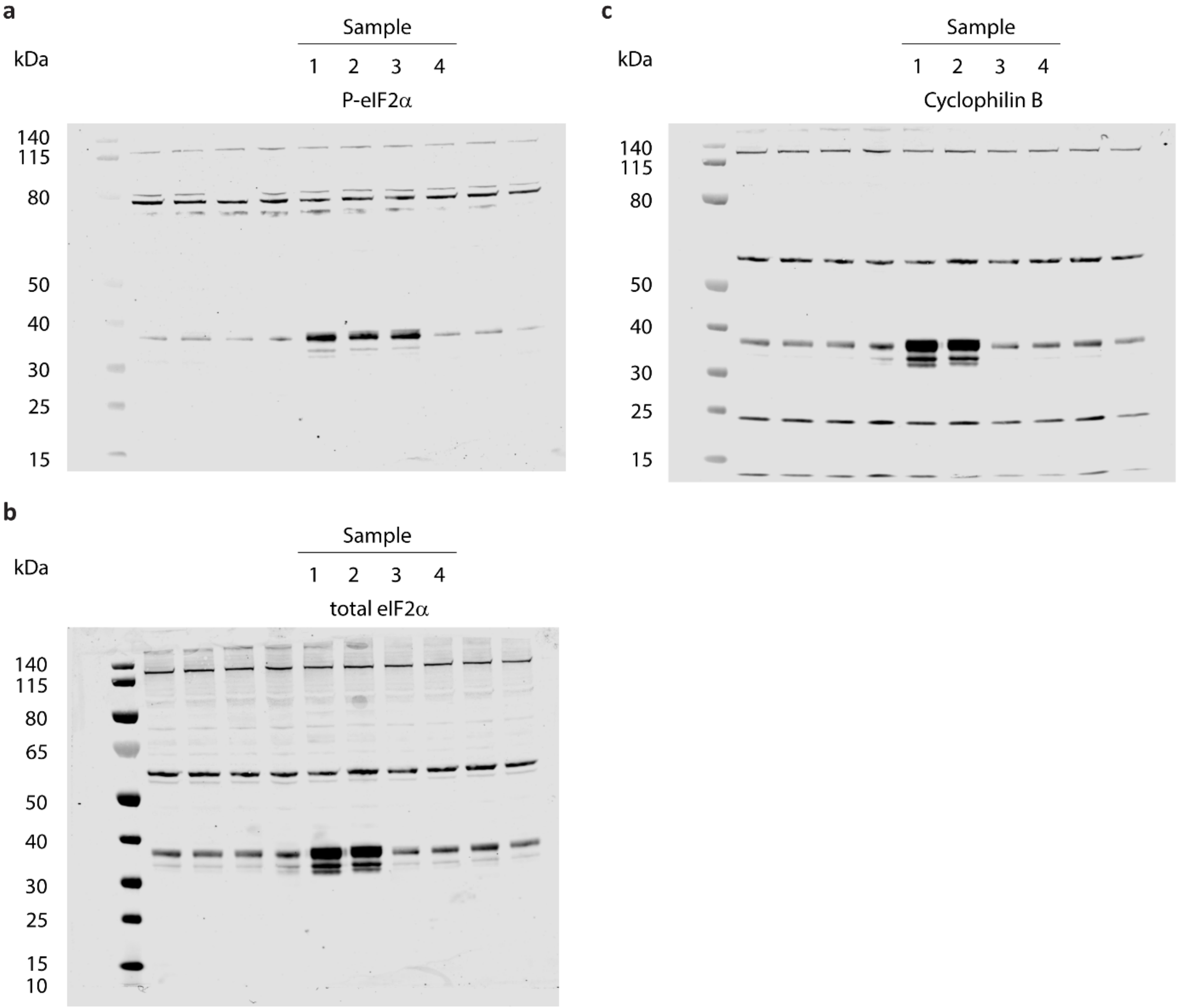

Supplementary Figure 50: Full scans of Western blots presented in Supplementary Fig. 17. **a**, Scan of Western blot showing results for probing the membrane for the P-eIF2α level assessment with rabbit anti-phospho-eIF2α antibody. **b**, Scan of Western blot showing results for probing the membrane for the P-eIF2α level assessment with mouse anti-total eIF2α antibody. **c**, Scan of Western blot showing results for probing the membrane for the P-eIF2α level assessment with mouse anti-cyclophilin B antibody. All sample numbers can be found in the caption to Supplementary Fig. 17.

Supplementary Table 1: Plasmids used in the present study.

**Common plasmids**		
Name		*Plasmid backbone* (*promoter*)
NES-PylRS^{AF}		pcDNA3.1 (CMV)
tRNA^{Pyl}		pUC57 (U6)
NES-PylRS^{AF} + tRNA^{Pyl}		pcDNA3.1 (CMV, U6)
iRFP-GFP^{39TAG}		pCI (CMV)
iRFP-GFP^{39TAG,149TAG}		pCI (CMV)
iRFP-GFP^{39TAG,149TAG,182TAG}		pCI (CMV)
GFP-P2A-T2A-mCherry^{189TAG}		pCI (CMV)
GFP^{39TAG}, mCherry^{189TAG}-ms2		pBI (CMV2, CMV1)
GFP^{39TAG,149TAG}-ms2		pBI (CMV1)
GFP^{39TAG,149TAG,182TAG}-ms2		pBI (CMV1)
trastuzumab LC		pcDNA3.4 (CMV)
trastuzumab HC		pcDNA3.4 (CMV)
trastuzumab HC^{121TAG}		pcDNA3.4 (CMV)
Mock		pcDNA3.1
eIF2Bγ		pBI (CMV1)
eIF2Bε		pBI (CMV2)
eIF2Bγ + eIF2Bε		pBI (CMV1, CMV2)
PKRΔ		pcDNA3.1 (CMV)
PKRΔ K64E		pcDNA3.1 (CMV)
eIF2α WT		pcDNA3.1 (CMV)
eIF2α S51A		pcDNA3.1 (CMV)
(+Gly) eIF2α WT		pcDNA3.1 (CMV)
(+Gly) eIF2α S51A		pcDNA3.1 (CMV)
PKRΔ + eIF2α S51A v1		pBI (CMV1, CMV2)
PKRΔ + eIF2α S51A v2		pcDNA3.1 (CMV)
PKRΔ + eIF2α S51A v3		pcDNA3.1 (CMV)
**Organelle constructs**		
Name	*Protein order in fusions*	*Plasmid backbone* (*promoter*)
PMP	LCK$_{1-10}$-FUS-MCP-NES-PylRS^{AF}	pcDNA3.1 (CMV)
GMP	EBAG9$_{1-29}$-FUS-MCP-NES-PylRS^{AF}	pcDNA3.1 (CMV)
ERMP	CYPIIC1$_{1-27}$-FUS-MCP-NES-PylRS^{AF}	pcDNA3.1 (CMV)
OMMP	TOM20$_{1-70}$-FUS-MCP-NES-PylRS^{AF}	pcDNA3.1 (CMV)
OA(PMP)-PKRΔ	LCK$_{1-10}$-FUS-PKRΔ	pcDNA3.1 (CMV)
OA(GMP)-PKRΔ	EBAG9$_{1-29}$-FUS-PKRΔ	pcDNA3.1 (CMV)
OA(ERMP)-PKRΔ	CYPIIC1$_{1-27}$-FUS-PKRΔ	pcDNA3.1 (CMV)
OA(OMMP)-PKRΔ	TOM20$_{1-70}$-FUS-PKRΔ	pcDNA3.1 (CMV)
OA(PMP)-eIF2α S51A	LCK$_{1-10}$-FUS-eIF2α S51A	pcDNA3.1 (CMV)

OA(GMP)-eIF2α S51A	$EBAG9_{1-29}$-FUS-eIF2α S51A	pcDNA3.1 (CMV)
OA(ERMP)-eIF2α S51A	$CYPIIC1_{1-27}$-FUS-eIF2α S51A	pcDNA3.1 (CMV)
OA(OMMP)-eIF2α S51A	$TOM20_{1-70}$-FUS-eIF2α S51A	pcDNA3.1 (CMV)

Supplementary References:

1. Kozak, M. An analysis of 5'-noncoding sequences from 699 vertebrate messenger RNAs. *Nucleic Acids Res.* **15**, 8125–8148 (1987).
2. Rabouw, H. H. *et al.* Small molecule ISRIB suppresses the integrated stress response within a defined window of activation. *Proc. Natl. Acad. Sci. U.S.A.* **116**, 2097–2102 (2019).
3. Cagnetta, R. *et al.* Noncanonical modulation of the eIF2 pathway controls an increase in local translation during neural wiring. *Mol. Cell* **73**, 474-489.e5 (2019).
4. Zyryanova, A. F. *et al.* ISRIB blunts the integrated stress response by allosterically antagonising the inhibitory effect of phosphorylated eIF2 on eIF2B. *Mol. Cell* **81**, 88-103.e6 (2021).
5. Schmidt, E. K., Clavarino, G., Ceppi, M. & Pierre, P. SUnSET, a nonradioactive method to monitor protein synthesis. *Nat Methods* **6**, 275–277 (2009).
