# Supplementary material for: Remodeling the cellular stress response for enhanced genetic code expansion in mammalian cells
Source: Nat Commun. 2023 Oct 31;14:6931. doi: 10.1038/s41467-023-42689-2 (PMC10616097; doi:10.1038/s41467-023-42689-2)
Supplement: Supplementary file 3 — Description of Additional Supplementary Files Document [file 41467_2023_42689_MOESM3_ESM.pdf]

<u>Description of Additional Supplementary Files</u>

Supplementary Data 1 - Protein sequences of stress remodelers
