# Supplementary material for: Remodeling the cellular stress response for enhanced genetic code expansion in mammalian cells
Source: Nat Commun. 2023 Oct 31;14:6931. doi: 10.1038/s41467-023-42689-2 (PMC10616097; doi:10.1038/s41467-023-42689-2)
Supplement: Supplementary file 5 — Reporting Summary [file 41467_2023_42689_MOESM5_ESM.pdf]

nature portfolio

Corresponding author(s): Edward A. Lemke

Last updated by author(s): Sep 24, 2023

Reporting Summary

Nature Portfolio wishes to improve the reproducibility of the work that we publish. This form provides structure for consistency and transparency in reporting. For further information on Nature Portfolio policies, see our Editorial Policies and the Editorial Policy Checklist.

Statistics

For all statistical analyses, confirm that the following items are present in the figure legend, table legend, main text, or Methods section.

n/a	Confirmed	
☐	☒	The exact sample size (*n*) for each experimental group/condition, given as a discrete number and unit of measurement
☐	☒	A statement on whether measurements were taken from distinct samples or whether the same sample was measured repeatedly
☐	☒	The statistical test(s) used AND whether they are one- or two-sided<br>*Only common tests should be described solely by name; describe more complex techniques in the Methods section.*
☒	☐	A description of all covariates tested
☐	☒	A description of any assumptions or corrections, such as tests of normality and adjustment for multiple comparisons
☐	☒	A full description of the statistical parameters including central tendency (e.g. means) or other basic estimates (e.g. regression coefficient) AND variation (e.g. standard deviation) or associated estimates of uncertainty (e.g. confidence intervals)
☐	☒	For null hypothesis testing, the test statistic (e.g. *F*, *t*, *r*) with confidence intervals, effect sizes, degrees of freedom and *P* value noted<br>*Give P values as exact values whenever suitable.*
☒	☐	For Bayesian analysis, information on the choice of priors and Markov chain Monte Carlo settings
☒	☐	For hierarchical and complex designs, identification of the appropriate level for tests and full reporting of outcomes
☒	☐	Estimates of effect sizes (e.g. Cohen's *d*, Pearson's *r*), indicating how they were calculated

Our web collection on statistics for biologists contains articles on many of the points above.

Software and code

Policy information about availability of computer code

Data collection	FACS Diva version 9.0.1 - flow cytometry; Tecan i-control 2.0.10.0 - enzyme-linked immunosorbent assay (ELISA); LI-COR Acquisition Software 1.1.0.61 - Western blot
Data analysis	FlowJo version 10.7.1 (BD Biosciences) - flow cytometry; RStudio 2022.02.1+461 - statistical analysis; MyAssays (https://www.myassays.com/, 5PL curve) - ELISA; Image Studio Lite 5.2 - Western blot; Microsoft Excel 2016, GraphPad Prism 9.1.1 (225) - for all experiments

For manuscripts utilizing custom algorithms or software that are central to the research but not yet described in published literature, software must be made available to editors and reviewers. We strongly encourage code deposition in a community repository (e.g. GitHub). See the Nature Portfolio guidelines for submitting code & software for further information.

Data

Policy information about availability of data

All manuscripts must include a data availability statement. This statement should provide the following information, where applicable:

- Accession codes, unique identifiers, or web links for publicly available datasets
- A description of any restrictions on data availability
- For clinical datasets or third party data, please ensure that the statement adheres to our policy

The data generated in this study are provided in the main text and Supplementary Information. Source data are provided with this paper.

Research involving human participants, their data, or biological material

Policy information about studies with human participants or human data. See also policy information about sex, gender (identity/presentation), and sexual orientation and race, ethnicity and racism.

Reporting on sex and gender	N/A
Reporting on race, ethnicity, or other socially relevant groupings	N/A
Population characteristics	N/A
Recruitment	N/A
Ethics oversight	N/A

Note that full information on the approval of the study protocol must also be provided in the manuscript.

Field-specific reporting

Please select the one below that is the best fit for your research. If you are not sure, read the appropriate sections before making your selection.

☒ Life sciences ☐ Behavioural & social sciences ☐ Ecological, evolutionary & environmental sciences

For a reference copy of the document with all sections, see nature.com/documents/nr-reporting-summary-flat.pdf

Life sciences study design

All studies must disclose on these points even when the disclosure is negative.

Sample size	Three biological replicates were performed for the key flow cytometry (FC) experiments to confirm the observed effects. For each independent FC experiment at least 100000 single live HEK293T cells were collected for each sample to ensure that coefficient of variation even for target populations with relatively low frequency (~ 1%) is smaller than 5 %. In all experiments for all samples the frequency of target population was > 1%. For ELISA three serial dilutions were measured to determine the concentration of each sample in each replicate. For Western blot analyses five independent experiments were performed. Performing at least three independent experiments provides an opportunity for statistical analysis and is common for the listed assays.
Data exclusions	No data were excluded from the analyses.
Replication	For the key experiments three independent (biological) replicates were performed to confirm the reproducibility. For FC analysis for each sample in each replicate at least 100000 single live HEK293T were collected. Three serial dilutions were measured to determine the concentration of each sample in each replicate using ELISA. Three independent experiments were performed for Western blot analyses. All attempts at replication were successful.
Randomization	The HEK293T cells were identically seeded into wells, then a well was randomly chosen for transfection with particular plasmid set and later all samples were prepared for FC, ELISA, or WB analysis. For FC analysis at least 100000 single live HEK293T cells for each sample in each replicate were collected using the gating strategy (for details see Methods). For FC experiments all selected cells were analyzed to determine median/mean signal of fluorescent proteins or percentage of cells in the frame of interest. Three serial dilutions were measured to determine the concentration of each sample in each replicate using ELISA. All values lying in the range of calibration curve were used to quantify the concentration of samples in each replicate. Three independent experiments were performed for Western blot analyses.
Blinding	The investigators were blinded to group allocation during data collection and analysis.

Reporting for specific materials, systems and methods

We require information from authors about some types of materials, experimental systems and methods used in many studies. Here, indicate whether each material, system or method listed is relevant to your study. If you are not sure if a list item applies to your research, read the appropriate section before selecting a response.

Materials & experimental systems

n/a	Involved in the study
☐	☒ Antibodies
☐	☒ Eukaryotic cell lines
☒	☐ Palaeontology and archaeology
☒	☐ Animals and other organisms
☒	☐ Clinical data
☒	☐ Dual use research of concern
☒	☐ Plants

Methods

n/a	Involved in the study
☒	☐ ChIP-seq
☐	☒ Flow cytometry
☒	☐ MRI-based neuroimaging

Antibodies

Antibodies used	rabbit anti-phospho-eIF2α antibody (Cell Signaling, 3398, dilution 1:1000), mouse anti-total eIF2α antibody (Thermo Fisher Scientific, AHO0802, dilution 1:1000), mouse anti-puromycin antibody (Merck Millipore, clone 12D10, MABE343, dilution 1:1000), mouse anti-cyclophilin B antibody (Abcam, clone CL3901, ab236760, dilution 1:1000), rabbit anti-cyclophilin B antibody (Invitrogen, PA1-027A, dilution 1:1000), IRDye® 800CW goat anti-rabbit antibody (LI-COR, 926-32211, dilution 1:10000), IRDye® 680RD goat anti-mouse antibody (LI-COR, 926-68070, dilution 1:10000), IRDye® 800CW goat anti-mouse antibody (LI-COR, 925-32210, dilution 1:10000), IRDye® 680RD goat anti-rabbit antibody (LI-COR, 926-68071, dilution 1:10000)
Validation	Antibodies were validated by the manufacturers, validation data are provided on a corresponding manufacturer's webpage describing an antibody.

Eukaryotic cell lines

Policy information about cell lines and Sex and Gender in Research

Cell line source(s)	HEK293T (ATCC, CRL-3216), Freestyle™ 293-F (ThermoFischer, R79007)
Authentication	Authenticated by the manufacturer. Validation by morphology.
Mycoplasma contamination	All cell lines were regularly tested for Mycoplasma contamination, with negative results.
Commonly misidentified lines (See ICLAC register)	No misindentified lines were used.

Flow Cytometry

Plots

Confirm that:

☒ The axis labels state the marker and fluorochrome used (e.g. CD4-FITC).

☒ The axis scales are clearly visible. Include numbers along axes only for bottom left plot of group (a 'group' is an analysis of identical markers).

☒ All plots are contour plots with outliers or pseudocolor plots.

☒ A numerical value for number of cells or percentage (with statistics) is provided.

Methodology

Sample preparation	Samples were derived from HEK293T cells as described in the Methods section.
Instrument	LSRFortessa SORP, BD Biosciences
Software	DIVA Software version 9.0.1 for collection, FlowJo version 10.7.1 (BD Biosciences) for analysis.
Cell population abundance	At least 100000 single live HEK293T cells were analyzed for each condition.
Gating strategy	First, the population of HEK293T cells was gated (using FSC-A x SSC-A parameters), and then a single cell population was selected (SSC-W x SSC-A). Next, live cells were picked (SSC-W x 405–450/50 channel). Then the analysis was dependent on the used reporter. All details are presented in the Methods section.

☒ Tick this box to confirm that a figure exemplifying the gating strategy is provided in the Supplementary Information.
